# Effectiveness of community-based complementary food supplement (Yingyangbao) distribution in children aged 6-23 months in poor areas in China

**DOI:** 10.1371/journal.pone.0174302

**Published:** 2017-03-20

**Authors:** Jie Wang, Suying Chang, Liyun Zhao, Wentao Yu, Jian Zhang, Qingqing Man, Li He, Yifan Duan, Hui Wang, Robert Scherpbier, Shi-an Yin

**Affiliations:** 1 Department of Maternal and Child Nutrition, National Institute for Nutrition and Health, Chinese Center for Disease Control and Prevention, Beijing, China; 2 Section of Health and Nutrition and Water, Environment and Sanitation, United Nations Children’s Fund, Beijing, China; 3 Department of Nutrition Surveillance, National Institute for Nutrition and Health, Chinese Center for Disease Control and Prevention, Beijing, China; 4 Department of Nutrition on Aging, National Institute for Nutrition and Health, Chinese Center for Disease Control and Prevention, Beijing, China; 5 Department of Science and Technology, National Institute for Nutrition and Health, Chinese Center for Disease Control and Prevention, Beijing, China; 6 Department of Population Research, China Population and Development Research Center, Beijing, China; Institut de recherche pour le developpement, FRANCE

## Abstract

**Background:**

Poor growth and micronutrient deficiency mainly attack older infants and young children. Some countries have adopted clinically effective measures to combat malnutrition, but the compliance and improvement in efficacy of intervention vehicles in national programs require evaluation.

**Methods:**

Baseline and follow-up cross-sectional surveys were conducted before and after a nutrition intervention program in 3 national poverty counties in China. Soybean-based complementary food supplements called Yingyangbao (YYB) in Chinese and training materials on child feeding were distributed to households with children aged 6–23 months for 18 months. Representative children were selected by probability proportional to size sampling methods to assess compliance of YYB and the intervention efficacy. A questionnaire was designed to collect data on basic characteristics of children, breastfeeding, 24-hour dietary intake, and consumption and appetite of YYB. Anthropometrics and hemoglobin were measured in the field, and anemia prevalence was evaluated. Venous blood was drawn from children aged 12–35 months to evaluate micronutrient status. Logistic regression was used to identify the risk factors for children’s anemia.

**Results:**

Of the children involved in the follow-up survey (n = 693), the *P*50 (*P*25, *P*75) intake of YYB was 6.7 (3.5, 7.0) sachets weekly, and 54.7% of the children liked the taste of YYB. Compared with the baseline situation (n = 823), the proportion of children fed a diverse diet and foods rich in iron or vitamin A increased (*P* < 0.01) in the follow-up study. The prevalence of stunting and underweight decreased (*P* < 0.05), the prevalence of anemia decreased from 28.0% to 19.9% (*P* < 0.01), and the prevalence of vitamin B_12_ deficiency decreased from 26.8% to 15.4% (*P* < 0.01). For children aged 12–23 months, those who liked YYB and consumed 6 or more sachets of YYB weekly were at lower risk for anemia (OR = 0.34, 95% CI 0.13–0.90, *P* < 0.05), but the risk of stunting was associated with a non-diverse diet (OR = 1.48, 95% CI 1.06–2.07, *P* < 0.05).

**Conclusion:**

The quality of diet and nutritional status of children aged 6–23 months are significantly improved by the intervention of YYB and nutrition education, and good compliance to YYB contributes to a low risk for anemia.

**Trial Registration:**

Chinese Clinical Trial Registry ChiCTR-OOC-16008846

## Introduction

Ending all forms of malnutrition by 2030 is one of the targets of the sustainable development goals as reported in the Global Nutrition Report 2015. Older infants and young children are at the greatest risk for malnutrition, mainly in the forms of poor growth and micronutrient deficiency [1]. In 2014, stunting prevalence was 23.8%, and 159 million children under age 5 were still affected by stunting worldwide, with 57% of stunted children living in Asia [2]. Global anemia prevalence in 2010 was 32.9%, and the burden was highest in children under age 5, which was the only age group with negative trends from 1990 to 2010 [3]. In China, stunting affected 19.0% and anemia affected 16.6% of children aged 0–5 years in poorer areas [4]. Furthermore, children under 2 years old in poor areas were at the greatest risk for anemia and poor growth [4–7]. Infants and young children were key targeted populations for combating malnutrition.

Some countries have implemented nutrition intervention programs to improve nutritional status for the targeted populations [1]. Micronutrient supplementation or fortification has been used in recent years instead of pills or capsules in early years to combat anemia or other micronutrient deficiencies in view of the safety of overdose and the sustainability of programs [8–10]. Moreover, micronutrient powders (MNPs), such as Sprinkles [11] and Yingyangbao (YYB, Chinese Child Improvement Program) [10], have been developed as an approach for delivering iron and other micronutrients to young children. MNPs are single-serving packets of vitamins and minerals in powdered form (Sprinkles) or based on soybean or milk powder (YYB) that can be mixed into semisolid food or water before consumption.

The effects of MNP have been tested, and one meta-analysis found that six of ten randomized clinical studies (RCTs) showed the benefits of MNP on anemia, nine of fourteen RCTs suggested a benefit of MNP on hemoglobin, one of two RCTs suggested a benefit of MNP on serum retinol concentration, zero of three studies suggested a benefit of MNP on retinol deficiency, and there was a lack of data regarding the impact on growth [12]. Baseline situations (age and prevalence of malnutrition), MNP composition, intervention duration, and adherence to MNP are the main influential factors for intervention effects. For the intervention study with designed products on a targeted population, the adherence was the only flexible and controllable factor. One study observed that anemia and diarrhea increased with a decrease in adherence [13]. Another study found that anemia decreased by 55.5% in a twice-weekly MNP group and by 66.0% in a daily MNP group [14]. One study compared the adherences and effects of fortified rice and sprinkles and found that 9 of 101 children refused to consume the fortified rice, 30 of 97 children refused to consume sprinkles, and 5 of 94 children did not finish the study in the control group. After 6 months of intervention, the reduction of anemia (defined as <10 g/dL) was 67% in the rice group, 27% in the sprinkle group and 22% in the control group. Therefore, a low compliance with sprinkles could be a reason for the small intervention effect of sprinkles compared with the rice and control groups [15]. Nutrition education could be beneficial for better compliance and dietary quality and could contribute to better impacts of MNP on malnutrition [16–18].

Based on the efficacy of micronutrient products in clinical studies, several countries, including Bangladesh, Mongolia, Bolivia and China, have implemented national MNP programs for infants and young children who are the most susceptible to micronutrient malnutrition [18–22]. However, the adherence, acceptance and effectiveness of MNP programs require further assessment to provide feedback information for the producers to improve their MNP products and for the policy-makers to achieve the best cost-effect outcomes. In the present study, we sought to evaluate the adherence to YYB and efficacy of YYB on child growth, anemia prevalence and malnutrition prevalence of iron, folic acid, vitamin A, vitamin D and vitamin B_12_.

## Subjects and methods

### Study design

We conducted a baseline and follow-up cross-sectional survey in 3 national poverty counties that were selected by the Millennium Development Goals Achievement Fund in China (MDG-China) to evaluate the efficacy of a nutrition intervention program on Children’s malnutrition improvement. In the MDG-China program, soybean-based complementary food supplement called Yingyangbao (YYB) and training materials were distributed free to all households with children aged 6–23 months in the 3 counties, and the intervention lasted for 18 months.

**Fig 1:**
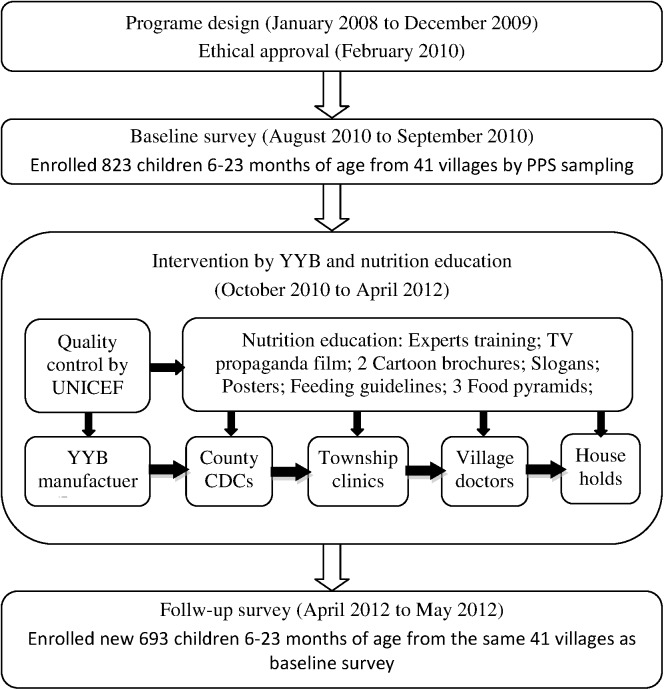
Study flow diagram.

Qingdao Biomate Company supplied YYB under the quality assurance process overseen by UNICEF. The nutrient content of the YYB product was based on China’s national standard GB/T22570-2008 for complementary food supplements. One sachet YYB daily was recommended, and it weighed 12 g and contained 3 g of protein, 7.5 mg of iron, 5 mg of zinc, 200 mg of calcium, 250 μg of vitamin A, 5 μg of vitamin D, 0.5 mg of vitamin B_1_, 0.5 mg of vitamin B_2_, 0.5 μg of vitamin B_12_ and 75 μg of folic acid. YYB and training brochures were provided by UNICEF and distributed by village doctors to households with children aged 6–23 months in the 3 counties for 18 months (Fig 1).

The manufacturer transported YYB to the Center for Disease Control and Prevention (CDC) in each county, the CDCs delivered YYB to township health clinics, and then village doctors came to pick up the YYB and delivered it to families at village clinics. Records were maintained of receipt and delivery at each step. A nutrition education program was also conducted in all related counties, townships and villages to encourage exclusive breastfeeding until 6 months of age and continuous breastfeeding until at least 24 months of age to promote appropriate complementary feeding practices, as well as to guide the feeding of YYB to children. Study staff from the CDCs also trained doctors and nurses in township and village clinics on the relevant YYB guidelines and related nutrition information. Caregivers with children 6–23 months were instructed by those trained village doctors on how to add one sachet of YYB per day to their children’s food. One video about YYB was broadcasted on a local TV channel before and during the program. Two colored cartoon brochures of the YYB manual and an infant feeding guide were provided, giving detailed information on how to use YYB and how to feed children. In total, there were 13,055 children involved in the program, which covered 98.2% of the targeted population. Eleven types of training materials and 58,806 pieces of advocacy materials were distributed among the target areas and families.

### Survey sampling

Forty-one villages were selected from all the target villages in Wuding county, Zhengan county and Zhenan county by probability proportional to size (PPS) sampling methods [23] based on 2010 population estimates, and cluster sampling was utilized for household selection by local CDCs from updated lists of the target households with children aged 6–11, 12–17 and 18–23 months living in those villages. Based on previous studies, the major effect of YYB intervention was to reduce the prevalence of anemia [12]. Therefore, survey sample size estimates were based on a speculated change in anemia prevalence from 30% according to the prescreening result to 20% after the intervention program. The estimated sample size was 817 children at 95% confidence intervals, power of 80%, a design effect of 2.5 and 10% non-response. Baseline and follow-up surveys were conducted in the same villages but with different children.

### Questionnaire

The field survey was conducted by health workers in county medical care centers. Health workers were well trained and supervised by qualified and experienced experts before and during data collection. A questionnaire was designed to collect data on the basic characteristics of the children, consumption and appetite for YYB, breastfeeding and 24-hour dietary intake (WHO IYCF) [24]. Children’s diets were defined as diverse if the children consumed foods from 4 or more of 7 food categories (grains, roots and tubers; legumes and nuts; dairy products [milk, yogurt, cheese]; flesh foods [meat, fish, poultry and liver/organ meats]; eggs; vitamin A-rich fruits and vegetables; and other fruits and vegetables); otherwise, their diets were designated monotonous. The questions were answered by the children’s mothers or their other caregivers.

### Anthropometry

Body weight was measured and recorded to the nearest 0.01 kg with platform weighing scales (TC100KA, 0–100 kg of capacity and 10-g accuracy, Huatec Company, China). Children wore only underwear. Children’s length was measured to the nearest 0.1 cm using a body length scale (YSC-2, Beijing Guowangxingda Weight Scale Company, China). The scales were calibrated before each examination.

### Blood samples for hemoglobin and micronutrient tests

Three to five milliliters of venous blood was drawn from the children aged 12–23 months voluntarily, and one drop of venous blood was used to measure hemoglobin in the field. For younger children and those who did not voluntarily give venous blood, finger blood was used to measure hemoglobin levels by HemoCue Hb 301 (Angelholm, Sweden). Hemoglobin results were similar in finger and venous blood samples [25]. Anemia was defined as hemoglobin < 11 g/dL with adjustment for altitude [26].

Blood samples were covered by aluminum foil to protect them from light. Serum was separated and stored at -20°C at county hospitals and then shipped on ice within one month of fieldwork to Beijing and stored at -70°C until analysis.

Serum retinol was measured by HPLC (Waters 600E). Serum 25-OH-vitamin D was analyzed with the DiaSorin 25-OH-vitamin D ^125^I RIA KIT (Stillwater, Minnesota 55082–0285, U.S.A) and XH6080 Radioimmunoassay (Xi’an Nuclear Instrument Factory, China). Serum ferritin was measured by the ^125^I Ferritin Radioimmunoassay Kit (Beijing North Institute of Biological Technology, China) and XH6080 Radioimmunoassay. Folic acid and vitamin B_12_ were measured by the Simui TRAC-SNB Radioassay Kit (MP Biomedicals LLC, Germany) and XH6080 Radioimmunoassay. C reactive protein was measured by Nanopia CRP (Sekisui Medical Co., Ltd., Japan) using Toshiba 120 Automatic Biochemistry Analyzers (Toshiba, Japan).

Iron deficiency was defined as ferritin levels of less than 12 μg/L and C-reactive protein less than 5 mg/L. Cutoff values were 0.7 μmol/L for vitamin A deficiency and 1.05 μmol/L for vitamin A insufficiency, 50 nmol/L for vitamin D deficiency, 4 ng/mL for folic acid deficiency, and 203 pg/mL for vitamin B_12_ deficiency [7].

### Statistical analysis

Statistical analysis was conducted using SAS software (version 9.1; SAS Institute, Inc., Cary, North Carolina). Data were expressed as the mean ± SD or *P*50 (*P*25, *P*75) for continuous variables and as frequencies for categorical variables. WHO Anthro software was used to calculate the children’s growth status (LAZ, length for age Z score; WAZ, weight for age Z score; WLZ, weight for length Z score; BAZ, BMI for age Z score). Stunting (LAZ < -2), underweight (WAZ < -2), wasting (WLZ < -2) and overweight (BAZ > +2) were estimated [27]. We used the general linear models to compare the means of variances and Chi-square to test the differences in categorical variables between baseline survey results and follow-up survey results, and between groups based on YYB consumption or appetite. For some variables associated with age, we reported the results with age groups of 6–11 months, 12–17 months and 18–23 months. Pearson correlation was used to analyze the associations between factors including growth indicators, hemoglobin and micronutrient levels, intake of YYB and the age of the children. Univariate and multivariate logistic regression were used to assess the potential risk factors for malnutrition, with odds ratios (OR) and 95% confidence intervals (CI) reported. *P* value < 0.05 is considered to be significantly different.

### Ethical review

This project and survey protocol were approved on February 2, 2010, by the Ethical Review Committee of the National Institute for Nutrition and Health at the Chinese Center for Disease Control and Prevention, and the experimental periods would start from March 1, 2010, to December 31, 2012, according to the study protocol. Actually, volunteers were recruited from August 21, 2010, and followed up until May 7, 2012. The questionnaire, anthropometry measurements and blood samples were collected between August 21 and September 9, 2010, for baseline survey and between April 9 and May 7, 2012, for the follow-up survey. Written informed consent was obtained from all children’s parents or their caregivers.

Because the study was to evaluate the efficacy of a Chinese national nutrition intervention program and such study was not considered as a clinical study in the past, we therefore did not register the study before enrollment of participants started. However, we retrospectively registered the study under the title “Improving Nutrition, Food Safety and Food Security for Chinese Most Vulnerable Women and Children” at the Chinese Clinical Trial Registry (Study-ID: ChiCTR-OOC-16008846, date of registration: July 15, 2016, http://www.chictr.org.cn/register.aspx). The authors confirm that all ongoing and related trials for this intervention are registered.

## Results

### Characteristics

Children aged 6–23 months were enrolled at the baseline and final surveys, and their gender and age distribution are shown in Table 1. There were 823 and 693 children aged 6–23 months involved at the baseline and follow-up surveys, respectively. Based on the WHO growth standards, LAZ, WAZ, WLZ, BAZ were -0.79 ± 1.33, -0.56 ± 1.12, -0.21 ± 1.10 and -0.12 ± 1.10 in the baseline survey, and then the scores increased to -0.60 ± 1.27, -0.24 ± 0.97, 0.08 ± 1.07 and 0.17 ± 1.13 in the follow-up survey, respectively (*P* < 0.05).

**Table 1:** Characteristics of the children in the baseline and follow-up surveys.

Characteristics	Baseline	Follow-up	F/chisq	P
Total number surveyed	823	693		
Average age (months)	14.5 ± 5.1	15.7 ± 4.9	21.68	<0.01
Age group (months)	N (%)	N (%)		
6–11	308 (37.4)	198 (28.6)	13.26	<0.01
12–17	272 (33.0)	230 (33.2)	0.003	0.95
18–23	243 (29.5)	265 (38.2)	12.82	<0.01
Gender	N (%)	N (%)		
Boy	428 (52.0)	386 (55.7)	2.07	0.15
Girl	395 (48.0)	307 (44.3)		

### Feeding practices in the baseline and follow-up surveys

Based on the WHO IYCF, the feeding practices of the children were evaluated and are shown in Table 2. The proportions of children fed on diverse diets and foods rich in iron or vitamin A in the follow-up survey were significantly higher than in the baseline survey (*P* < 0.01). No difference was shown in breastfeeding indicators between the two surveys (*P* > 0.05).

**Table 2:** Feeding practices of children in the baseline and follow-up surveys (%).

Feeding indicators	Baseline	Follow-up	Chisq	P
Children ever breastfed	90.9	91.6	0.24	0.62
Continued breastfeeding at 1 year	41.7	41.1	0.01	0.92
Continued breastfeeding at 2 years	10.4	12.0	0.22	0.64
Introduction of solid, semi-solid or soft foods	75.5	75.6	0.00	0.99
Minimum dietary diversity ^1^	46.1	58.4	23.13	<0.01
Consumption of iron-rich or iron-fortified foods ^2^	40.6	47.5	7.26	<0.01
Consumption of vitamin A-rich foods and supplements ^2^	70.0	82.5	32.24	<0.01

^1^ The proportion of children 6–23 months of age who receive foods from 4 or more food groups. The seven food groups include grains, roots and tubers; legumes and nuts; dairy products (milk, yogurt, cheese); flesh foods (meat, fish, poultry and liver/organ meats); eggs; vitamin-A rich fruits and vegetables; and other fruits and vegetables according to WHO IYCF.

^2^ Not including YYB.

### Consumption of and appetite for YYB

YYB consumption and appetite are shown in Table 3. For the children involved in the follow-up survey, the *P*50 (*P*25, *P*75) intake of YYB was 6.7 (3.5, 7.0) sachets weekly; moreover, 22.9% consumed 2 or less sachets of YYB weekly, 23.1% consumed 3 to 6 sachets of YYB weekly, and 54.0% consumed 7 sachets of YYB weekly. A total of 54.7% of the children liked the taste of YYB. Age could have affected the intake of and appetite for YYB, and a larger proportion of older children liked YYB and consumed more than younger children (*P* < 0.05).

**Table 3:** Yingyangbao consumption weekly by children of different age groups [N(%)].

Age group (months)	YYB consumption weekly		Appetite for YYB	
	< 6 sachets	> = 6 sachets	Dislike	Like
6–11	104 (52.5)	94 (47.5) ^a^	81 (55.5)	65 (44.5) ^a^
12–17	79 (34.3)	151 (65.7) ^b^	86 (46.0)	101 (54.0) ^a^
18–23	74 (37.9)	191 (72.1) ^b^	84 (38.0)	137 (62.0) ^b^
Average	257 (37.1)	436 (62.9)	251 (45.3)	303 (54.7)

^a^ Values in the same column without the same superscript letter are different at the *P* < 0.05 level.

^b^ Values in the same column without the same superscript letter are different at the *P* < 0.05 level.

### The prevalence of malnutrition in the baseline and follow-up surveys

The prevalence of growth malnutrition by age group is listed in Table 4. The prevalence of stunting and underweight for children aged 12–17 months and/or 18–23 months in the follow-up survey was lower than in the baseline survey (P = 0.054 or *P* < 0.05). However, more children aged 18–23 months in the follow-up survey were affected by overweight than in the baseline survey (*P* < 0.05).

**Table 4:** Growth status of children in the baseline and follow-up surveys for different age groups (%).

	Age group (months)	Stunting	Underweight	Wasting	Overweight
Baseline	6–11	11.7	6.5	3.2	4.2
	12–17	19.5	12.9 ^a^	8.5 ^a^	2.2
	18–23	24.3 ^a^	9.0 ^a^	4.9	0.8 ^a^
	Average	18.0 ^a^	9.4 ^a^	5.5	2.6 ^a^
Follow-up	6–11	10.6	3.0	3.5	5.0
	12–17	13.0	3.9 ^b^	2.6 ^b^	3.5
	18–23	11.7 ^b^	4.2 ^b^	4.5	5.3 ^b^
	Average	11.8 ^b^	3.8 ^b^	3.6	4.6 ^b^

^a^ Values in the same age group without the same superscript letter are different at the *P* < 0.05 level.

^b^ Values in the same age group without the same superscript letter are different at the *P* < 0.05 level.

The means of hemoglobin, retinol, ferritin, folic acid and vitamin B_12_ concentrations were significantly improved from 117.1±12.0 g/L, 1.04±0.16 μmol/L, 17.8±11.9 μg/L, 13.1±7.3 μg/L and 313.0±149.9 ng/L in the baseline, respectively, to 119.8±11.1 g/L, 1.27±0.35 μmol/L, 20.4±14.2 μg/L, 15.7±7.6 μg/L and 494.7±292.5 ng/L in the follow-up survey (*P* < 0.05). However, the mean concentration of vitamin D significantly decreased from 88.3±22.0 nmol/L to 56.6±20.2 nmol/L (*P* < 0.05).

The prevalence of micronutrient malnutrition is shown in Table 5. For children aged 12–17 months and 18–23 months, the prevalences of anemia, vitamin A insufficiency and vitamin B_12_ deficiency were lower in the follow-up survey than in the baseline survey (*P* < 0.05), and the prevalences of iron deficiency and folic acid deficiency were similar in the the baseline and follow-up surveys (*P* > 0.05), but the prevalence of vitamin D deficiency was higher in the follow-up survey than in the baseline survey (*P* < 0.05).

**Table 5:** Prevalence of micronutrient malnutrition in the baseline and follow-up surveys for different age groups of children (%).

	Age group (months)	Anemia	Vitamin A insufficiency	Vitamin D deficiency	Iron deficiency	Folic acid deficiency	Vitamin B_12_ deficiency
Baseline	N	823	219	177	264	149	110
	6–11	38.3	-	-	-	-	-
	12–17	26.1 ^a^	57.9 ^a^	1.1 ^a^	43.1	1.3	32.2
	18–23	16.9 ^a^	53.6 ^a^	5.9 ^a^	29.1	2.8	19.6
	Average	28.0 ^a^	55.7 ^a^	3.4 ^a^	35.6	2.0	26.4 ^a^
Follow-up	N	693	380	370	366	373	372
	6–11	36.9	-	-	-	-	-
	12–17	18.7 ^b^	31.2 ^b^	40.4 ^b^	40.4	0.0	22.3
	18–23	8.3 ^b^	29.6 ^b^	41.6 ^b^	27.9	0.0	10.2
	Average	19.9 ^b^	30.3 ^b^	41.1 ^b^	33.1	0.0	15.3 ^b^

^a^ Values in the same age group without the same superscript letter are different at the *P* < 0.05 level.

^b^ Values in the same age group without the same superscript letter are different at the *P* < 0.05 level.

- No data reported due to a lack of venous blood samples.

### The effect of YYB consumption on growth and micronutrient status

The effect of YYB intake and appetite on micronutrient status is shown in Table 6. The children involved in the follow-up survey were divided into two groups according to YYB consumption weekly or appetite for YYB. We did not observe that growth malnutrition was affected by either YYB sachets weekly or appetite for YYB (*P* > 0.05). However, the children who took 6 or more sachets of YYB weekly were less affected by anemia (*P* < 0.05), vitamin A insufficiency (*P* > 0.05) and iron deficiency (*P* = 0.06) than the children who took less than 6 sachets of YYB weekly. Furthermore, the children who liked YYB had less prevalence of anemia, vitamin A insufficiency and iron deficiency than the children who disliked the taste of YYB (*P* < 0.05). We did not observe the influence of intake of and appetite for YYB on the status of vitamin D and vitamin B_12_.

**Table 6:** Micronutrient status affected by intake of and appetite for YYB (%).

Nutritional status	Intake		Appetite	
	< 6 sachets	> = 6 sachets	Dislike	Like
N	257	436	251	303
Stunting	12.1	11.7	11.2	10.6
Underweight	2.3	4.6	5.6	3.6
Wasting	2.0	4.6	6.0	3.0
Overweight	4.3	4.8	4.0	4.0
Anemia	28.4 ^a^	14.9 ^b^	20.7 ^a^	13.2 ^b^
Vitamin A insufficiency ^1^	34.2	28.6	36.1 ^a^	23.8 ^b^
Vitamin D deficiency ^1^	41.4	40.7	43.0	39.1
Iron deficiency ^1^	40.0	30.0	38.0 ^a^	27.1 ^b^
Vitamin B_12_ deficiency ^1^	17.4	14.6	12.7	13.9

^a^ Values without the same superscript letter are different at the *P* < 0.05 level.

^b^ Values without the same superscript letter are different at the *P* < 0.05 level.

^1^ Results from the children aged 12–23 months.

In the multivariate logistic model for children aged 12–23 months, those who liked YYB and consumed 6 or more sachets of YYB weekly were at lower risk for anemia than the children who disliked YYB and consumed less than 6 sachets of YYB weekly (OR = 0.34, 95% CI 0.13–0.90, *P* < 0.05 after adjusting for age and gender). The risk of stunting was only associated with a non-diverse diet (OR = 1.48, 95% CI 1.06–2.07, *P* < 0.05 after adjusting for age and gender) but not with either intake of or appetite for YYB.

## Discussion

Malnutrition and inappropriate feeding practices during the first two years of life have been shown to increase susceptibility to infection, which results in morbidity and mortality, and to adversely affect a child’s growth and development. Food-based nutrition intervention programs have been shown to effectively improve nutritional status for the targeted populations. However, the adherence or acceptance to the intervention product would be an important factor affecting the food-based intervention efficacy. Our study showed that the quality of diet and nutritional status of children aged 6–23 months were improved by the intervention of YYB and nutrition education and that better adherence to YYB contributes to a greater efficacy of intervention.

Previous studies seldom reported the beneficial effect of MNP on growth and development of children [12]. However, we found that the prevalence of stunting decreased from 18.0% to 11.8% and that the prevalence of underweight decreased from 9.4% to 3.8% after 18 months of YYB intervention together with nutrition education. By comparing the feeding practices between the baseline and follow-up surveys, a higher percentage of children consumed diverse diets and iron-fortified or vitamin A-rich foods at the end of the study compared to at baseline. The risk of stunting was associated with a monotonous diet (OR = 1.48, 95% CI 1.06–2.07, *P* < 0.05 after adjusting for age and gender) but not with either intake of or appetite for YYB. One previous study also observed that a diverse diet was a protective factor against anemia in young children [7].

Comparing the MNP involved studies, it has been found that body weight and children’s length were improved in studies that involved nutrition education [15, 28] but not in the studies with MNP without nutrition education[14, 29–31]. The intervention of only nutrition education without supply of food or supplements can also improve the growth and development of children [32]. Thus, only micronutrient supplementation without nutrition education or diet intervention would not improve the growth status in infants and young children in poor areas. Therefore, we speculated that the reduction of stunting and underweight in the present study benefited more from the improvement in diet quality through nutrition education than from YYB intervention.

Micronutrient supplementation has improved hemoglobin concentration and decreased anemia prevalence in several studies. Overall, after 2 months or longer intervention, a significant rise in hemoglobin levels (3–16 g/L) can be achieved, and a mean reduction of 34% of anemia was found [12]. Among the studies, lower hemoglobin concentration (100 g/L and lower) or higher proportions of anemia at baseline (50–80%) in young children [18, 33–35] and good adherence of children in the intervention group [14, 36] may lead to the greater improvement in hemoglobin levels and anemia prevalence. The present study found a 2.7 g/L rise in hemoglobin concentration and 28.9% reduction in anemia prevalence after 18 months of YYB intervention together with nutrition education. The intervention impact that resulted from the study was more effective than a previous study in a similar situation in which a 20% reduction in anemia was achieved [16]. Nutrition education on the benefits of YYB and child feeding practices may also contribute to the good adherence and dietary quality improvement, which leads to benefits in the malnutrition improvement.

After YYB intervention accompanied by nutrition education, we observed significant improvement in vitamin A, ferritin, folic acid, and vitamin B_12_ concentrations in serum. Several previous studies have found similar beneficial effects of MNP on vitamin A and ferritin [34, 35, 37, 38], but no studies indicated the effect of MNP or YYB on folic acid, vitamin B_12_ and vitamin D. It is necessary to conduct more studies to evaluate the efficacy of multi-micronutrient supplementation on micronutrient status. It is well known that exposure to sunlight can induce vitamin D synthesis in skin, and vitamin D status is mainly influenced by season [39]. The baseline survey was conducted in summer and the final survey was conducted in spring in the present study, which may mainly contribute to the difference in vitamin D status. Therefore, it is necessary to conduct the baseline and final surveys in the same season to assess the effect of supplementation on vitamin D status. At the same time, our results also indicated that the fortified dose (5 μg/d) of vitamin D in the intervention product was not enough to maintain the optimal vitamin D status.

Our study results showed that median intake of YYB was 6.7 sachets weekly, and two-thirds of children consumed one sachet of YYB daily. When comparing the intervention effect, those children who took 6 or more sachets of YYB weekly had a 47.5% lower prevalence of anemia than the children who took less than 6 sachets of YYB weekly. One previous study found that when the proportion of children who used Sprinkles in the previous 7 days decreased from 64.9% in 2008 to 21.9% in 2010, together with an average intake of 3.2 sachets weekly in 2008 decreasing to 1.1 sachets weekly in 2010, the prevalence of anemia increased from 42.8% in 2008 to 71.7% in 2010 [13]. Therefore, good compliance is the foundation for achieving optimal efficacy through the intervention program. Improving YYB flavor may lead more children to like the taste of YYB and thus would contribute to better compliance and intervention efficacy.

The status of vitamin D in humans is more sensitive to sunlight exposure than food supplements, but the baseline survey was conducted in summer and the follow-up survey was done in spring; therefore, we did not find an effect of YYB on the improvement of vitamin D status, which requires further study to evaluate. Venous blood was drawn from children over 1 year old, and we did not observe whether the YYB intervention improved infants’ micronutrient status. The intervention benefits obtained from this intervention program were synergistically benefited from both YYB and nutrition education; however, we could not speculate on the contribution of the education effect because the study did not include a group of children who consumed YYB without nutrition education.

## Conclusions

Micronutrient supplements (Yingyangbao) together with nutrition education significantly improve dietary quality and decrease the prevalence of stunting, underweight, anemia, vitamin A insufficiency and vitamin B_12_ deficiency in children.

## Supporting information

S1 File Trend checklist. (DOCX)

S2 File Study protocol. (DOCX)

S3 File Yingyangbao intake, child feeding practices, growth status and micronutrient status in children. (DOCX)
