# Supplementary material for: Effectiveness of community-based complementary food supplement (Yingyangbao) distribution in children aged 6-23 months in poor areas in China
Source: PLoS One. 2017 Mar 20;12(3):e0174302. doi: 10.1371/journal.pone.0174302 (PMC5358851; doi:10.1371/journal.pone.0174302)
Supplement: S2 File — (DOCX) [file pone.0174302.s002.docx]

**Improving Nutrition, Food Safety and Food Security for Chinese Most Vulnerable Women and Children**

**Study Protocol**

Funding information：Millennium Development Goals Spanish Achievement Fund

**1. Background**

Nutrition and health in early life were the basis of a healthy life, and the first 1000 days of life were the most important time in the life. Therefore, nutrition of the women at childbearing age and the children under 2 years old should be focused.

Recent surveys showed that the nutritional status of women and children in rural areas in China need to be improved. The prevalence of stunting and low weight for children under 5 years old in rural areas was 5.3 times and 4.6 times higher than in urban areas in 2006. In a 2005 national survey on children, it was reported that the children under 5 years old in China was affected by anemia with 1.9 times higher in rural than in urban areas.

Improving nutritional status of women and children will benefit to the society’s sustainable development. Considering the poor economy situation, less knowledge on nutrition and diets, sort of resource or waste of local foods rich in nutrients, we carried out the nutrition survey and intervention project in national three poorest counties.

Food safety in China was analyzed by WFP, FAO and UNIDO and 271 poverty counties were defined. Three of them were selected to carry out the nutrition survey and intervention study, and they were Zhengan County in Guizhou Province, Wuding County in Yunnan Province, AND Zhenan County in Shanxi Province. The nutritional status of children, pregnant women and lactating women will be analyzed, nutrition intervention will be done on the children, nutrition knowledge on dietary balance and infant and young children feeding practices will be trained on the village doctors and women to improve the nutrition status on these population.

**2. Study aims**

To understand the nutrition status of children and women, to carry out nutrition intervention program on children aged 6-23 months by complementary food supplements (Yingyangbao, YYB), to improve nutrition and child feeding skills of caregivers and women in child-bearing age, and to collect experience of YYB distribution.

**3. Study design**

## Target population: pregnant women, breast-feeding mothers, and children aged 0-35 months at 3 poor counties

## Baseline nutrition survey for pregnant and breastfeeding women and children

## Intervention by Yingyangbao (YYB) and education on all children aged 6-23 months (around 15000 children) living in the 3 counties for about 18 months

## Final nutrition survey on children aged under 3 years

Doctors participated into this project will get trained on nutrition knowledge and child feeding skills, YYB and training materials were distributed among households with children aged 6-23 months. Doctors visit households and give face-to-face guidance at least once a month. Advocacy slogans and videos can be seen on local areas and TV channel.

The same villages as baseline nutrition survey

Representative women and children selected by PPS and cluster sampling method at target sites

## To understand the nutrition situation of children after intervention by YYB and nutrition education

## To understand the nutrition situation of women and children

Analysis: to understand the nutrition situation of women and children in poor areas and to evaluate the nutrition intervention effect of complementary food supplements (Yingyangbao) plus education in Children aged 6-23 months in Poor Areas in China

**Figure 1. Study flow diagram**

**3.1 Baseline nutrition survey**

**3.1.1 Sampling and sample size**

Cross-section survey and PPS sampling methods were used in this study. Three counties were selected and representative for poverty counties in western China, Zhengan in Guizhou province, Wuding in Yunnan province and Zhenan in Shanxi province. Sixteen villages from Wuding County, 16 villages from Zhenan County and 9 villages from Zhengan County were selected into our cross-section survey by PPS sampling principles based on 2010 population estimates. Cluster sampling was utilized for household selection.

A total of 817 children were theoretically estimated to be adequate to detect a change of anemia prevalence from 30 % at baseline to 20% at follow-up survey, with 80 % power, 95 % confidence intervals, a design effect of 2.5 and 10 % non-response.

**3.1.2 Volunteers’ recruitment**

Advocacy and communication on this nutrition survey and intervention will be advertised in all villages. The contents of nutrition survey, nutrition intervention and evaluation will be fully understood by the parents and the pregnant or lactating women. The parents with a child age 0-3 years will be informed on the nutrition survey and intervention, if they want to participate in the survey or receive the Yingyangbao, they will sign the informed consent form. The pregnant and lactating women who voluntarily join the nutrition survey will also sign the informed consent.

**3.1.3 Survey indicators and data collection**

**Anthropometry**

Body weight was measured and recorded to the nearest 0.01 kg with platform weighing scales (TC100KA, 0-100 kg of capacity and 10 g of accuracy, Huatec Company, China). Children wore only underwear. The length of children less than 24 months of age and the height of children older than 24 months were measured to the nearest 0.1 cm using a body length scale (YSC-2, Beijing Guowangxingda Weight Scale Company, China) and a body height scale (TZG, Jiangyin Medical Equipment Company, China). The scales were calibrated before each examination.

**Blood samples collection and analysis**

Three to five milliliters of blood were drawn from the children aged 12-35 months, breastfeeding mothers and pregnant women, and one drop of venous blood was used to measure hemoglobin at the field site. Blood samples were covered by aluminum foil to protect them from light. Serum was separated and stored at -20℃ at county hospitals, then shipped on ice within one month of fieldwork to Beijing and stored at -70℃ until analysis.

For children younger than one year old or for participants who did not voluntarily give venous blood, finger blood was used to measure hemoglobin level by HemoCue 301 (Angelholm, Sweden). Anemia was defined as hemoglobin < 11 g/dL for children aged 6-35 months and < 12 g/dL for the mothers, and all the cutoffs were adjusted for altitude. Because there was no hemoglobin cut-off for children younger than 6 months, we used the same cut-off as for children aged 6-35 months to assess anemia.

Serum retinol was measured by HPLC (Waters 600E). Serum 25-OH-vitamin D was analyzed with the DiaSorin 25-OH-vitamin D ^125^I RIA KIT (Stillwater, Minnesota 55082-0285, U.S.A) and XH6080 Radioimmunoassay (Xi’an Nuclear Instrument Factory, China). Serum ferritin was measured by the ^125^I Ferritin Radioimmunoassay Kit (Beijing North Institute of Biological Technology, China) and XH6080 Radioimmunoassay. Folic and vitamin B_12_ were measured by the Simui TRAC-SNB Radioassay Kit (MP Biomedicals LLC, Germany) and XH6080 Radioimmunoassay. C reactive protein was measured by Nanopia CRP (Sekisui Medical Co., Ltd., Japan) using Toshiba 120 Automatic Biochemistry Analyzers (Toshiba, Japan).

Iron deficiency was defined as ferritin levels of less than 12 μg/L, and the analysis was restricted to children with C-reactive protein of less than 5 mg/L. Cutoff values were 0.7 μmol/L for vitamin A deficiency and 1.05 μmol/L for vitamin A insufficiency, 50 nmol/L for vitamin D deficiency, 4 ng/mL for folic acid deficiency, and 203 pg/mL for vitamin B_12_ deficiency.

**Questionnaire**

A questionnaire including baby’s birth status, family information, and disease history and feeding practices according to WHO IYCF was designed to collect these information. IYCF knowledge covering breastfeeding and complementary feeding will be answered by the pregnant and lactating women. In addition, health status during pregnancy and 24 hour diet record will be recalled by the women.

**3.2 Nutrition intervention**

**3.2.1 Yingyangbao intervention**

Yingyangbao (YYB) was designed according to General standard for complementary food supplements (GB/T22570-2008) to increase nutrient intake (vitamin A, D, B1, B2 B12, folic, calcium, iron, zinc) in infant and young children. In this project, YYBs will be distributed to around 15000 children aged 6-23 months in Wuding, Zhenan and Zhengan counties within 18 months from Oct 2010 to Mar 2012. Caregivers can receive YYB from village doctors and feed them to the babies under the guidance of doctors. Record cards will be used to write down the consumption of Yiangyangbao by caregivers. One achets YYB daily is recommended, and the intervention will last for 18 months.

Yingyangbao distribution:

Manufacturer

Every 2 months

County CDC

Within 1 week and every 2 months

Township hospital

Within 1 week in each month

Village doctors

Within 1 week in each month

Caregivers

**3.2.2 Purchase and supplies of yingyangbao**

UNICEF takes charge of Yingyangbao pruchasement including supplier and price. The manufacturer transports the Yingyangbao to County CDC. National Institute for Nutrition and Food Safety provides the quantity of Yiangyangbao based on target children to UNICEF.

**3.2.3 Quality control of yingyangbao**

UNICEF and manufacturer take charge of quality of Yingyangbao. The manufacturer should have process quality control and product test to prove the quality. The quality control plan is listed in Table 1.

**Table 1 Yingyangbao quality control plan**

| Sampling site | Time schedule | Samples |
| --- | --- | --- |
| Producer | Oct, 2010 | 3batches×2bags/batch×30sachets/bag |
| 1）3 Counties | Oct, 2010 and Apri, 2011 | 1 batch×1bag/batch×30sachets/bag |
| 2）2 Village doctors | Oct, 2010 and Apri, 2011 | 1 batch×1bag/batch×30sachets/bag |
| 3）2 households in each county | Oct, 2010 and Apri, 2011 | 6 sachets from household |

**3.2.4 Yingyangbao storage**

Yingyangbao should be stored in safety place and avoid high moisture, poisonous material, drugs and insects.

**3.2.5 Advocacy**

Training materials and advocacy materials were spread among the target areas and families (Table 2). Communication meetings and training lectures to doctors and caregivers were held in each county to understand the importance of children’s nutrition and health, the benefit of YYB, breastfeeding and complementary skills. The village doctors visited the households to teach the parents or caregivers to feed YYB on children according to the Yingyangbao introduction books and feeding brochures.

At all villages, people could see advocacy slogans of YYB and child nutrition which could mind the parents to feed their children on YYB. When people went to hospitals or clinics, dietary guidance and food pyramid posters of women and children could be seen. During the first several months, an advocacy DVD “The love of mother” was broadcasted on local TV channel in which the benefit of YYB was easy to be understood.

**Table 2 Training materials and targeted population**

| Items | Acceptors or posting places |
| --- | --- |
| Lectures | Doctors at townships and villages |
| Home-visit and face-to-face | All children’s caregivers |
| Yingyangbao introduction books | All children’s caregivers |
| Infant and young children feeding brochures | All children’s caregivers |
| Dietary guidance on Chinese pregnant, lactating women and children aged 0-6 years | All doctors at county, township and village levels |
| Posters of food pyramid for children | Hospitals or clinics at townships and villages |
| Posters of food pyramid for pregnant and lactating women | Hospitals or clinics at townships and villages |
| Posters of breastfeeding, complementary feeding | Hospitals or clinics at townships and villages |
| Advocacy slogans | Outside of townships and villages |
| Advocacy on blackboard | Townships and villages |
| Advocacy DVD | Local TV channel |

**3.3 Final nutrition survey (evaluation on nutrition intervention)**

After the nutrition intervention, a final nutrition survey will be carried on the children aged 0-3 years, the contents are the same as the baseline nutrition survey so that the nutrition intervention effect could be evaluated. Moreover, intake and appetite for Yingyangbao was assessed in the final survey.

**4 Quality control**

The field survey was conducted by health workers in county medical care centers. Health workers were well trained and supervised by qualified and experienced experts before and during data collection. The same vehicles and materials were used in 3 counties and provided by NINFS at China CDC, and NINFS analyze all the blood samples in same method for the same biomarkers with quality control sample used during the laboratory analysis. Double data entry was used to control errors.

**5 Data input and analysis**

Epidata 3.1 was used to double data entry. Statistical analysis was conducted using SAS software (version 9.1; SAS Institute, Inc., Cary, North Carolina). Data were expressed as the mean ± SD or median (range) for continuous variables and as frequencies for categorical variables. WHO Anthro software was used to calculate the children’s growth status (HAZ/LAZ, height/length for age Z score; WAZ, weight for age Z score; WHZ/WLZ, weight for height/length Z score; BAZ, BMI for age Z score). Stunting (HAZ/LAZ < -2), low weight (WAZ < -2), wasting (WHZ/WLZ < -2) and overweight (BAZ > +2) were estimated. We used the general linear models and Chi-square test to compare the means of variances and the differences in contingency tables. Univariate logistic regression was used to analyze the potential risk factors for anemia; the risk factors were then fed into stepwise multivariate logistic regression models to assess the odds ratios (OR) and 95% confidence intervals (CI). *P* value < 0.05 is considered to be statistically significantly different.

**6. Time schedule**

| Year  contents | 2010 | | | | | | | | | | | | | 2011 | | | | | | | | | | | | 2012 | | | | | | | | | | | | |
| --- | --- | --- | --- | --- | --- | --- | --- | --- | --- | --- | --- | --- | --- | --- | --- | --- | --- | --- | --- | --- | --- | --- | --- | --- | --- | --- | --- | --- | --- | --- | --- | --- | --- | --- | --- | --- | --- | --- |
| Month of the year | 1 | 2 | 3 | 4 | 5 | 6 | 7 | 8 | 9 | 10 | 11 | 12 | 1 | | 2 | 3 | 4 | 5 | 6 | 7 | 8 | 9 | 10 | 11 | 12 | | 1 | 2 | 3 | 4 | 5 | 6 | 7 | 8 | 9 | 10 | 11 | 12 |
| Study design |  |  |  |  |  |  |  |  |  |  |  |  |  | |  |  |  |  |  |  |  |  |  |  |  | |  |  |  |  |  |  |  |  |  |  |  |  |
| Communication with target provinces and counties |  |  |  |  |  |  |  |  |  |  |  |  |  | |  |  |  |  |  |  |  |  |  |  |  | |  |  |  |  |  |  |  |  |  |  |  |  |
| Advocacy material design |  |  |  |  |  |  |  |  |  |  |  |  |  | |  |  |  |  |  |  |  |  |  |  |  | |  |  |  |  |  |  |  |  |  |  |  |  |
| Yingyangbao purchasement |  |  |  |  |  |  |  |  |  |  |  |  |  | |  |  |  |  |  |  |  |  |  |  |  | |  |  |  |  |  |  |  |  |  |  |  |  |
| Initiation and training |  |  |  |  |  |  |  |  |  |  |  |  |  | |  |  |  |  |  |  |  |  |  |  |  | |  |  |  |  |  |  |  |  |  |  |  |  |
| Provincial initiation meeting |  |  |  |  |  |  |  |  |  |  |  |  |  | |  |  |  |  |  |  |  |  |  |  |  | |  |  |  |  |  |  |  |  |  |  |  |  |
| Baseline nutrition survey |  |  |  |  |  |  |  |  |  |  |  |  |  | |  |  |  |  |  |  |  |  |  |  |  | |  |  |  |  |  |  |  |  |  |  |  |  |
| Yingyangbao production |  |  |  |  |  |  |  |  |  |  |  |  |  | |  |  |  |  |  |  |  |  |  |  |  | |  |  |  |  |  |  |  |  |  |  |  |  |
| Yingyangbao distribution |  |  |  |  |  |  |  |  |  |  |  |  |  | |  |  |  |  |  |  |  |  |  |  |  | |  |  |  |  |  |  |  |  |  |  |  |  |
| Final nutrition survey |  |  |  |  |  |  |  |  |  |  |  |  |  | |  |  |  |  |  |  |  |  |  |  |  | |  |  |  |  |  |  |  |  |  |  |  |  |
| Nutrition advocacy |  |  |  |  |  |  |  |  |  |  |  |  |  | |  |  |  |  |  |  |  |  |  |  |  | |  |  |  |  |  |  |  |  |  |  |  |  |
| Mid-term estimation |  |  |  |  |  |  |  |  |  |  |  |  |  | |  |  |  |  |  |  |  |  |  |  |  | |  |  |  |  |  |  |  |  |  |  |  |  |
| Provincial supervision |  |  |  |  |  |  |  |  |  |  |  |  |  | |  |  |  |  |  |  |  |  |  |  |  | |  |  |  |  |  |  |  |  |  |  |  |  |
| Data analysis and report writing |  |  |  |  |  |  |  |  |  |  |  |  |  | |  |  |  |  |  |  |  |  |  |  |  | |  |  |  |  |  |  |  |  |  |  |  |  |
| Communication other partners of MDG program |  |  |  |  |  |  |  |  |  |  |  |  |  | |  |  |  |  |  |  |  |  |  |  |  | |  |  |  |  |  |  |  |  |  |  |  |  |
| Final meeting |  |  |  |  |  |  |  |  |  |  |  |  |  | |  |  |  |  |  |  |  |  |  |  |  | |  |  |  |  |  |  |  |  |  |  |  |  |

改善中国最弱势妇女和儿童群体的营养、食品安全和食品保障状况

研究方案

项目来源：联合国千年发展目标西班牙基金

**1. 项目背景**

婴幼儿时期是大脑和身体各组织器官发育的关键时期，婴幼儿时期的健康状况可能会影响儿童的一生以及成年时期慢性疾病发生的易感性。儿童营养不良可开始于生命的最初阶段，尤其是宫内营养不良可直接影响婴儿的发育，因此关注儿童的营养与健康状况的改善应从关注育龄妇女的健康状况开始。

近年来全国性调查结果显示，我国农村妇女和儿童的营养与健康状况丞待改善，以不发达地区尤为突出。据估计全世界发育不良的儿童中有720万人（约占4%）在中国。2005年，农村地区低体重儿童数量（10%）是城市低体重儿童的5倍（2%），儿童贫血率平均为19.3%，贫血率在最为贫困的县可高达80%。2002年全国营养与健康状况调查结果显示，约半数农村的儿童（49.5%）处于维生素A边缘缺乏状态。我国是农业大国，解决农村和农民的问题是是我国政府所面临的重大问题，改善农村妇女和儿童的营养与健康状况将有利于国家的长期可持续发展所需要后备人才的储备。

然而，由于贫困地区经济落后，居民的购买能力差，同时在这些地区也存在资源短缺和因知识匮乏造成的资源浪费问题，贫困地区需要更多的营养性食品、强化食品，而儿童的看护人也渴望得到更多的营养知识来科学合理喂养儿童的同时，改善自身营养与健康状况。

联合国世界粮食计划署、联合国粮农组织及国际农业发展基金联合开展了对中国各地粮食安全现状的文献和二手数据的调研，确定了271个中国最贫困县，本项目从中挑选了3个最贫困县开展调查和干预工作，分别是：贵州省正安县、陕西省镇安县、云南省武定县。调查贫困县0-3岁儿童、孕妇、乳母营养状况，对6-24月龄儿童进行微量营养素补充，评价营养干预效果。同时，对儿童家长、孕妇、乳母进行营养知识和儿童科学喂养知识培训，通过补充干预和宣传教育等措施，改善妇女和儿童的营养与健康状况。

**2. 项目目标**

2.1 了解贫困地区儿童和妇女营养状况。

2.2 采用婴幼儿辅食营养补充品（营养包）对6-23月龄的儿童进行干预，改善儿童的营养与健康状况。

2.3 高婴幼儿家长和育龄妇女的营养知识，通过科学喂养和合理搭配膳食改善儿童和育龄妇女营养与健康状况。

2.4 在项目实施过程中，探讨并解决营养包发放体系和有效机制，为今后营养干预积累经验。

**3. 项目实施方案**

## 目标人群：孕妇、乳母和婴幼儿

## 针对6-23月龄儿童进行营养干预（营养包+宣传教育）

## 对婴幼儿开展终期营养调查

## 基线营养状况调查

## （孕妇、乳母和婴幼儿）

在与基线调查相同的样本村对婴幼儿进行营养调查

对于6-23月龄的儿童，采用营养包与家长营养知识宣传教育相结合的方法进行营养干预，乡村医生将得到专业培训，乡村医生每月至少一次入户走访和指导营养包使用和婴幼儿喂养，宣传材料发放到婴幼儿家庭中，宣传标语和宣传电视节目可以在当地收看。

PPS法选取样本村，调查样本村内的妇女和儿童的营养与健康状况

## 了解营养干预后的儿童营养与健康状况及营养包服用情况

## 了解妇女和儿童的营养状况

分析：妇女与儿童的营养与健康状况，评价营养干预对婴幼儿营养与健康状况的改善效果

**图1. 研究流程**

**3.1 基线营养状况调查：**

**3.1.1 抽样与样本量：**

基于每个县的目标人群，按PPS抽样原则，将目标县中所有自然村按总人口数排序，并计算累计人口数。确定样本村数，计算抽样间隔，确定样本村。武定县和镇安县各抽取16个村、正安县抽取9个村进行营养状况调查。估计干预前儿童贫血患病率30%，期望干预后患病率降低30%，取α=0.05，β=0.2，设计效应1.5，拒绝率10%，则根据样本量计算公式得到的监测儿童样本量为600人。

**3.1.2 目标人群募集**

在项目县开展宣传工作，宣讲营养对孕妇、乳母和儿童的意义，宣传儿童辅助补充品“营养包”的营养素构成、对健康的意义和食用方法。村医登记申请领取营养包的儿童和家长姓名和联系方式。自愿参加营养调查的孕妇、乳母和儿童家长签署知情同意书。

**3.1.3 调查指标与数据采集：**

（1）体格检查：目标人群的身高/身长（YSC-2卧式身长测量器，北京国旺兴达体重秤有限公司；TZG身高座高器，江阴市第二医疗器械厂）、体重（TC100KA，北京华晟仪器有限责任公司；ACS-20-YE 电子婴儿秤，无锡市衡器厂有限公司）、腰围（非孕期妇女，火炬型腰围尺）、血压（上海医疗设备厂生产的玉兔牌台式血压计）。

（2）生物学监测：经家长同意给13月龄-36月龄儿童、妇女、乳母采集静脉血3-5毫升，现场测定全血血红蛋白（HemoCue 301），将全血静置30分钟后，离心分离血清置-30度保存，然后将冰冻状态血清样品空运至北京存于-80度，测定测量CRP（自动分析仪）、维生素A（HPLC法）、维生素D（放免法）、铁蛋白（放免法）、叶酸（放免法）、维生素B_12_（放免法）等的含量。0-12月龄儿童，从指尖末梢采血测定血红蛋白含量（Hemo Cue 301）。

（3）问卷调查：儿童基本情况调查内容包括儿童父母职业和文化程度、儿童出生时的基本情况、患病史、母乳喂养情况（按WHO的IYCF问卷和指标）、食物摄入情况（按WHO的IYCF问卷和评价指标）。孕妇和乳母情况调查表询问怀孕和乳母分娩时的情况、婴幼儿科学喂养知识掌握程度（按WHO的IYCF问卷和评价指标）、营养素补充剂使用情况等。

**3.2 营养干预**

**3.2.1 营养包干预**

对正安县、武定县和镇安县约15000名6－23月龄的婴幼儿提供辅食营养包干预，每个月儿童家长自愿到村医处领取营养包，并在村医的指导下给儿童食用营养包，促进婴幼儿生长发育，减少婴幼儿疾病发生，改善贫困地区婴幼儿营养状况。在干预期间内，推荐目标儿童每天服用1袋营养包，满6个月婴儿即为干预对象，满24月龄的儿童不再进行干预。项目干预持续18个月。

营养包发放周期及发放量如下：

企业

发放周期：2个月

县疾控

发放周期：2个月，一星期内发完

乡镇卫生院

发放周期：1个月，一星期内发完

医生

发放周期：1个月，一星期内发完

婴幼儿家长

**3.2.2营养包的采购与供应**

由联合国儿童基金会按其采购规定及项目要求进行营养包招标。确定供应商及营养包采购价格。按项目计划将营养包的需求量及供货要求通知供应商，并要求供应商按时向项目县提供营养包。营养包由联合国儿童基金会招标确定企业负责生产，企业按项目要求完成营养包生产。中国疾病预防控制中心营养与食品安全所（营养食品所）根据3个项目县实施单位上报婴幼儿数计算营养包数量，报送儿基会。儿基会根据营养食品所上报营养包数量向中标企业下达生产定单，中标企业接到儿基会的生产定单，在合同约定时限内按照已确定的营养包配方完成营养包的生产，并按要求将营养包产品按时运输到各项目县指定地点。

**3.2.3营养包的质量控制**

产品技术要求和质量由生产企业和联合国儿童基金会制定和负责。内部质量控制：生产商负责产品生产过程及产品出厂前的质量控制，按企业标准进行产品相关指标检验，保证产品质量。外部质量监测：由项目指定具有检验能力的部门进行产品的外部质量监测，外部质量控制将对企业样品、项目点采集样品进行检测。营养包质量监测采样计划要求见下表。

**表1 项目营养包质量监测采样计划**

| 采样地点 | 计划采样时间 | 采样量 | 采样人 |
| --- | --- | --- | --- |
| 生产企业 | 2010年10月 | 3批×2包/批×30袋/包 | 项目质量监测人员 |
| 项目点 | 武定、镇安、正安 | | |
| 1）县储存点 | 第1次：2010年10月  第2次：2011年4月 | 1批×1包/批×30袋/包 | 中国CDC |
| 2）村医处（每次每县选2点取样） | 第1次：2010年10月  第2次：2011年4月 | 1批×1包/批×30袋/包 | 中国CDC |
| 3）农户家（每次每县选2户取样） | 第1次：2010年10月  第2次：2011年4月 | 6袋（从有剩余的家庭） | 中国CDC |

**3.2.4营养包在项目点的储存条件要求**

营养包在到达项目县后，分发过程其储存应达到食品储存的基本要求：1）有专人并上锁保管；2）货物应在室温下，避光、阴凉、通风、干燥处进行保存； 3）不应与有毒有害物质及医疗药品混储；4）存放要求离墙隔地（建议离墙30cm以上，隔地15cm以上）；5）需要有防鼠防虫害设施。

**3.2.5 宣传教育的内容与要求**

为项目县印刷和发放了《营养包使用手册》、《婴幼儿喂养指导手册》、《中国孕期、哺乳期妇女和0-6岁儿童膳食指南》、《儿童膳食宝塔张贴画》、《孕期、哺乳期膳食宝塔张贴画》、《母乳喂养、合理辅食添加张贴画》等宣传材料。每个村卫生所、乡镇卫生院张贴张贴画，悬挂营养包宣传标语。每个县组织村医召开1-2次宣传会议，督促营养包分发和记录工作，给村医分发膳食指南书籍。给每位领取营养包的儿童家长分发和讲解营养包使用手册和婴幼儿喂养指导手册知识和使用方法。在开展营养包发放和指导服用的同时，由乡镇卫生院和村医采用面对面的宣传指导方式，指导儿童家长如何给孩子喂营养包，指导如何给孩子添加辅食。同时纠正不适当的喂养行为。同时，在县电视台播放宣传片“母爱的光辉”、 制作悬挂宣传横幅、举办健康教育宣传专栏。

**表2宣教材料和目标人群**

| 教材 | 宣教对象 |
| --- | --- |
| 专题讲座 | 乡镇卫生院工作人员和村医 |
| 入户指导和面对面宣传 | 儿童家长 |
| 营养包使用手册 | 儿童家长 |
| 婴幼儿喂养指导手册 | 儿童家长 |
| 中国孕期、哺乳期妇女和0-6岁儿童膳食指南 | 县、乡镇、村医 |
| 儿童膳食宝塔张贴画 | 乡镇卫生院和村卫生室 |
| 孕期、哺乳期膳食宝塔张贴画 | 乡镇卫生院和村卫生室 |
| 母乳喂养、合理辅食添加张贴画 | 乡镇卫生院和村卫生室 |
| 宣传标语 | 乡镇和村街道等显著位置 |
| 黑板报、营养专栏 | 乡镇医院和村卫生室 |
| DVD宣传片 | 县电视台 |

**3.3 终期营养调查（营养干预效果评价）**

在营养干预结束后，对各县0-3岁儿童营养状况开展调查，与基线调查结果比较，评价营养干预的效果。调查内容同基线营养状况调查，并调查营养包的服用情况。

**4 质量控制**

统一体检设备与采血耗材，调查员均经项目组统一进行培训，培训合格后在国家和省项目组联合的督导下进行现场调查。调查问卷采用双人双录入法核对以保证录入质量。生物样本的相同检测项目在一个实验室完成，采用统一的质控样品，保证检测的准确性。

**5 数据录入与统计分析**

采用EPIDATA进行数据录入，采用SAS9.2进行统计分析，计算均数与标准差或中位数与四分位数。计数资料以百分数表示，用WHO Anthro软件计算婴幼儿Z评分及体育发育状况评价。用线性模型和卡方检验分析差异显著性，Logistic回归分析危险因素及OR值与95%CI。P<0.05为差异有统计学意义。

**6. 项目时间表**

| 进度安排  任务 | 2010年 | | | | | | | | | | | | | 2011年 | | | | | | | | | | | | 2012年 | | | | | | | | | | | | |
| --- | --- | --- | --- | --- | --- | --- | --- | --- | --- | --- | --- | --- | --- | --- | --- | --- | --- | --- | --- | --- | --- | --- | --- | --- | --- | --- | --- | --- | --- | --- | --- | --- | --- | --- | --- | --- | --- | --- |
| 月份 | 1 | 2 | 3 | 4 | 5 | 6 | 7 | 8 | 9 | 10 | 11 | 12 | 1 | | 2 | 3 | 4 | 5 | 6 | 7 | 8 | 9 | 10 | 11 | 12 | | 1 | 2 | 3 | 4 | 5 | 6 | 7 | 8 | 9 | 10 | 11 | 12 |
| 方案设计、确定 |  |  |  |  |  |  |  |  |  |  |  |  |  | |  |  |  |  |  |  |  |  |  |  |  | |  |  |  |  |  |  |  |  |  |  |  |  |
| 项目省沟通、项目县确定 |  |  |  |  |  |  |  |  |  |  |  |  |  | |  |  |  |  |  |  |  |  |  |  |  | |  |  |  |  |  |  |  |  |  |  |  |  |
| 宣传材料设计制作 |  |  |  |  |  |  |  |  |  |  |  |  |  | |  |  |  |  |  |  |  |  |  |  |  | |  |  |  |  |  |  |  |  |  |  |  |  |
| 营养包招标 |  |  |  |  |  |  |  |  |  |  |  |  |  | |  |  |  |  |  |  |  |  |  |  |  | |  |  |  |  |  |  |  |  |  |  |  |  |
| 国家级项目启动会、培训会 |  |  |  |  |  |  |  |  |  |  |  |  |  | |  |  |  |  |  |  |  |  |  |  |  | |  |  |  |  |  |  |  |  |  |  |  |  |
| 省县启动会培训会 |  |  |  |  |  |  |  |  |  |  |  |  |  | |  |  |  |  |  |  |  |  |  |  |  | |  |  |  |  |  |  |  |  |  |  |  |  |
| 基线调查 |  |  |  |  |  |  |  |  |  |  |  |  |  | |  |  |  |  |  |  |  |  |  |  |  | |  |  |  |  |  |  |  |  |  |  |  |  |
| 营养包生产 |  |  |  |  |  |  |  |  |  |  |  |  |  | |  |  |  |  |  |  |  |  |  |  |  | |  |  |  |  |  |  |  |  |  |  |  |  |
| 营养包发放 |  |  |  |  |  |  |  |  |  |  |  |  |  | |  |  |  |  |  |  |  |  |  |  |  | |  |  |  |  |  |  |  |  |  |  |  |  |
| 干预效果评价 |  |  |  |  |  |  |  |  |  |  |  |  |  | |  |  |  |  |  |  |  |  |  |  |  | |  |  |  |  |  |  |  |  |  |  |  |  |
| 乡、村级培训，营养知识宣传教育 |  |  |  |  |  |  |  |  |  |  |  |  |  | |  |  |  |  |  |  |  |  |  |  |  | |  |  |  |  |  |  |  |  |  |  |  |  |
| 国家级中期评估 |  |  |  |  |  |  |  |  |  |  |  |  |  | |  |  |  |  |  |  |  |  |  |  |  | |  |  |  |  |  |  |  |  |  |  |  |  |
| 省级督导 |  |  |  |  |  |  |  |  |  |  |  |  |  | |  |  |  |  |  |  |  |  |  |  |  | |  |  |  |  |  |  |  |  |  |  |  |  |
| 数据分析报告撰写 |  |  |  |  |  |  |  |  |  |  |  |  |  | |  |  |  |  |  |  |  |  |  |  |  | |  |  |  |  |  |  |  |  |  |  |  |  |
| 与其他部门负责项目的汇总分析 |  |  |  |  |  |  |  |  |  |  |  |  |  | |  |  |  |  |  |  |  |  |  |  |  | |  |  |  |  |  |  |  |  |  |  |  |  |
| 项目总结会 |  |  |  |  |  |  |  |  |  |  |  |  |  | |  |  |  |  |  |  |  |  |  |  |  | |  |  |  |  |  |  |  |  |  |  |  |  |
