# Supplementary material for: Effectiveness of community-based complementary food supplement (Yingyangbao) distribution in children aged 6-23 months in poor areas in China
Source: PLoS One. 2017 Mar 20;12(3):e0174302. doi: 10.1371/journal.pone.0174302 (PMC5358851; doi:10.1371/journal.pone.0174302)
Supplement: S3 File — (DOCX) [file pone.0174302.s003.docx]

| sex | bl | bw | hb | Age | folic | B12 | ferr | crp | time | retinol | vd | sachet | diver | va | iron |
| --- | --- | --- | --- | --- | --- | --- | --- | --- | --- | --- | --- | --- | --- | --- | --- |
| 2 | 81.5 | 9.51 | 12 | 20.96 |  |  |  |  | 0 |  |  |  | 1 | 1 | 1 |
| 2 | 95.5 | 13.53 | 13.8 | 22.54 | 10.75 |  | 29.51 | 0.001 | 0 | 0.99 | 75.83 |  | 1 | 1 | 1 |
| 2 | 71.5 | 9.61 | 9.9 | 9.95 |  |  |  |  | 0 |  |  |  | 1 | 1 | 1 |
| 1 | 75.5 | 8.95 | 10.6 | 12.25 |  |  |  |  | 0 |  |  |  | 1 | 1 | 1 |
| 1 | 70 | 8.11 | 10.1 | 9.76 | 16.22 |  | 5.24 | 0.002 | 0 | 0.94 | 101.04 |  | 0 | 0 | 0 |
| 1 | 76 | 10.42 | 12.1 | 16.16 | 7.82 |  | 13.38 | 0.002 | 0 | 1.05 | 62.83 |  | 0 | 1 | 0 |
| 1 | 70 | 7.99 | 11 | 7 |  |  |  |  | 0 |  |  |  | 1 | 0 | 1 |
| 1 | 80.5 | 10.30 | 13.1 | 18.89 | 36.81 |  | 32.43 | 0.05 | 0 | 0.88 | 96.24 |  | 1 | 1 | 1 |
| 1 | 80 | 9.82 | 11.3 | 16.59 | 11.74 |  | 8.42 | 0.033 | 0 | 1.00 | 83.42 |  | 0 | 0 | 0 |
| 2 | 78 | 8.84 | 12.6 | 17.54 | 8.46 |  | 11.89 | 0.007 | 0 | 1.00 | 91.01 |  | 1 | 1 | 0 |
| 1 | 76.6 | 9.50 | 11 | 13.54 |  |  |  |  | 0 |  |  |  | 1 | 1 | 1 |
| 2 | 71.1 | 8.39 | 11.9 | 8.9 |  |  |  |  | 0 |  |  |  | 1 | 1 | 0 |
| 1 | 81 | 9.87 | 11.9 | 20.01 |  |  | 50.83 | 0.003 | 0 |  | 52.76 |  | 1 | 1 | 1 |
| 1 | 71 | 9.30 | 11.5 | 6.83 |  |  |  |  | 0 |  |  |  | 0 | 1 | 0 |
| 1 | 70.5 | 7.45 | 12.1 | 7.95 |  |  |  |  | 0 |  |  |  | 1 | 1 | 1 |
| 1 | 77 | 9.80 | 11.6 | 10.25 |  |  |  |  | 0 |  |  |  | 0 | 1 | 0 |
| 1 | 72.5 | 8.79 | 11.7 | 8.21 |  |  |  |  | 0 |  |  |  | 1 | 1 | 0 |
| 1 | 82 | 10.35 | 11 | 18.73 | 5.84 |  | 9.5 | 0.002 | 0 |  | 88.74 |  | 1 | 1 | 1 |
| 2 | 85.5 | 9.86 | 10 | 19.91 | 30.64 |  | 48.41 | 0.003 | 0 |  | 118.57 |  | 0 | 0 | 0 |
| 2 | 80 | 10.51 | 12.3 | 17.28 | 5.58 |  | 19.46 | 0.002 | 0 |  | 94.18 |  | 1 | 1 | 1 |
| 1 | 80 | 9.20 | 13.1 | 14.55 |  |  |  |  | 0 |  |  |  | 0 | 1 | 1 |
| 2 | 72 | 8.86 | 10.7 | 10.97 |  |  |  |  | 0 |  |  |  | 0 | 1 | 0 |
| 2 | 79.5 | 9.81 | 14 | 15.54 | 16.29 |  |  | 0.003 | 0 |  | 96.24 |  | 1 | 1 | 1 |
| 1 | 79.2 | 8.99 | 12.7 | 15.64 |  |  | 12.06 | 0.004 | 0 |  | 82.75 |  | 0 | 0 | 1 |
| 2 | 68 | 7.40 | 11.3 | 7.06 |  |  |  |  | 0 |  |  |  | 0 | 1 | 0 |
| 2 | 73 | 8.51 | 12.8 | 11.86 | 15.86 |  |  | 0.003 | 0 |  | 79.81 |  | 1 | 1 | 1 |
| 2 | 72 | 10.15 | 12 | 9.23 |  |  |  |  | 0 |  |  |  | 0 | 1 | 1 |
| 2 | 71 | 6.68 | 11.6 | 10.12 |  |  |  |  | 0 |  |  |  | 0 | 1 | 0 |
| 2 | 77 | 9.65 | 13.9 | 18.92 | 28.74 |  | 18.87 | 0.04 | 0 |  | 97.52 |  | 1 | 1 | 0 |
| 2 | 69 | 6.94 | 11.1 | 10.22 |  |  |  |  | 0 |  |  |  | 1 | 1 | 0 |
| 2 | 75.5 | 8.28 | 10.7 | 8.57 |  |  |  |  | 0 |  |  |  | 0 | 0 | 1 |
| 1 | 85.5 | 11.74 | 11.5 | 21.49 |  |  |  |  | 0 |  |  |  | 1 | 1 | 1 |
| 2 | 74.2 | 8.88 | 11.8 | 12.52 |  |  | 28.29 | 0.002 | 0 |  | 89.11 |  | 0 | 1 | 0 |
| 1 | 74.8 | 8.53 | 12.3 | 13.9 |  |  |  |  | 0 |  |  |  | 1 | 1 | 1 |
| 1 | 75 | 9.20 | 11.3 | 14.29 |  |  |  |  | 0 |  |  |  | 0 | 1 | 0 |
| 1 | 69 | 9.09 | 11.6 | 8.28 |  |  |  |  | 0 |  |  |  | 1 | 1 | 1 |
| 1 | 72 | 7.36 | 10.9 | 10.61 |  |  |  |  | 0 |  |  |  | 0 | 1 | 0 |
| 1 | 79.4 | 11.36 | 10.4 | 16 | 12.27 |  | 37.61 | 0.241 | 0 | 0.93 | 103.82 |  | 0 | 1 | 1 |
| 2 | 68 | 7.41 | 7.3 | 8.54 |  |  |  |  | 0 |  |  |  | 0 | 0 | 0 |
| 1 | 80 | 10.18 | 11.8 | 16.99 | 5.56 |  | 23.4 | 0.002 | 0 |  | 76.72 |  | 0 | 1 | 0 |
| 1 | 80 | 9.19 | 10.9 | 18.92 |  |  |  |  | 0 |  |  |  | 1 | 1 | 0 |
| 1 | 78 | 9.50 | 11.7 | 13.34 |  |  |  |  | 0 |  |  |  | 1 | 1 | 1 |
| 1 | 65 | 8.75 | 10.2 | 6.51 |  |  |  |  | 0 |  |  |  | 0 | 0 | 0 |
| 2 | 78 | 10.10 | 11.5 | 17.12 |  |  |  |  | 0 |  |  |  | 1 | 1 | 1 |
| 1 | 76 | 8.54 | 12.7 | 11.6 |  |  |  |  | 0 |  |  |  | 1 | 1 | 1 |
| 1 | 74 | 8.70 | 13.1 | 10.78 |  |  |  |  | 0 |  |  |  | 1 | 1 | 1 |
| 2 | 75 | 9.90 | 9.1 | 12.09 |  |  |  |  | 0 |  |  |  | 1 | 1 | 1 |
| 1 | 71 | 10.72 | 9.8 | 11.14 |  |  |  |  | 0 |  |  |  | 1 | 1 | 1 |
| 2 | 71 | 7.90 | 9.7 | 9.99 |  |  |  |  | 0 |  |  |  | 1 | 1 | 0 |
| 1 | 74.2 | 8.45 | 11.4 | 11.89 |  |  |  |  | 0 |  |  |  | 1 | 1 | 1 |
| 2 | 80 | 9.28 | 13.7 | 18.56 |  |  |  |  | 0 |  |  |  | 0 | 1 | 0 |
| 2 | 72 | 9.23 | 11 | 10.28 |  |  |  |  | 0 |  |  |  | 0 | 1 | 0 |
| 1 | 76 | 8.61 | 11 | 12.62 | 41.28 |  | 25.67 | 0.003 | 0 | 1.10 | 90.24 |  | 1 | 1 | 1 |
| 1 | 75 | 8.17 | 12.6 | 10.71 |  |  |  |  | 0 |  |  |  | 1 | 1 | 1 |
| 1 | 91 | 13.86 | 14.2 | 21.16 |  |  |  |  | 0 |  |  |  | 1 | 1 | 1 |
| 1 | 71 | 8.03 | 11.2 | 9.82 |  |  |  |  | 0 |  |  |  | 1 | 1 | 1 |
| 2 | 89 | 11.61 | 12.7 | 22.97 | 12.14 |  | 23.83 | 0.002 | 0 | 1.23 | 76.42 |  | 1 | 1 | 1 |
| 2 | 75.5 | 9.46 | 11.4 | 13.4 |  |  |  |  | 0 |  |  |  | 1 | 1 | 0 |
| 1 | 80.4 | 10.50 | 11.9 | 16.16 | 24.83 |  | 29.98 | 0.002 | 0 | 1.37 | 109.76 |  | 1 | 1 | 1 |
| 2 | 66.9 | 7.24 | 12.4 | 7.56 |  |  |  |  | 0 |  |  |  | 1 | 1 | 1 |
| 2 | 67 | 7.51 | 11.7 | 9.53 |  |  |  |  | 0 |  |  |  | 1 | 1 | 0 |
| 1 | 70.5 | 9.27 | 12.2 | 9.2 |  |  |  |  | 0 |  |  |  | 1 | 1 | 0 |
| 2 | 81.5 | 10.65 | 13 | 18.66 |  |  |  |  | 0 |  |  |  | 1 | 1 | 0 |
| 2 | 81 | 9.61 | 12.1 | 16.07 |  |  |  |  | 0 |  |  |  | 1 | 1 | 1 |
| 2 | 82 | 10.67 | 11.3 | 21.59 |  |  |  |  | 0 |  |  |  | 0 | 1 | 0 |
| 2 | 68 | 8.67 | 10.3 | 6.7 |  |  |  |  | 0 |  |  |  | 0 | 0 | 0 |
| 1 | 76 | 9.45 | 11.3 | 7 |  |  |  |  | 0 |  |  |  | 0 | 1 | 0 |
| 1 | 73.4 | 7.96 | 10.6 | 14.03 |  |  |  |  | 0 |  |  |  | 1 | 1 | 0 |
| 2 | 67 | 8.25 | 10.6 | 8.51 |  |  |  |  | 0 |  |  |  | 0 | 1 | 0 |
| 1 | 74.4 | 6.85 | 12.9 | 12.06 |  |  |  |  | 0 |  |  |  | 1 | 1 | 1 |
| 2 | 78.3 | 7.99 | 10.7 | 14.95 |  |  |  |  | 0 |  |  |  | 1 | 1 | 0 |
| 1 | 73 | 8.75 | 10.6 | 10.32 |  |  |  |  | 0 |  |  |  | 1 | 1 | 1 |
| 1 | 66.4 | 6.81 | 8.8 | 6.08 |  |  |  |  | 0 |  |  |  | 0 | 0 | 0 |
| 2 | 71.5 | 8.27 | 11.3 | 11.6 |  |  |  |  | 0 |  |  |  | 0 | 1 | 0 |
| 2 | 79 | 9.58 | 10.2 | 17.38 |  |  |  |  | 0 |  |  |  | 1 | 1 | 1 |
| 2 | 74 | 8.20 | 9.8 | 14.03 |  |  |  |  | 0 |  |  |  | 1 | 1 | 0 |
| 1 | 81 | 11.79 | 12.8 | 16.89 | 32.7 |  | 74.72 | 0.056 | 0 | 0.91 | 107.86 |  | 0 | 1 | 1 |
| 2 | 76 | 7.57 | 9.6 | 16.66 |  |  |  |  | 0 |  |  |  | 1 | 1 | 0 |
| 1 | 78 | 9.95 | 11.3 | 15.74 | 11.03 |  | 24.45 | 0.04 | 0 | 1.08 | 63.53 |  | 0 | 0 | 0 |
| 2 | 86 | 11.93 | 10.8 | 16.99 | 16.05 |  | 8.47 | 0.002 | 0 | 1.13 | 94.55 |  | 0 | 1 | 0 |
| 2 | 78.5 | 9.72 | 11.4 | 20.11 |  |  |  |  | 0 |  |  |  | 1 | 1 | 0 |
| 2 | 67.3 | 7.48 | 11.9 | 7.72 |  |  |  |  | 0 |  |  |  | 0 | 1 | 0 |
| 1 | 75 | 9.51 | 11.8 | 11.43 |  |  |  |  | 0 |  |  |  | 0 | 1 | 0 |
| 2 | 67 | 7.46 | 12 | 7.13 |  |  |  |  | 0 |  |  |  | 0 | 0 | 0 |
| 2 | 82 | 9.87 | 11.3 | 16.95 |  |  |  |  | 0 |  |  |  | 1 | 1 | 1 |
| 2 | 65.8 | 6.43 | 11.7 | 8.08 |  |  |  |  | 0 |  |  |  | 0 | 1 | 0 |
| 1 | 72.8 | 8.95 | 10.6 | 12.81 |  |  |  |  | 0 |  |  |  | 0 | 1 | 0 |
| 2 | 70.5 | 8.95 | 9.6 | 10.61 |  |  |  |  | 0 |  |  |  | 1 | 1 | 1 |
| 2 | 75 | 8.93 | 11.1 | 16.82 |  |  |  |  | 0 |  |  |  | 0 | 1 | 0 |
| 2 | 68 | 7.04 | 9.5 | 10.15 |  |  |  |  | 0 |  |  |  | 0 | 1 | 0 |
| 2 | 80 | 9.74 | 11.9 | 19.98 | 8.14 |  | 16.61 | 0.002 | 0 |  | 37.80 |  | 0 | 0 | 1 |
| 2 | 71.6 | 8.92 | 11.7 | 11.01 |  |  |  |  | 0 |  |  |  | 1 | 1 | 0 |
| 2 | 77 | 9.25 | 10.2 | 13.11 |  |  |  |  | 0 |  |  |  | 1 | 1 | 1 |
| 1 | 81.3 | 10.36 | 9.5 | 19.48 |  |  |  |  | 0 |  |  |  | 1 | 1 | 1 |
| 2 | 78 | 9.28 | 11.6 | 20.21 |  |  |  |  | 0 |  |  |  | 1 | 1 | 0 |
| 1 | 80 | 10.09 | 10.2 | 20.67 |  |  |  |  | 0 |  |  |  | 0 | 1 | 0 |
| 2 | 68 | 8.10 | 11.8 | 7 |  |  |  |  | 0 |  |  |  | 0 | 0 | 0 |
| 1 | 81 | 9.85 | 11.7 | 20.27 |  |  |  |  | 0 |  |  |  | 1 | 1 | 1 |
| 2 | 72.1 | 8.53 | 8.3 | 11.01 |  |  |  |  | 0 |  |  |  | 0 | 0 | 1 |
| 2 | 70 | 8.51 | 9.7 | 10.41 |  |  |  |  | 0 |  |  |  | 1 | 1 | 1 |
| 1 | 73 | 9.61 | 10.9 | 11.79 |  |  |  |  | 0 |  |  |  | 1 | 1 | 1 |
| 1 | 69 | 8.53 | 11.7 | 7.69 |  |  |  |  | 0 |  |  |  | 0 | 0 | 1 |
| 2 | 71.1 | 7.90 | 12.4 | 11.53 |  |  |  |  | 0 |  |  |  | 0 | 1 | 0 |
| 1 | 78 | 11.25 | 12.4 | 13.7 |  |  |  |  | 0 |  |  |  | 1 | 1 | 1 |
| 1 | 68 | 7.96 | 12.4 | 7.66 |  |  |  |  | 0 |  |  |  | 0 | 0 | 0 |
| 1 | 85 | 10.95 | 13.7 | 18.14 |  |  |  |  | 0 |  |  |  | 1 | 1 | 1 |
| 2 | 78 | 9.08 | 11.5 | 20.67 |  |  |  |  | 0 |  |  |  | 1 | 1 | 1 |
| 2 | 79 | 10.40 | 10.6 | 15.15 |  |  |  |  | 0 |  |  |  | 1 | 1 | 0 |
| 1 | 73.2 | 9.34 | 11 | 13.27 |  |  |  |  | 0 |  |  |  | 0 | 0 | 0 |
| 1 | 78.5 | 10.79 | 10.6 | 16.16 |  |  |  |  | 0 |  |  |  | 1 | 1 | 1 |
| 1 | 75 | 9.99 | 11.5 | 14.06 |  |  |  |  | 0 |  |  |  | 1 | 1 | 1 |
| 1 | 75 | 9.12 | 11 | 14.42 |  |  |  |  | 0 |  |  |  | 0 | 1 | 1 |
| 2 | 78 | 9.95 | 11.1 | 15.34 |  |  |  |  | 0 |  |  |  | 0 | 0 | 1 |
| 1 | 67 | 8.86 | 11.1 | 6.01 |  |  |  |  | 0 |  |  |  | 0 | 0 | 1 |
| 2 | 77.5 | 9.00 | 10.7 | 16.13 |  |  |  |  | 0 |  |  |  | 1 | 1 | 1 |
| 2 | 80 | 10.41 | 10.4 | 16.69 |  |  |  |  | 0 |  |  |  | 1 | 1 | 0 |
| 1 | 86 | 11.55 | 12.1 | 23.62 |  |  |  |  | 0 |  |  |  | 1 | 0 | 1 |
| 1 | 77 | 9.25 | 10.7 | 17.58 |  |  |  |  | 0 |  |  |  | 1 | 1 | 1 |
| 1 | 75 | 9.40 | 12.1 | 15.31 |  |  |  |  | 0 |  |  |  | 0 | 1 | 0 |
| 2 | 81 | 9.66 | 12.2 | 23.69 |  |  |  |  | 0 |  |  |  | 0 | 0 | 0 |
| 2 | 74 | 8.62 | 10.3 | 12.98 |  |  |  |  | 0 |  |  |  | 0 | 1 | 0 |
| 2 | 69 | 7.76 | 10.7 | 10.02 |  |  |  |  | 0 |  |  |  | 1 | 1 | 1 |
| 1 | 82 | 10.47 | 12.3 | 19.22 |  |  |  |  | 0 |  |  |  | 0 | 1 | 0 |
| 1 | 70 | 8.15 | 9.8 | 10.35 |  |  |  |  | 0 |  |  |  | 1 | 1 | 1 |
| 2 | 74 | 9.56 | 10 | 11.5 |  |  |  |  | 0 |  |  |  | 0 | 0 | 0 |
| 2 | 81 | 9.99 | 12.6 | 20.76 |  |  |  |  | 0 |  |  |  | 1 | 1 | 1 |
| 1 | 77 | 9.77 | 10.7 | 13.14 |  |  |  |  | 0 |  |  |  | 1 | 1 | 0 |
| 1 | 79 | 9.20 | 12.5 | 19.65 |  |  |  |  | 0 |  |  |  | 1 | 1 | 1 |
| 1 | 81 | 10.50 | 13.3 | 20.07 |  |  |  |  | 0 |  |  |  | 1 | 1 | 0 |
| 1 | 83 | 12.89 | 11.2 | 18.6 |  |  |  |  | 0 |  |  |  | 0 | 0 | 0 |
| 1 | 76 | 10.00 | 14 | 14.42 |  |  |  |  | 0 |  |  |  | 1 | 1 | 0 |
| 2 | 69 | 7.68 | 9.8 | 10.05 |  |  |  |  | 0 |  |  |  | 1 | 1 | 1 |
| 1 | 78 | 9.18 | 13.1 | 17.97 | 14.21 |  | 12.23 | 0.045 | 0 |  | 132.85 |  | 0 | 1 | 0 |
| 1 | 85 | 12.56 | 12.8 | 20.86 |  |  |  |  | 0 |  |  |  | 1 | 1 | 0 |
| 2 | 73 | 9.22 | 11.3 | 12.16 |  |  |  |  | 0 |  |  |  | 0 | 0 | 0 |
| 2 | 77 | 9.24 | 12 | 15.34 |  |  |  |  | 0 |  |  |  | 1 | 1 | 1 |
| 2 | 72 | 9.63 | 11.8 | 9 |  |  |  |  | 0 |  |  |  | 0 | 0 | 0 |
| 1 | 82 | 10.01 | 10.5 | 20.04 | 13.59 |  | 6.2 | 0.002 | 0 | 0.89 | 85.29 |  | 0 | 0 | 0 |
| 1 | 75 | 9.92 | 10 | 10.51 |  |  |  |  | 0 |  |  |  | 0 | 0 | 0 |
| 1 | 82 | 11.09 | 8.9 | 23.98 | 16.92 |  |  | 0.045 | 0 |  |  |  | 1 | 1 | 1 |
| 2 | 75.5 | 9.02 | 11.2 | 15.31 |  |  |  |  | 0 |  |  |  | 1 | 1 | 0 |
| 2 | 77 | 8.52 | 10.7 | 16.26 |  |  |  |  | 0 |  |  |  | 1 | 1 | 1 |
| 1 | 73.6 | 9.03 | 11 | 10.48 |  |  |  |  | 0 |  |  |  | 1 | 1 | 1 |
| 2 | 72.5 | 9.32 | 11.6 | 9.76 |  |  |  |  | 0 |  |  |  | 1 | 1 | 1 |
| 2 | 80 | 11.24 | 13.2 | 21.19 | 3.98 |  | 16.11 | 0.046 | 0 | 1.39 | 115.64 |  | 1 | 1 | 1 |
| 1 | 74 | 9.64 | 9 | 13.08 |  |  |  |  | 0 |  |  |  | 0 | 1 | 1 |
| 1 | 75 | 9.61 | 11.3 | 10.28 |  |  |  |  | 0 |  |  |  | 0 | 0 | 0 |
| 2 | 64 | 6.98 | 10.8 | 7.79 |  |  |  |  | 0 |  |  |  | 1 | 1 | 1 |
| 2 | 77 | 9.50 | 12 | 16.62 |  |  |  |  | 0 |  |  |  | 0 | 1 | 0 |
| 1 | 75 | 8.43 | 10.6 | 14.09 |  |  |  |  | 0 |  |  |  | 0 | 0 | 0 |
| 1 | 78 | 9.22 | 12.9 | 18.5 |  |  |  |  | 0 |  |  |  | 0 | 1 | 0 |
| 1 | 80 | 9.99 | 11.5 | 20.04 |  |  |  |  | 0 |  |  |  | 1 | 1 | 1 |
| 2 | 65 | 7.35 | 9.6 | 6.28 |  |  |  |  | 0 |  |  |  | 0 | 0 | 0 |
| 2 | 74 | 7.85 | 11.1 | 14.26 |  |  |  |  | 0 |  |  |  | 1 | 1 | 1 |
| 2 | 80.5 | 9.23 | 11 | 15.7 |  |  |  |  | 0 |  |  |  | 0 | 1 | 1 |
| 2 | 73 | 8.25 | 13 | 12.22 |  |  |  |  | 0 |  |  |  | 1 | 1 | 1 |
| 2 | 65 | 7.07 | 11.6 | 7.39 |  |  |  |  | 0 |  |  |  | 0 | 1 | 0 |
| 1 | 80 | 9.49 | 12.4 | 21.19 |  |  |  |  | 0 |  |  |  | 1 | 1 | 1 |
| 1 | 84 | 13.31 | 10.4 | 11.1 |  |  |  |  | 0 |  |  |  | 1 | 1 | 0 |
| 1 | 82 | 10.17 | 10.9 | 19.58 |  |  |  |  | 0 |  |  |  | 1 | 1 | 1 |
| 1 | 74 | 8.52 | 10.7 | 13.37 |  |  |  |  | 0 |  |  |  | 0 | 0 | 0 |
| 1 | 79.5 | 9.27 | 11.9 | 18.69 |  |  |  |  | 0 |  |  |  | 1 | 1 | 1 |
| 1 | 84 | 10.86 | 11.5 | 22.87 |  |  |  |  | 0 |  |  |  | 0 | 0 | 0 |
| 1 | 71 | 9.65 | 12.4 | 8.11 |  |  |  |  | 0 |  |  |  | 0 | 1 | 0 |
| 1 | 73.3 | 8.36 | 11.4 | 13.73 |  |  |  |  | 0 |  |  |  | 0 | 0 | 0 |
| 1 | 81.5 | 10.82 | 14.5 | 22.8 | 14.71 |  | 20.36 | 0.002 | 0 | 0.75 | 70.25 |  | 1 | 1 | 1 |
| 1 | 79 | 11.35 | 13 | 14.88 | 16.85 |  | 20.88 | 0.003 | 0 | 1.13 | 76.59 |  | 1 | 1 | 1 |
| 1 | 70.5 | 8.26 | 9.3 | 10.97 |  |  |  |  | 0 |  |  |  | 0 | 0 | 0 |
| 2 | 83.6 | 11.27 | 12.3 | 21.91 |  |  |  |  | 0 |  |  |  | 1 | 1 | 1 |
| 2 | 76 | 10.43 | 11.2 | 13.8 | 15.12 |  | 8.89 | 0.003 | 0 | 1.08 | 70.25 |  | 1 | 1 | 0 |
| 1 | 72.2 | 8.34 | 10.3 | 10.18 |  |  |  |  | 0 |  |  |  | 0 | 0 | 0 |
| 2 | 68 | 7.37 | 10.5 | 10.71 |  |  |  |  | 0 |  |  |  | 0 | 1 | 0 |
| 2 | 71 | 7.38 | 11 | 6.37 |  |  |  |  | 0 |  |  |  | 1 | 1 | 0 |
| 1 | 78.8 | 10.11 | 12.3 | 7.59 | 8.18 |  | 19.6 | 0.004 | 0 |  | 60.13 |  | 1 | 1 | 1 |
| 2 | 70 | 8.15 | 10.7 | 10.81 |  |  |  |  | 0 |  |  |  | 0 | 1 | 0 |
| 2 | 75.5 | 8.11 | 12 | 15.93 |  |  |  |  | 0 |  |  |  | 1 | 1 | 1 |
| 1 | 80 | 8.87 | 12.5 | 17.84 | 12.98 |  | 6.99 | 0.003 | 0 | 1.00 | 68.71 |  | 1 | 1 | 1 |
| 2 | 63 | 6.55 | 12.5 | 6.77 |  |  |  |  | 0 |  |  |  | 1 | 1 | 0 |
| 1 | 72 | 9.72 | 11.5 | 8.34 |  |  |  |  | 0 |  |  |  | 0 | 1 | 0 |
| 1 | 76 | 9.42 | 11.8 | 15.64 | 9.77 |  | 21.26 | 0.002 | 0 | 0.82 | 86.12 |  | 1 | 1 | 1 |
| 1 | 89.2 | 14.25 | 13.4 | 18.33 |  |  |  |  | 0 |  |  |  | 1 | 1 | 0 |
| 2 | 77.5 | 9.35 | 11.6 | 19.81 | 9.54 |  | 22.18 | 0.842 | 0 |  | 51.20 |  | 1 | 0 | 1 |
| 1 | 72.5 | 8.75 | 9.7 | 11.96 |  |  |  |  | 0 |  |  |  | 1 | 1 | 1 |
| 2 | 72.2 | 7.78 | 10.1 | 9.36 |  |  |  |  | 0 |  |  |  | 0 | 0 | 0 |
| 1 | 80.5 | 10.22 | 13.1 | 16.1 |  |  |  |  | 0 |  |  |  | 0 | 0 | 0 |
| 1 | 76.5 | 9.65 | 12.8 | 15.28 | 3.56 |  | 12.43 | 0.003 | 0 | 0.84 | 72.92 |  | 1 | 1 | 0 |
| 2 | 79 | 9.74 | 11.1 | 8.44 |  |  |  |  | 0 |  |  |  | 1 | 1 | 0 |
| 1 | 72 | 8.48 | 10.4 | 10.61 |  |  |  |  | 0 |  |  |  | 0 | 0 | 0 |
| 1 | 81.8 | 9.86 | 12.2 | 18.6 |  |  |  |  | 0 |  |  |  | 1 | 1 | 0 |
| 1 | 85 | 11.26 | 11.5 | 13.9 | 10.1 |  | 8.97 | 0.002 | 0 | 0.97 | 64.12 |  | 1 | 1 | 1 |
| 1 | 70 | 8.58 | 10.1 | 10.25 |  |  |  |  | 0 |  |  |  | 0 | 1 | 0 |
| 2 | 79 | 9.69 | 11.1 | 22.44 |  |  |  |  | 0 |  |  |  | 1 | 1 | 1 |
| 2 | 76 | 8.71 | 12.7 | 16.79 | 8.74 |  | 37.81 | 0.003 | 0 |  | 66.97 |  | 0 | 1 | 1 |
| 1 | 78 | 10.60 | 9.4 | 8.21 |  |  |  |  | 0 |  |  |  | 0 | 1 | 0 |
| 2 | 70.5 | 8.07 | 11.5 | 12.35 |  |  |  |  | 0 |  |  |  | 0 | 0 | 0 |
| 2 | 82 | 10.39 | 9.3 | 20.24 |  |  |  |  | 0 |  |  |  | 0 | 0 | 0 |
| 1 | 85 | 12.36 | 11.8 | 22.28 |  |  | 9.1 | 0.003 | 0 | 0.98 | 112.45 |  | 1 | 0 | 1 |
| 1 | 67 | 7.51 | 9.4 | 6.37 |  |  |  |  | 0 |  |  |  | 0 | 0 | 0 |
| 1 | 80 | 10.40 | 10.4 | 17.15 |  |  |  |  | 0 |  |  |  | 1 | 1 | 1 |
| 2 | 71 | 9.11 | 11.5 | 9.46 |  |  |  |  | 0 |  |  |  | 0 | 0 | 0 |
| 1 | 70 | 8.64 | 11.9 | 8.48 |  |  |  |  | 0 |  |  |  | 0 | 1 | 0 |
| 1 | 77 | 9.55 | 14 | 15.31 |  |  |  |  | 0 |  |  |  | 1 | 1 | 0 |
| 2 | 84 | 12.84 | 13.1 | 22.64 |  |  |  |  | 0 |  |  |  | 0 | 0 | 0 |
| 1 | 83 | 10.24 | 9.5 | 17.77 |  |  |  |  | 0 |  |  |  | 0 | 1 | 0 |
| 2 | 71.5 | 8.23 | 12 | 12.52 |  |  |  |  | 0 |  |  |  | 0 | 0 | 0 |
| 2 | 85 | 11.05 | 10.6 | 20.86 |  |  |  |  | 0 |  |  |  | 0 | 1 | 0 |
| 2 | 80 | 9.68 | 11.4 | 22.7 | 5.94 |  | 6.99 | 0.004 | 0 | 1.08 | 89.57 |  | 1 | 1 | 1 |
| 1 | 70 | 8.57 | 11.3 | 6.18 |  |  |  |  | 0 |  |  |  | 0 | 0 | 0 |
| 1 | 68 | 8.12 | 11.1 | 8.41 |  |  |  |  | 0 |  |  |  | 0 | 0 | 0 |
| 1 | 70 | 9.06 | 8.9 | 8.34 |  |  |  |  | 0 |  |  |  | 1 | 1 | 0 |
| 2 | 66 | 6.48 | 10.7 | 9.23 |  |  |  |  | 0 |  |  |  | 0 | 0 | 0 |
| 1 | 75 | 9.37 | 12.9 | 10.05 |  |  |  |  | 0 |  |  |  | 0 | 1 | 0 |
| 2 | 66 | 7.74 | 12.4 | 7.56 |  |  |  |  | 0 |  |  |  | 1 | 1 | 0 |
| 1 | 71 | 9.14 | 9.6 | 9.86 |  |  |  |  | 0 |  |  |  | 0 | 1 | 0 |
| 2 | 66 | 8.65 | 11.4 | 8.21 |  |  |  |  | 0 |  |  |  | 1 | 1 | 0 |
| 2 | 80 | 10.00 | 11.6 | 20.83 |  |  |  |  | 0 |  |  |  | 0 | 0 | 0 |
| 1 | 74.5 | 8.28 | 11.8 | 13.37 |  |  |  |  | 0 |  |  |  | 0 | 0 | 0 |
| 2 | 68 | 7.81 | 11.1 | 10.74 |  |  |  |  | 0 |  |  |  | 0 | 0 | 1 |
| 2 | 76 | 9.51 | 12.3 | 14.36 |  |  |  |  | 0 |  |  |  | 1 | 1 | 1 |
| 2 | 80 | 9.99 | 11.4 | 16.33 | 12.35 |  | 14.73 | 0.004 | 0 | 1.31 | 130.34 |  | 1 | 1 | 1 |
| 2 | 77.5 | 9.52 | 11.8 | 14.85 |  |  |  |  | 0 |  |  |  | 1 | 1 | 1 |
| 2 | 73 | 8.76 | 13.5 | 14.46 |  |  |  |  | 0 |  |  |  | 1 | 1 | 1 |
| 2 | 79 | 10.18 | 12.3 | 21.36 | 13.75 |  | 4.66 | 0.122 | 0 | 0.84 | 80.79 |  | 0 | 1 | 0 |
| 2 | 81 | 9.21 | 12.1 | 18.92 | 12.19 |  | 30.91 | 0.004 | 0 | 1.01 | 103.53 |  | 1 | 1 | 0 |
| 2 | 71 | 7.92 | 8.2 | 10.22 |  |  |  |  | 0 |  |  |  | 0 | 0 | 0 |
| 1 | 81 | 10.46 | 14.2 | 20.7 |  |  |  |  | 0 |  |  |  | 1 | 1 | 0 |
| 2 | 71 | 8.72 | 11.4 | 11.63 |  |  |  |  | 0 |  |  |  | 1 | 0 | 1 |
| 2 | 74 | 8.73 | 11.4 | 14.09 |  |  |  |  | 0 |  |  |  | 1 | 1 | 1 |
| 1 | 84 | 10.77 | 9.5 | 23.59 |  |  |  |  | 0 |  |  |  | 0 | 0 | 0 |
| 2 | 65 | 8.58 | 9.1 | 7.06 |  |  |  |  | 0 |  |  |  | 0 | 1 | 0 |
| 1 | 69 | 8.45 | 12.4 | 10.12 |  |  |  |  | 0 |  |  |  | 0 | 0 | 0 |
| 1 | 66 | 7.51 | 12.3 | 7.72 |  |  |  |  | 0 |  |  |  | 0 | 0 | 0 |
| 2 | 81.2 | 9.38 | 10.6 | 21.52 |  |  |  |  | 0 |  |  |  | 0 | 0 | 0 |
| 1 | 71 | 8.99 | 11.2 | 9.63 |  |  |  |  | 0 |  |  |  | 0 | 0 | 0 |
| 1 | 80 | 9.30 | 12.6 | 8.61 |  |  |  |  | 0 |  |  |  | 0 | 0 | 0 |
| 2 | 76 | 8.92 | 12.1 | 9.49 |  |  |  |  | 0 |  |  |  | 0 | 0 | 0 |
| 1 | 75 | 9.94 | 8.8 | 14.95 |  |  |  |  | 0 |  |  |  | 0 | 0 | 0 |
| 2 | 74 | 9.75 | 9.8 | 15.74 |  |  |  |  | 0 |  |  |  | 0 | 0 | 0 |
| 2 | 73 | 8.66 | 10.8 | 11.7 |  |  |  |  | 0 |  |  |  | 0 | 0 | 0 |
| 1 | 70 | 7.92 | 10.2 | 7.13 |  |  |  |  | 0 |  |  |  | 0 | 0 | 0 |
| 2 | 78 | 9.66 | 10.3 | 9.4 |  |  |  |  | 0 |  |  |  | 0 | 0 | 0 |
| 2 | 75 | 8.30 | 8.3 | 13.6 |  |  |  |  | 0 |  |  |  | 0 | 0 | 0 |
| 2 | 75 | 8.30 | 12.7 | 13.83 |  |  |  |  | 0 |  |  |  | 0 | 1 | 0 |
| 2 | 78 | 7.80 | 12.3 | 21.06 | 17.93 |  | 16.09 | 0.002 | 0 | 1.12 | 76.96 |  | 0 | 0 | 0 |
| 2 | 82 | 9.70 | 11.5 | 21.26 |  |  |  |  | 0 |  |  |  | 0 | 0 | 1 |
| 1 | 77 | 9.70 | 10.3 | 12.16 |  |  |  |  | 0 |  |  |  | 0 | 0 | 1 |
| 2 | 68.5 | 8.80 | 12.1 | 9.53 |  |  |  |  | 0 |  |  |  | 1 | 1 | 1 |
| 1 | 75 | 9.80 | 10.1 | 13.54 |  |  |  |  | 0 |  |  |  | 0 | 0 | 0 |
| 1 | 82.5 | 11.50 | 9.4 | 12.55 |  |  |  |  | 0 |  |  |  | 0 | 0 | 1 |
| 2 | 69 | 8.70 | 11.5 | 10.78 |  |  |  |  | 0 |  |  |  | 0 | 1 | 1 |
| 1 | 82 | 11.50 | 11.5 | 18.04 |  |  |  |  | 0 |  |  |  | 0 | 0 | 0 |
| 1 | 70 | 9.50 | 11.2 | 9.17 |  |  |  |  | 0 |  |  |  | 0 | 0 | 0 |
| 2 | 71 | 7.80 | 12.1 | 12.45 |  |  |  |  | 0 |  |  |  | 1 | 1 | 0 |
| 1 | 73 | 8.00 | 11 | 11.4 |  |  |  |  | 0 |  |  |  | 0 | 1 | 0 |
| 2 | 66 | 5.80 | 10.2 | 6.67 |  |  |  |  | 0 |  |  |  | 1 | 1 | 0 |
| 1 | 72 | 7.70 | 11.2 | 10.68 |  |  |  |  | 0 |  |  |  | 0 | 0 | 0 |
| 1 | 67 | 8.70 | 10.6 | 7.43 |  |  |  |  | 0 |  |  |  | 0 | 1 | 0 |
| 2 | 77.5 | 8.90 | 11.3 | 18.1 |  |  |  |  | 0 |  |  |  | 0 | 1 | 0 |
| 1 | 82 | 9.50 | 10.3 | 19.35 |  |  |  |  | 0 |  |  |  | 1 | 0 | 1 |
| 2 | 77 | 8.70 | 11.7 | 18.96 |  |  |  |  | 0 |  |  |  | 1 | 1 | 1 |
| 2 | 79 | 9.70 | 13.8 | 21.22 |  |  |  |  | 0 |  |  |  | 1 | 0 | 1 |
| 2 | 74 | 11.20 | 12 | 16.2 |  |  |  |  | 0 |  |  |  | 0 | 1 | 0 |
| 1 | 74 | 7.80 | 12.5 | 11.24 |  |  |  |  | 0 |  |  |  | 0 | 0 | 0 |
| 2 | 88 | 11.50 | 11.7 | 23.82 |  |  |  |  | 0 |  |  |  | 1 | 1 | 1 |
| 1 | 74 | 9.50 | 11.7 | 12.91 |  |  |  |  | 0 |  |  |  | 1 | 1 | 1 |
| 1 | 75 | 10.70 | 12.4 | 13.34 |  |  |  |  | 0 |  |  |  | 1 | 1 | 0 |
| 2 | 71.5 | 7.70 | 10.3 | 13.27 |  |  |  |  | 0 |  |  |  | 0 | 0 | 0 |
| 1 | 79 | 8.50 | 14 | 15.24 |  |  |  |  | 0 |  |  |  | 0 | 1 | 0 |
| 1 | 74 | 8.80 | 11.3 | 14.92 |  |  |  |  | 0 |  |  |  | 1 | 1 | 1 |
| 1 | 75 | 8.30 | 11.4 | 14.92 |  |  |  |  | 0 |  |  |  | 1 | 1 | 1 |
| 2 | 75 | 9.70 | 10.8 | 16.49 |  |  |  |  | 0 |  |  |  | 0 | 0 | 0 |
| 1 | 85 | 12.68 | 12.5 | 23.1 |  |  |  |  | 0 |  |  |  | 0 | 1 | 0 |
| 2 | 76 | 8.70 | 12.3 | 16.49 |  |  |  |  | 0 |  |  |  | 0 | 0 | 0 |
| 1 | 76 | 9.70 | 12.3 | 11.96 |  |  |  |  | 0 |  |  |  | 0 | 0 | 0 |
| 1 | 79.3 | 9.25 | 13 | 20.21 |  |  |  |  | 0 |  |  |  | 0 | 0 | 0 |
| 2 | 75.5 | 9.70 | 12.3 | 14.69 |  |  |  |  | 0 |  |  |  | 0 | 0 | 0 |
| 1 | 65 | 8.70 | 11.9 | 6.57 |  |  |  |  | 0 |  |  |  | 0 | 0 | 0 |
| 1 | 72 | 7.30 | 11.3 | 12.25 |  |  |  |  | 0 |  |  |  | 0 | 0 | 0 |
| 1 | 90 | 12.70 | 11.2 | 21.75 |  |  |  |  | 0 |  |  |  | 0 | 0 | 0 |
| 1 | 81 | 10.80 | 11.3 | 14.75 |  |  |  |  | 0 |  |  |  | 0 | 0 | 0 |
| 2 | 75.5 | 7.80 | 11.7 | 18.04 |  |  |  |  | 0 |  |  |  | 0 | 0 | 0 |
| 1 | 79 | 9.75 | 9.8 | 20.21 |  |  |  |  | 0 |  |  |  | 0 | 0 | 0 |
| 2 | 68 | 8.70 | 9.8 | 10.02 |  |  |  |  | 0 |  |  |  | 0 | 0 | 0 |
| 1 | 78 | 9.50 | 11.5 | 16.39 |  |  |  |  | 0 |  |  |  | 0 | 0 | 0 |
| 1 | 70.5 | 9.50 | 10.9 | 9.59 |  |  |  |  | 0 |  |  |  | 0 | 0 | 0 |
| 2 | 84 | 10.60 | 12.6 | 21.85 |  |  | 7.51 | 0.196 | 0 | 0.93 | 113.81 |  | 1 | 1 | 1 |
| 1 | 79.6 | 9.32 | 13.4 | 17.91 |  |  | 16.66 | 0.025 | 0 | 0.92 | 117.39 |  | 1 | 1 | 1 |
| 2 | 78.2 | 7.67 | 13 | 16 | 17.13 | 534.93 | 24.39 | 0.002 | 0 | 0.89 | 136.93 |  | 1 | 1 | 1 |
| 1 | 72.2 | 7.00 | 12.3 | 10.74 |  |  |  |  | 0 |  |  |  | 1 | 1 | 1 |
| 2 | 76.5 | 8.62 | 12.6 | 14.82 | 21.09 | 223.8 | 12.13 | 0.024 | 0 |  | 73.94 |  | 1 | 1 | 1 |
| 1 | 75.6 | 9.61 | 12.3 | 14.92 | 20.12 | 627.21 | 42.77 | 0.005 | 0 | 1.01 | 120.67 |  | 1 | 1 | 0 |
| 2 | 78.9 | 10.17 | 12.7 | 18.6 | 24.83 | 424.18 | 28.39 | 0.384 | 0 |  | 64.06 |  | 1 | 1 | 1 |
| 1 | 81.5 | 10.58 | 13.2 | 17.91 | 32.02 | 277.74 | 37 | 0.058 | 0 | 0.86 | 97.12 |  | 0 | 1 | 0 |
| 2 | 68.5 | 6.68 | 12.7 | 9.33 |  |  |  |  | 0 |  |  |  | 0 | 1 | 0 |
| 1 | 78.5 | 10.15 | 10.9 | 14.49 |  |  | 4.12 | 0.025 | 0 | 0.90 | 53.35 |  | 1 | 1 | 1 |
| 1 | 86.3 | 10.03 | 12.7 | 18.46 |  |  | 13.26 | 0.011 | 0 | 1.32 | 137.93 |  | 1 | 1 | 1 |
| 1 | 72.3 | 9.51 | 10.3 | 10.87 |  |  |  |  | 0 |  |  |  | 1 | 1 | 1 |
| 2 | 89.3 | 12.45 | 13.1 | 22.57 | 10.86 | 301.32 | 16.86 | 0.002 | 0 | 1.16 | 96.75 |  | 0 | 1 | 1 |
| 1 | 68.1 | 7.23 | 12.1 | 7.29 |  |  |  |  | 0 |  |  |  | 0 | 0 | 0 |
| 1 | 68.5 | 9.05 | 14 | 9.07 |  |  |  |  | 0 |  |  |  | 1 | 0 | 1 |
| 2 | 71.1 | 8.14 | 11.9 | 14.06 | 37.28 | 346.39 | 8.7 | 0.003 | 0 | 0.96 | 64.86 |  | 1 | 1 | 1 |
| 2 | 75.9 | 9.75 | 11.7 | 13.01 |  |  |  |  | 0 |  |  |  | 0 | 0 | 1 |
| 2 | 73.1 | 9.75 | 13.5 | 13.11 | 22.11 | 122.58 | 7.5 | 0.004 | 0 | 0.88 | 91.57 |  | 0 | 0 | 0 |
| 2 | 70.1 | 7.59 | 12.3 | 9.4 |  |  |  |  | 0 |  |  |  | 0 | 0 | 1 |
| 1 | 74.1 | 9.20 | 11.6 | 14.03 |  |  | 34.66 | 0.036 | 0 | 0.75 | 92.13 |  | 0 | 0 | 1 |
| 2 | 73 | 7.36 | 12.8 | 14.88 | 6.87 | 237.46 | 4.96 | 0.002 | 0 | 1.05 | 75.56 |  | 1 | 1 | 0 |
| 2 | 80.5 | 11.70 | 13.6 | 21.85 | 10.72 | 258.36 | 14.47 | 0.093 | 0 | 1.01 | 82.76 |  | 1 | 1 | 1 |
| 2 | 70 | 9.50 | 10.7 | 7.2 |  |  | 24.69 | 0.002 | 0 |  | 63.78 |  | 0 | 0 | 0 |
| 1 | 77.7 | 9.40 | 11.5 | 15.41 |  |  | 2.8 | 0.03 | 0 |  | 98.56 |  | 0 | 0 | 0 |
| 1 | 70.3 | 7.37 | 10.6 | 7.49 |  |  |  |  | 0 |  |  |  | 0 | 1 | 0 |
| 1 | 82.4 | 9.93 | 11.9 | 21.16 | 24.05 | 370.31 | 40.06 | 0.003 | 0 | 1.31 | 44.13 |  | 1 | 1 | 0 |
| 1 | 83.5 | 9.35 | 13.6 | 18.37 |  |  | 28.21 | 0.03 | 0 | 1.20 | 58.09 |  | 0 | 1 | 0 |
| 1 | 76.3 | 9.45 | 11.1 | 12.58 |  | 625.86 | 7.94 | 0.04 | 0 | 1.04 | 92.13 |  | 0 | 1 | 0 |
| 1 | 70.6 | 9.80 | 11.8 | 9.4 |  |  |  |  | 0 |  |  |  | 1 | 1 | 0 |
| 1 | 82.8 | 10.86 | 12.5 | 21.03 | 9.16 | 328.77 | 17.12 | 0.002 | 0 | 1.07 | 93.82 |  | 1 | 1 | 0 |
| 1 | 71.5 | 8.31 | 11.6 | 11.66 |  |  |  |  | 0 |  |  |  | 0 | 0 | 0 |
| 1 | 82.1 | 10.85 | 12.1 | 20.21 | 6.75 | 255.79 | 7.59 | 0.002 | 0 | 1.16 | 118.99 |  | 1 | 1 | 0 |
| 2 | 76.5 | 8.96 | 12 | 13.9 | 7.17 | 460.78 | 5.86 | 0.002 | 0 | 0.88 | 83.29 |  | 1 | 1 | 1 |
| 2 | 78.2 | 9.50 | 11.7 | 15.93 | 6.61 | 460.26 | 24.38 | 0.03 | 0 | 1.17 | 57.20 |  | 1 | 0 | 1 |
| 1 | 75.6 | 7.63 | 12.4 | 15.97 | 9.96 | 91.357 | 15.31 | 0.064 | 0 | 0.95 | 76.88 |  | 1 | 0 | 1 |
| 2 | 77.5 | 8.85 | 12.7 | 18.5 | 32.69 | 587.28 | 18.28 | 0.002 | 0 | 1.08 | 118.18 |  | 1 | 1 | 1 |
| 1 | 76.1 | 10.10 | 11.5 | 11.01 |  |  |  |  | 0 |  |  |  | 0 | 1 | 0 |
| 2 | 81.1 | 8.96 | 12.2 | 21.55 |  |  | 11.92 | 0.003 | 0 | 1.08 | 103.00 |  | 0 | 0 | 1 |
| 1 | 73.5 | 8.69 | 11.9 | 8.31 |  |  |  |  | 0 |  |  |  | 1 | 1 | 1 |
| 1 | 75.9 | 10.13 | 12.1 | 13.5 | 16.02 | 196.36 | 14.28 | 0.002 | 0 | 0.91 | 131.83 |  | 1 | 1 | 1 |
| 1 | 83.5 | 12.49 | 12.8 | 22.05 | 7.78 | 158.05 | 23.92 | 0.002 | 0 |  | 67.08 |  | 1 | 1 | 1 |
| 2 | 68.1 | 7.71 | 12.9 | 8.57 |  |  |  |  | 0 |  |  |  | 0 | 0 | 0 |
| 1 | 80.1 | 9.38 | 12.8 | 21.42 |  |  | 7.78 | 0.103 | 0 | 0.93 | 81.42 |  | 1 | 1 | 1 |
| 1 | 70.2 | 8.26 | 10.3 | 8.08 |  |  |  |  | 0 |  |  |  | 1 | 1 | 1 |
| 2 | 76.1 | 8.26 | 12.1 | 12.62 |  |  |  |  | 0 |  |  |  | 1 | 1 | 1 |
| 2 | 77.1 | 8.03 | 11.4 | 17.61 | 12.75 | 251.79 | 22.6 | 0.03 | 0 | 1.14 | 125.90 |  | 0 | 1 | 1 |
| 1 | 80.2 | 9.65 | 13.8 | 17.38 | 7.13 | 245.3 | 22.09 | 0.002 | 0 | 0.93 | 131.83 |  | 1 | 1 | 1 |
| 2 | 89 | 8.23 | 13.5 | 23.29 | 9.66 | 300.53 | 16.83 | 0.003 | 0 | 1.11 | 97.94 |  | 1 | 1 | 0 |
| 2 | 71.1 | 7.48 | 13.7 | 8.34 |  |  |  |  | 0 |  |  |  | 0 | 1 | 0 |
| 2 | 77.2 | 8.96 | 12.9 | 18.27 | 12.98 | 318.12 | 28.35 | 0.003 | 0 | 1.16 | 100.84 |  | 1 | 1 | 1 |
| 2 | 73 | 8.40 | 13 | 11.17 |  |  |  |  | 0 |  |  |  | 0 | 1 | 0 |
| 1 | 75.2 | 10.09 | 10.2 | 12.62 |  |  |  |  | 0 |  |  |  | 1 | 1 | 1 |
| 1 | 64.5 | 7.51 | 10.9 | 6.54 |  |  |  |  | 0 |  |  |  | 0 | 0 | 0 |
| 2 | 65 | 6.07 | 12 | 7.2 |  |  |  |  | 0 |  |  |  | 0 | 1 | 0 |
| 2 | 68.9 | 6.99 | 13 | 7.62 |  |  |  |  | 0 |  |  |  | 1 | 1 | 1 |
| 2 | 78.5 | 9.61 | 11.2 | 17.41 |  |  |  |  | 0 |  |  |  | 1 | 1 | 1 |
| 1 | 82.8 | 10.14 | 12.9 | 15.8 |  |  | 11.66 | 0.002 | 0 | 0.75 | 62.18 |  | 0 | 0 | 1 |
| 2 | 80.5 | 11.12 | 13.7 | 18.73 | 10.22 | 359.3 | 21.37 | 0.002 | 0 | 1.04 | 79.84 |  | 1 | 1 | 1 |
| 1 | 69 | 8.13 | 12.4 | 7.2 |  |  |  |  | 0 |  |  |  | 0 | 1 | 0 |
| 2 | 70.5 | 9.00 | 11.8 | 9.1 |  |  |  |  | 0 |  |  |  | 0 | 0 | 1 |
| 1 | 68.2 | 8.18 | 11.9 | 7.75 |  |  |  |  | 0 |  |  |  | 0 | 0 | 0 |
| 2 | 90.6 | 10.07 | 12.2 | 23.26 | 5 | 144.45 | 16.72 | 0.003 | 0 | 0.75 | 61.87 |  | 1 | 1 | 1 |
| 2 | 83 | 10.42 | 10.9 | 18.17 | 6.93 | 757.5 | 4.89 | 0.003 | 0 | 1.03 | 99.68 |  | 1 | 0 | 1 |
| 1 | 85.6 | 12.80 | 13.3 | 22.31 | 11.22 | 363.07 | 14.64 | 0.002 | 0 | 0.84 | 77.71 |  | 1 | 1 | 1 |
| 2 | 66.5 | 7.65 | 10.6 | 6.97 |  |  |  |  | 0 |  |  |  | 0 | 0 | 0 |
| 1 | 82.3 | 11.03 | 12 | 19.12 | 13.92 | 133.11 | 13.05 | 0.045 | 0 | 0.97 | 69.91 |  | 1 | 1 | 0 |
| 2 | 63 | 6.62 | 10.5 | 6.7 |  |  |  |  | 0 |  |  |  | 0 | 1 | 0 |
| 2 | 69.5 | 8.22 | 10.7 | 7.95 |  |  |  |  | 0 |  |  |  | 0 | 0 | 0 |
| 2 | 64.8 | 6.11 | 10.3 | 6.31 |  |  |  |  | 0 |  |  |  | 0 | 1 | 0 |
| 1 | 78 | 9.77 | 13.3 | 17.45 | 8.56 | 413.58 | 38.24 | 0.02 | 0 | 0.79 | 70.12 |  | 1 | 1 | 1 |
| 1 | 79.2 | 9.17 | 12.7 | 16.43 | 11.13 | 288.83 | 4.79 | 0.002 | 0 | 1.09 | 88.63 |  | 1 | 1 | 1 |
| 2 | 74.3 | 8.01 | 12.5 | 16.2 | 12.06 | 562.38 | 9.43 | 0.003 | 0 | 1.03 | 119.89 |  | 0 | 1 | 0 |
| 1 | 80.9 | 10.65 | 13.5 | 20.17 | 7.74 | 253.97 | 15.19 | 0.4 | 0 | 0.89 | 103.55 |  | 1 | 1 | 1 |
| 2 | 81 | 11.40 | 13 | 19.84 | 5.27 | 209.42 | 32.33 | 0.04 | 0 | 1.25 | 90.20 |  | 0 | 1 | 1 |
| 2 | 82.1 | 10.88 | 12.4 | 19.35 | 14.41 | 324.65 | 7.03 | 0.003 | 0 | 1.35 | 108.17 |  | 0 | 1 | 0 |
| 2 | 81.2 | 10.50 | 13.5 | 19.88 | 8.57 | 524.91 | 16.01 | 0.002 | 0 | 1.14 | 73.28 |  | 1 | 1 | 1 |
| 2 | 76 | 10.60 | 12.9 | 19.09 | 8.64 | 218.92 | 11.16 | 0.003 | 0 | 0.82 | 94.52 |  | 1 | 1 | 0 |
| 2 | 73 | 8.01 | 13.2 | 6.51 |  |  |  |  | 0 |  |  |  | 0 | 1 | 1 |
| 2 | 73 | 10.39 | 11.4 | 7.49 |  |  |  |  | 0 |  |  |  | 1 | 1 | 1 |
| 1 | 69 | 7.40 | 11.7 | 8.9 |  |  |  |  | 0 |  |  |  | 1 | 1 | 1 |
| 2 | 72 | 9.00 | 11.6 | 14.39 | 20.27 | 86.994 | 6.27 | 0.046 | 0 | 1.07 | 101.66 |  | 0 | 0 | 0 |
| 2 | 80 | 10.60 | 12.2 | 19.94 | 9.29 | 460.27 | 55.43 | 0.007 | 0 | 0.93 | 90.73 |  | 0 | 0 | 0 |
| 2 | 67 | 7.10 | 10.4 | 10.22 |  |  |  |  | 0 |  |  |  | 1 | 1 | 0 |
| 1 | 64 | 6.55 | 11.5 | 6.08 |  |  |  |  | 0 |  |  |  | 0 | 0 | 0 |
| 1 | 84 | 12.20 | 12.8 | 22.93 | 8.5 | 222.39 | 18.55 | 0.002 | 0 | 0.85 | 125.47 |  | 1 | 1 | 1 |
| 2 | 74.5 | 9.48 | 11.3 | 8.67 |  |  |  |  | 0 |  |  |  | 0 | 1 | 1 |
| 2 | 70.5 | 8.48 | 11.7 | 11.24 |  |  |  |  | 0 |  |  |  | 0 | 0 | 1 |
| 2 | 66 | 7.67 | 12.8 | 7.29 |  |  |  |  | 0 |  |  |  | 0 | 0 | 0 |
| 2 | 78.5 | 10.60 | 12.6 | 20.11 | 11.92 | 451.22 | 25.24 | 0.003 | 0 | 1.07 | 78.55 |  | 1 | 1 | 1 |
| 2 | 75.2 | 8.93 | 11 | 15.57 | 5.32 | 174.96 | 7.92 | 0.004 | 0 | 1.11 | 103.97 |  | 1 | 1 | 1 |
| 2 | 63.5 | 7.42 | 11.7 | 6.44 |  |  |  |  | 0 |  |  |  | 0 | 1 | 1 |
| 1 | 75.5 | 8.50 | 9.7 | 15.01 | 14.55 | 190.81 | 6.12 | 0.003 | 0 | 1.32 | 82.05 |  | 1 | 1 | 0 |
| 1 | 70.5 | 9.00 | 11 | 9.26 |  |  |  |  | 0 |  |  |  | 0 | 1 | 0 |
| 2 | 77.3 | 9.68 | 12.4 | 19.35 |  |  | 8.8 | 0.025 | 0 |  | 73.67 |  | 1 | 0 | 1 |
| 1 | 79.5 | 11.50 | 11.4 | 12.52 | 5.83 | 373.6 | 4.59 | 0.002 | 0 | 1.48 | 63.76 |  | 1 | 1 | 1 |
| 2 | 65 | 6.16 | 12.4 | 7.39 |  |  |  |  | 0 |  |  |  | 0 | 0 | 0 |
| 1 | 83.3 | 10.88 | 14.4 | 22.8 | 13.79 | 408.12 | 13.87 | 0.002 | 0 | 0.85 | 108.06 |  | 1 | 0 | 1 |
| 1 | 84.5 | 11.46 | 13.8 | 23.66 | 11.5 | 198.32 | 31.53 | 0.002 | 0 | 1.02 | 102.08 |  | 1 | 1 | 1 |
| 2 | 68.8 | 7.17 | 12.4 | 7.52 |  |  |  |  | 0 |  |  |  | 0 | 1 | 0 |
| 1 | 77.5 | 12.30 | 14.8 | 13.44 | 13.9 | 143.01 | 32.92 | 0.045 | 0 |  | 101.51 |  | 1 | 1 | 0 |
| 2 | 80.5 | 11.10 | 12.9 | 17.54 | 5.86 | 175.58 | 6.88 | 0.121 | 0 |  | 77.42 |  | 0 | 0 | 0 |
| 2 | 81.2 | 11.05 | 13.1 | 20.3 | 7.71 | 205.3 | 28.89 | 0.012 | 0 | 0.91 | 84.92 |  | 1 | 1 | 1 |
| 2 | 80 | 9.47 | 11.3 | 20.6 | 10.56 | 56.211 | 8.45 | 0.025 | 0 | 0.99 | 114.57 |  | 0 | 1 | 1 |
| 1 | 82 | 9.80 | 13.9 | 18.2 |  |  | 13.95 |  | 0 | 0.78 | 94.47 |  | 0 | 0 | 1 |
| 1 | 77.2 | 10.65 | 12.4 | 15.21 | 18.04 | 227.31 | 13.18 | 0.196 | 0 | 0.84 | 78.21 |  | 1 | 1 | 0 |
| 2 | 70.9 | 8.00 | 12.2 | 8.08 |  |  |  |  | 0 |  |  |  | 0 | 0 | 0 |
| 1 | 90.5 | 11.48 | 12.9 | 23.85 | 15.75 | 154.94 | 39.79 | 0.026 | 0 | 0.86 | 69.75 |  | 0 | 1 | 0 |
| 1 | 69 | 8.47 | 11.3 | 6.51 |  |  |  |  | 0 |  |  |  | 0 | 0 | 1 |
| 1 | 82.5 | 11.35 | 14 | 23.56 | 13.45 | 243.49 | 14.62 | 0.026 | 0 | 1.25 | 113.22 |  | 1 | 0 | 1 |
| 1 | 80.5 | 11.98 | 13.9 | 20.4 | 8.02 | 261.45 | 12.47 | 0.002 | 0 | 1.08 | 78.21 |  | 1 | 0 | 1 |
| 1 | 72 | 8.58 | 12.9 | 11.56 |  |  |  |  | 0 |  |  |  | 0 | 1 | 0 |
| 2 | 75.2 | 8.50 | 11.9 | 13.37 | 9.99 | 439.57 | 8.52 | 0.158 | 0 | 0.89 |  |  | 0 | 1 | 0 |
| 2 | 70.5 | 8.10 | 12.9 | 7.92 |  |  |  |  | 0 |  |  |  | 1 | 1 | 1 |
| 2 | 71.5 | 8.67 | 10.6 | 10.25 |  |  |  |  | 0 |  |  |  | 0 | 1 | 0 |
| 1 | 72 | 7.84 | 11 | 10.81 |  |  |  |  | 0 |  |  |  | 1 | 1 | 1 |
| 1 | 76 | 9.76 | 10.8 | 12.71 | 5.09 |  | 9.56 | 0.002 | 0 | 0.91 | 103.82 |  | 1 | 1 | 1 |
| 1 | 76 | 9.00 | 11.3 | 13.63 | 15.85 | 158.21 | 19.76 | 0.004 | 0 | 0.94 | 111.25 |  | 1 | 1 | 1 |
| 1 | 80 | 10.95 | 13.4 | 17.91 | 12.67 | 254.25 | 7.77 | 0.002 | 0 | 1.07 | 84.92 |  | 0 | 0 | 0 |
| 2 | 79.9 | 7.15 | 11.9 | 21.98 |  |  | 28.93 | 0.04 | 0 | 1.45 | 111.90 |  | 0 | 0 | 0 |
| 2 | 68.5 | 7.70 | 13.3 | 9 |  |  |  |  | 0 |  |  |  | 0 | 0 | 0 |
| 2 | 73 | 8.83 | 13.2 | 14.46 |  |  | 6.64 | 0.002 | 0 | 1.00 | 79.01 |  | 0 | 0 | 1 |
| 2 | 76 | 9.05 | 13 | 18.73 |  |  | 28.78 | 0.002 | 0 | 1.11 | 82.74 |  | 1 | 1 | 1 |
| 1 | 70.5 | 7.76 | 11.4 | 10.84 |  |  |  |  | 0 |  |  |  | 0 | 0 | 1 |
| 1 | 67 | 7.00 | 12.2 | 8.18 |  |  |  |  | 0 |  |  |  | 0 | 0 | 1 |
| 2 | 78 | 10.00 | 13.7 | 13.27 | 12.66 | 217.19 | 12.64 | 0.002 | 0 | 1.36 | 78.61 |  | 1 | 1 | 1 |
| 1 | 78 | 10.10 | 12.3 | 17.38 |  |  | 21.24 | 0.031 | 0 | 0.89 | 89.52 |  | 0 | 0 | 0 |
| 2 | 69.9 | 7.80 | 13.4 | 9.43 |  |  |  |  | 0 |  |  |  | 0 | 0 | 1 |
| 1 | 82 | 11.33 | 12.4 | 16.26 | 6.95 | 220.65 | 6 | 0.046 | 0 | 1.03 | 79.82 |  | 1 | 1 | 0 |
| 2 | 68 | 7.70 | 11.8 | 9.13 |  |  |  |  | 0 |  |  |  | 0 | 0 | 1 |
| 2 | 69.5 | 6.90 | 12.6 | 11.2 | 19.08 | 369.86 | 16.47 | 0.232 | 0 | 0.99 | 85.05 |  | 1 | 1 | 1 |
| 2 | 75 | 9.30 | 10.9 | 13.4 | 17.68 | 178.38 | 12.1 | 0.003 | 0 | 0.82 | 36.47 |  | 0 | 1 | 0 |
| 2 | 69.5 | 8.80 | 12.3 | 7.59 |  |  |  |  | 0 |  |  |  | 0 | 1 | 0 |
| 2 | 70.5 | 7.90 | 13.5 | 10.71 |  |  |  |  | 0 |  |  |  | 0 | 0 | 0 |
| 1 | 73.5 | 8.00 | 11.9 | 11.17 |  |  |  |  | 0 |  |  |  | 1 | 1 | 1 |
| 1 | 76 | 10.00 | 11.3 | 13.34 | 8.8 | 187.47 | 6.98 | 0.04 | 0 | 1.04 | 93.00 |  | 1 | 1 | 0 |
| 2 | 70 | 8.00 | 12.9 | 8.54 |  |  |  |  | 0 |  |  |  | 0 | 1 | 0 |
| 2 | 66.5 | 7.70 | 13.3 | 6.21 |  |  |  |  | 0 |  |  |  | 0 | 1 | 1 |
| 1 | 82.5 | 11.20 | 11.4 | 20.17 | 13.37 | 310.06 | 4.91 | 0.04 | 0 | 1.20 | 84.59 |  | 0 | 0 | 1 |
| 1 | 83.5 | 11.10 | 12.1 | 20.4 | 6.41 | 464.41 | 24.93 | 0.04 | 0 | 0.94 | 93.54 |  | 0 | 1 | 0 |
| 2 | 78 | 8.30 | 12.7 | 14.46 | 11.31 | 736.42 | 11.13 | 0.002 | 0 | 1.23 | 118.85 |  | 0 | 1 | 1 |
| 2 | 78.5 | 9.90 | 11.9 | 17.05 | 16.63 | 261.66 | 11.27 | 0.349 | 0 | 0.88 | 108.32 |  | 0 | 1 | 0 |
| 2 | 77 | 10.20 | 11.7 | 16.76 | 10.33 | 267.22 | 15.48 | 0.003 | 0 | 1.04 | 102.64 |  | 0 | 1 | 0 |
| 2 | 70 | 8.20 | 11.8 | 7.98 |  |  |  |  | 0 |  |  |  | 1 | 1 | 1 |
| 1 | 71.5 | 7.80 | 10.2 | 11.43 |  |  |  |  | 0 |  |  |  | 0 | 1 | 0 |
| 1 | 73.5 | 8.90 | 12.1 | 10.12 |  |  |  |  | 0 |  |  |  | 1 | 1 | 1 |
| 2 | 71.5 | 7.90 | 11.6 | 12.16 |  |  |  |  | 0 |  |  |  | 1 | 1 | 0 |
| 1 | 79.9 | 10.00 | 10.1 | 20.6 |  |  | 4.2 | 0.004 | 0 | 0.89 | 87.11 |  | 0 | 0 | 1 |
| 1 | 76.5 | 8.00 | 11.8 | 15.41 | 14.46 | 218.28 | 13.12 | 0.003 | 0 | 0.99 | 58.38 |  | 1 | 1 | 1 |
| 2 | 73.5 | 8.30 | 11.7 | 14.36 |  |  | 12.92 | 0.054 | 0 | 0.80 | 96.27 |  | 0 | 0 | 1 |
| 1 | 66.5 | 8.05 | 12.3 | 6.01 |  |  |  |  | 0 |  |  |  | 1 | 1 | 1 |
| 2 | 72.5 | 7.10 | 11.9 | 15.21 | 5.06 | 368.53 | 11.46 | 0.004 | 0 | 1.01 | 99.82 |  | 1 | 1 | 0 |
| 2 | 69.7 | 6.80 | 11.8 | 11.73 | 11.01 | 191.41 | 12.18 | 0.432 | 0 | 0.91 | 101.04 |  | 1 | 1 | 1 |
| 1 | 72 | 7.85 | 10.2 | 10.97 |  |  |  |  | 0 |  |  |  | 1 | 1 | 1 |
| 2 | 73 | 7.82 | 11.9 | 12.45 | 39.82 | 171 | 40.32 | 0.002 | 0 | 0.83 | 146.39 |  | 0 | 1 | 0 |
| 1 | 75.5 | 9.70 | 14.4 | 12.22 | 15.78 | 716.03 | 27.1 | 0.004 | 0 | 1.08 | 96.27 |  | 1 | 1 | 1 |
| 1 | 84.5 | 11.79 | 11.8 | 21.13 | 14.64 | 297.18 | 11.75 | 0.003 | 0 | 1.05 | 135.34 |  | 1 | 0 | 1 |
| 2 | 70.5 | 6.70 | 12.3 | 12.55 | 17.2 | 124.07 | 7.18 | 0.427 | 0 | 0.90 | 146.39 |  | 0 | 0 | 0 |
| 2 | 67.5 | 7.80 | 11.5 | 7.39 |  |  |  |  | 0 |  |  |  | 0 | 1 | 0 |
| 1 | 74.5 | 8.10 | 10.6 | 14.46 | 8.25 | 451.04 | 13.49 | 0.04 | 0 | 1.07 | 88.63 |  | 0 | 1 | 0 |
| 1 | 82.5 | 10.90 | 13 | 21.45 | 10.45 | 450.02 | 10.46 | 0.018 | 0 | 1.02 | 86.11 |  | 1 | 1 | 1 |
| 2 | 68.5 | 7.52 | 13.5 | 10.74 |  |  |  |  | 0 |  |  |  | 0 | 1 | 1 |
| 2 | 82 | 9.00 | 13.3 | 21.09 | 9.57 | 170.05 | 8.11 | 0.002 | 0 | 1.14 | 80.12 |  | 1 | 1 | 1 |
| 1 | 83 | 12.55 | 12.4 | 21.13 |  |  | 7.56 | 0.04 | 0 |  | 41.47 |  | 1 | 1 | 1 |
| 2 | 75 | 8.10 | 11.8 | 14.19 | 10.31 | 264.62 | 6.19 | 0.05 | 0 | 1.15 | 51.21 |  | 1 | 1 | 1 |
| 1 | 71 | 8.08 | 12.1 | 6.7 |  |  |  |  | 0 |  |  |  | 0 | 0 | 0 |
| 1 | 78.2 | 9.25 | 11.8 | 18.46 | 9.11 | 434.65 | 7.18 | 0.002 | 0 | 1.01 | 67.08 |  | 1 | 0 | 1 |
| 1 | 72 | 8.77 | 12.5 | 7.39 |  |  |  |  | 0 |  |  |  | 0 | 1 | 1 |
| 1 | 86 | 10.73 | 12.6 | 21.91 |  |  |  |  | 0 | 1.14 |  |  | 0 | 0 | 0 |
| 2 | 71 | 9.23 | 11 | 11.14 |  |  |  |  | 0 |  |  |  | 0 | 0 | 0 |
| 1 | 74 | 7.90 | 12.8 | 14.29 | 19.73 | 128.95 | 18.19 | 0.003 | 0 | 0.95 | 82.51 |  | 0 | 1 | 1 |
| 2 | 64 | 7.00 | 11.8 | 6.14 |  |  |  |  | 0 |  |  |  | 0 | 1 | 0 |
| 1 | 83 | 12.00 | 13 | 15.57 |  | 434.1 | 17.11 | 0.002 | 0 | 1.35 |  |  | 1 | 1 | 1 |
| 1 | 72 | 8.93 | 10.9 | 7.29 |  |  |  |  | 0 |  |  |  | 1 | 1 | 0 |
| 1 | 76.5 | 9.00 | 11.3 | 15.15 | 14.17 | 185.99 | 10.38 | 0.003 | 0 | 1.05 | 79.41 |  | 1 | 1 | 1 |
| 2 | 69 | 7.87 | 12.4 | 11.86 |  |  |  |  | 0 |  |  |  | 0 | 1 | 1 |
| 1 | 71.5 | 8.50 | 12.4 | 11.6 |  |  |  |  | 0 |  |  |  | 0 | 1 | 0 |
| 1 | 76.5 | 9.25 | 12.2 | 15.05 |  |  | 6.34 | 0.002 | 0 | 0.86 |  |  | 0 | 1 | 1 |
| 1 | 74.3 | 9.75 | 7.7 | 9.95 |  |  |  |  | 0 |  |  |  | 0 | 0 | 0 |
| 2 | 67 | 7.98 | 12.5 | 7.85 |  |  |  |  | 0 |  |  |  | 0 | 0 | 1 |
| 2 | 86.5 | 11.00 | 12.7 | 22.28 |  |  | 16.81 | 0.002 | 0 | 1.03 |  |  | 0 | 1 | 1 |
| 2 | 70 | 7.25 | 11.5 | 7.43 |  |  |  |  | 0 |  |  |  | 0 | 1 | 0 |
| 2 | 84.4 | 11.28 | 13.6 | 21.55 |  |  |  | 0.003 | 0 |  |  |  | 0 | 1 | 1 |
| 2 | 82.3 | 10.83 | 11.6 | 20.3 |  |  | 15.81 | 0.003 | 0 | 1.06 |  |  | 0 | 1 | 0 |
| 2 | 83 | 11.21 | 14.4 | 16.46 |  |  |  | 0.003 | 0 | 1.20 |  |  | 0 | 0 | 0 |
| 1 | 92 | 11.92 | 12.8 | 18.6 |  |  |  |  | 0 |  |  |  | 0 | 0 | 0 |
| 1 | 77.5 | 10.57 | 12.4 | 15.31 |  |  | 5.8 | 0.003 | 0 | 0.78 |  |  | 1 | 1 | 1 |
| 2 | 83.2 | 11.74 | 13.5 | 16.79 |  |  | 9.58 | 0.002 | 0 | 1.17 |  |  | 0 | 1 | 0 |
| 2 | 71 | 9.43 | 10.2 | 10.51 |  |  |  |  | 0 |  |  |  | 0 | 1 | 0 |
| 1 | 79.7 | 10.96 | 10.1 | 12.71 |  |  |  |  | 0 |  |  |  | 0 | 0 | 0 |
| 1 | 64.5 | 8.57 | 10.9 | 6.05 |  |  |  |  | 0 |  |  |  | 0 | 0 | 0 |
| 2 | 75.5 | 9.25 | 12 | 14.13 |  |  |  |  | 0 |  |  |  | 0 | 1 | 1 |
| 2 | 69 | 7.34 | 9.5 | 9.23 |  |  |  |  | 0 |  |  |  | 0 | 1 | 0 |
| 2 | 82.3 | 11.66 | 11.9 | 19.98 |  |  |  |  | 0 |  |  |  | 1 | 1 | 0 |
| 2 | 66.5 | 7.00 | 11.7 | 9.66 |  |  |  |  | 0 |  |  |  | 0 | 1 | 0 |
| 1 | 72.6 | 8.97 | 10.4 | 11.01 |  |  |  |  | 0 |  |  |  | 0 | 1 | 0 |
| 1 | 65.1 | 8.44 | 12.1 | 6.05 |  |  |  |  | 0 |  |  |  | 0 | 0 | 0 |
| 2 | 70 | 9.76 | 12.2 | 8.25 |  |  |  |  | 0 |  |  |  | 0 | 0 | 0 |
| 1 | 76.1 | 9.43 | 11.9 | 11.4 |  |  |  |  | 0 |  |  |  | 1 | 0 | 0 |
| 1 | 83.1 | 11.91 | 12.5 | 19.32 |  |  | 11.65 | 0.002 | 0 |  |  |  | 1 | 1 | 0 |
| 2 | 78 | 10.09 | 10.4 | 14 |  |  |  | 0.002 | 0 | 1.01 |  |  | 1 | 0 | 1 |
| 1 | 79.1 | 9.66 | 12.7 | 18.96 |  |  | 22 | 0.179 | 0 | 1.21 |  |  | 1 | 1 | 1 |
| 2 | 72.6 | 9.72 | 11.9 | 11.53 |  |  |  |  | 0 |  |  |  | 1 | 1 | 1 |
| 1 | 83.1 | 11.39 | 12.5 | 17.81 |  |  | 14.89 | 0.526 | 0 | 0.95 |  |  | 1 | 1 | 1 |
| 1 | 79.5 | 9.89 | 12.6 | 15.11 |  |  | 13.38 | 0.002 | 0 | 1.15 |  |  | 1 | 1 | 1 |
| 2 | 79.8 | 10.02 | 12.5 | 15.01 |  |  | 3.21 | 0.004 | 0 | 1.03 |  |  | 0 | 1 | 0 |
| 1 | 68.5 | 7.75 | 9.8 | 7.33 |  |  |  |  | 0 |  |  |  | 0 | 0 | 0 |
| 1 | 68.1 | 10.19 | 6 | 8.38 |  |  |  |  | 0 |  |  |  | 0 | 0 | 0 |
| 1 | 72.1 | 9.33 | 8.2 | 7.82 |  |  |  |  | 0 |  |  |  | 1 | 1 | 1 |
| 2 | 69.1 | 7.58 | 11.6 | 9.13 |  |  |  |  | 0 |  |  |  | 1 | 1 | 1 |
| 2 | 70 | 7.56 | 10.7 | 9.13 |  |  |  |  | 0 |  |  |  | 1 | 1 | 1 |
| 1 | 72.6 | 10.10 | 12.7 | 9.26 |  |  |  |  | 0 |  |  |  | 0 | 1 | 0 |
| 1 | 72 | 8.51 | 10.7 | 12.06 |  |  | 23.9 | 0.002 | 0 |  |  |  | 1 | 1 | 1 |
| 2 | 72.2 | 8.44 | 10.7 | 8.11 |  |  |  |  | 0 |  |  |  | 1 | 1 | 1 |
| 1 | 80.1 | 9.81 | 13.7 | 14.52 |  |  | 42.68 | 0.003 | 0 |  |  |  | 1 | 1 | 0 |
| 1 | 74.1 | 9.76 | 11.6 | 13.9 |  |  | 14.89 | 0.003 | 0 | 1.00 |  |  | 0 | 1 | 0 |
| 1 | 72.1 | 9.77 | 10.6 | 8.8 |  |  |  |  | 0 |  |  |  | 0 | 1 | 0 |
| 2 | 87.5 | 12.90 | 11 | 21.03 |  |  | 25.01 | 0.004 | 0 | 1.06 |  |  | 1 | 1 | 0 |
| 1 | 76.5 | 9.49 | 10.1 | 11.53 |  |  |  |  | 0 |  |  |  | 0 | 0 | 1 |
| 1 | 79.8 | 10.78 | 12.9 | 18.37 |  |  | 18.8 | 0.004 | 0 | 1.20 |  |  | 1 | 1 | 1 |
| 1 | 74 | 9.38 | 12.3 | 7.69 |  |  |  |  | 0 |  |  |  | 0 | 1 | 0 |
| 1 | 78.8 | 10.57 | 11 | 15.08 |  |  |  |  | 0 |  |  |  | 1 | 1 | 1 |
| 2 | 69.9 | 9.59 | 7.7 | 7.52 |  |  |  |  | 0 |  |  |  | 0 | 0 | 0 |
| 2 | 86.2 | 11.96 | 13.5 | 21.88 |  |  | 12.93 | 0.002 | 0 | 1.05 |  |  | 1 | 1 | 0 |
| 1 | 84.4 | 11.80 | 13.2 | 22.24 |  |  | 12.12 | 0.003 | 0 | 1.24 |  |  | 1 | 1 | 1 |
| 2 | 83 | 12.26 | 12.8 | 20.44 |  |  | 39.46 | 0.002 | 0 | 0.84 |  |  | 1 | 1 | 1 |
| 1 | 83 | 11.59 | 12.5 | 20.63 |  |  | 15.2 | 0.004 | 0 | 1.15 |  |  | 0 | 1 | 1 |
| 2 | 79.6 | 8.81 | 12.7 | 13.96 |  |  |  |  | 0 |  |  |  | 1 | 1 | 1 |
| 1 | 74.2 | 11.03 | 11.1 | 7.13 |  |  |  |  | 0 |  |  |  | 0 | 0 | 0 |
| 2 | 90.2 | 13.99 | 12.3 | 22.97 |  |  |  | 0.002 | 0 | 1.47 |  |  | 1 | 1 | 0 |
| 1 | 74.7 | 10.37 | 11.4 | 12.52 |  |  |  |  | 0 |  |  |  | 0 | 1 | 0 |
| 2 | 70.1 | 6.91 | 13.5 | 10.35 |  |  |  |  | 0 |  |  |  | 0 | 1 | 0 |
| 1 | 67.5 | 8.79 | 10.9 | 6.51 |  |  |  |  | 0 |  |  |  | 0 | 1 | 0 |
| 2 | 81 | 10.27 | 12 | 20.3 |  |  | 21.98 | 0.003 | 0 | 1.01 |  |  | 1 | 1 | 0 |
| 1 | 71 | 8.33 | 11.2 | 8.18 |  |  |  |  | 0 |  |  |  | 0 | 1 | 0 |
| 2 | 65.5 | 7.32 | 12.1 | 8.8 |  |  |  |  | 0 |  |  |  | 0 | 1 | 0 |
| 1 | 74 | 11.27 | 10.3 | 7.92 |  |  |  |  | 0 |  |  |  | 0 | 0 | 0 |
| 2 | 72.6 | 7.97 | 8.7 | 12.19 |  |  |  |  | 0 |  |  |  | 0 | 0 | 0 |
| 1 | 84.1 | 10.67 | 12.7 | 15.05 |  |  | 33.76 | 0.005 | 0 | 1.10 |  |  | 1 | 1 | 0 |
| 1 | 84.2 | 11.74 | 12.8 | 20.7 |  |  | 35.35 | 0.046 | 0 | 1.21 |  |  | 1 | 1 | 1 |
| 1 | 85.1 | 11.62 | 12 | 20.04 |  |  | 25.69 | 0.055 | 0 | 1.50 |  |  | 1 | 1 | 1 |
| 1 | 72.1 | 8.95 | 9.3 | 7.79 |  |  |  |  | 0 |  |  |  | 0 | 0 | 0 |
| 2 | 80.1 | 9.20 | 11.8 | 17.31 |  |  | 7.62 | 0.004 | 0 | 1.09 |  |  | 1 | 1 | 1 |
| 1 | 87.1 | 12.77 | 11.6 | 20.44 |  |  | 8.07 | 0.003 | 0 | 1.15 |  |  | 1 | 1 | 1 |
| 2 | 75.9 | 10.19 | 11.7 | 14.03 |  |  |  | 0.003 | 0 | 1.18 |  |  | 1 | 1 | 0 |
| 1 | 91.1 | 12.36 | 11.4 | 15.74 |  |  |  | 0.003 | 0 | 1.06 |  |  | 0 | 1 | 0 |
| 2 | 83 | 9.92 | 12.8 | 17.71 |  |  | 8.96 | 0.003 | 0 | 1.20 |  |  | 1 | 1 | 1 |
| 1 | 82.8 | 12.27 | 12.1 | 18.73 |  |  |  | 0.002 | 0 | 1.04 |  |  | 1 | 1 | 1 |
| 2 | 66.7 | 7.53 | 10.8 | 6.05 |  |  |  |  | 0 |  |  |  | 0 | 0 | 0 |
| 2 | 79 | 9.80 | 12.6 | 21.45 |  |  | 61.82 | 0.004 | 0 | 1.15 |  |  | 1 | 1 | 0 |
| 1 | 83 | 10.64 | 12.3 | 22.31 |  |  |  |  | 0 |  |  |  | 1 | 1 | 0 |
| 1 | 73.7 | 10.06 | 12.4 | 9.26 |  |  |  |  | 0 |  |  |  | 0 | 0 | 0 |
| 1 | 64.3 | 5.96 | 11.7 | 6.28 |  |  |  |  | 0 |  |  |  | 0 | 0 | 0 |
| 1 | 74.4 | 10.66 | 12.3 | 11.3 |  |  |  |  | 0 |  |  |  | 0 | 0 | 0 |
| 2 | 68.3 | 6.55 | 12.7 | 6.08 |  |  |  |  | 0 |  |  |  | 0 | 0 | 0 |
| 2 | 77.1 | 8.45 | 13.1 | 15.67 |  |  | 12.41 | 0.002 | 0 | 1.00 |  |  | 0 | 1 | 0 |
| 2 | 77.2 | 8.48 | 12.4 | 15.67 |  |  | 8.23 | 0.003 | 0 | 1.15 |  |  | 0 | 1 | 0 |
| 2 | 70.2 | 9.08 | 11.3 | 10.71 |  |  |  |  | 0 |  |  |  | 0 | 0 | 0 |
| 1 | 70 | 9.02 | 10.8 | 6.31 |  |  |  |  | 0 |  |  |  | 0 | 0 | 0 |
| 1 | 85.1 | 11.66 | 12.6 | 21.91 |  |  | 14.89 | 0.002 | 0 | 1.11 |  |  | 1 | 1 | 1 |
| 2 | 81.1 | 10.68 | 12.8 | 18.73 |  |  |  | 0.002 | 0 | 1.51 |  |  | 1 | 1 | 0 |
| 2 | 78.8 | 9.05 | 14.7 | 16.36 |  |  |  |  | 0 |  |  |  | 0 | 1 | 0 |
| 2 | 82.7 | 10.65 | 12.1 | 20.9 |  |  | 29.17 | 0.002 | 0 | 1.10 |  |  | 1 | 1 | 1 |
| 1 | 69 | 7.83 | 11.6 | 7.43 |  |  |  |  | 0 |  |  |  | 0 | 1 | 0 |
| 1 | 75.5 | 10.76 | 12.5 | 10.94 |  |  |  |  | 0 |  |  |  | 0 | 1 | 0 |
| 1 | 76.5 | 11.06 | 11.6 | 13.08 |  |  |  |  | 0 |  |  |  | 1 | 1 | 0 |
| 2 | 78.8 | 9.36 | 11.9 | 19.35 |  |  | 25.87 | 0.002 | 0 | 1.14 |  |  | 0 | 1 | 0 |
| 2 | 68.2 | 8.61 | 11.6 | 9.66 |  |  |  |  | 0 |  |  |  | 0 | 1 | 0 |
| 2 | 73.6 | 10.69 | 12.5 | 14.13 |  |  |  |  | 0 |  |  |  | 1 | 1 | 0 |
| 1 | 84.2 | 10.45 | 11.8 | 20.86 |  |  | 5.26 | 0.002 | 0 | 1.08 |  |  | 0 | 1 | 1 |
| 2 | 77.3 | 10.33 | 13 | 13.11 |  |  |  |  | 0 |  |  |  | 0 | 1 | 0 |
| 2 | 75.1 | 9.15 | 11.9 | 15.54 |  |  |  | 0.004 | 0 | 0.95 |  |  | 0 | 1 | 0 |
| 1 | 70.2 | 8.04 | 10.7 | 8.94 |  |  |  |  | 0 |  |  |  | 0 | 1 | 0 |
| 2 | 77.3 | 9.93 | 10.8 | 18.33 |  |  | 17.4 | 0.003 | 0 | 0.87 |  |  | 0 | 1 | 1 |
| 1 | 91 | 13.66 | 13.6 | 23.98 |  |  | 7.44 | 0.42 | 0 | 0.97 |  |  | 1 | 1 | 1 |
| 1 | 82 | 10.06 | 10.1 | 17.68 |  |  |  |  | 0 |  |  |  | 0 | 0 | 0 |
| 1 | 77.5 | 9.11 | 11.4 | 13.67 |  |  |  |  | 0 |  |  |  | 0 | 1 | 0 |
| 2 | 81.6 | 11.76 | 12.7 | 18.46 |  |  | 12.15 | 0.003 | 0 | 0.91 |  |  | 0 | 1 | 0 |
| 2 | 67.3 | 7.49 | 10.1 | 8.25 |  |  |  |  | 0 |  |  |  | 0 | 0 | 0 |
| 2 | 66.5 | 9.20 | 12.2 | 7.2 |  |  |  |  | 0 |  |  |  | 1 | 1 | 0 |
| 1 | 74.3 | 8.80 | 10.5 | 9.63 |  |  |  |  | 0 |  |  |  | 0 | 1 | 0 |
| 2 | 66.1 | 6.65 | 12 | 6.18 |  |  |  |  | 0 |  |  |  | 0 | 0 | 0 |
| 1 | 86.5 | 11.00 | 12 | 19.55 |  |  | 20.97 | 0.002 | 0 | 0.97 |  |  | 1 | 1 | 1 |
| 2 | 67.6 | 7.23 | 11.7 | 9.49 |  |  |  |  | 0 |  |  |  | 0 | 0 | 0 |
| 2 | 77.3 | 10.95 | 11.4 | 12.42 |  |  |  |  | 0 |  |  |  | 0 | 0 | 0 |
| 2 | 75 | 9.01 | 11.1 | 17.18 |  |  |  |  | 0 |  |  |  | 0 | 1 | 0 |
| 2 | 75.5 | 9.01 | 11.2 | 17.18 |  |  |  |  | 0 |  |  |  | 0 | 1 | 0 |
| 2 | 69.9 | 7.70 | 10 | 8.64 |  |  |  |  | 0 |  |  |  | 0 | 0 | 0 |
| 2 | 82.3 | 10.73 | 11.6 | 19.94 |  |  | 13.59 | 0.078 | 0 | 0.92 |  |  | 1 | 1 | 0 |
| 2 | 82 | 12.10 | 12.3 | 20.76 |  |  |  |  | 0 |  |  |  | 1 | 1 | 0 |
| 1 | 67.1 | 7.40 | 11 | 6.93 |  |  |  |  | 0 |  |  |  | 0 | 1 | 0 |
| 2 | 84.9 | 13.59 | 12.6 | 23.46 |  |  | 50.83 | 0.004 | 0 | 1.29 |  |  | 0 | 1 | 0 |
| 1 | 82.3 | 11.71 | 13.2 | 17.02 |  |  | 20.39 |  | 0 |  |  |  | 1 | 1 | 0 |
| 2 | 76.2 | 9.81 | 11.4 | 13.04 |  |  |  |  | 0 |  |  |  | 1 | 1 | 0 |
| 2 | 68.6 | 8.43 | 12.9 | 9.3 |  |  |  |  | 0 |  |  |  | 0 | 0 | 0 |
| 1 | 73.1 | 8.90 | 12.5 | 11.99 |  |  |  |  | 0 |  |  |  | 0 | 0 | 0 |
| 2 | 76.1 | 7.67 | 10.4 | 9.63 |  |  |  | 0.004 | 0 | 1.12 |  |  | 0 | 0 | 0 |
| 2 | 63.8 | 6.79 | 12.4 | 6.6 |  |  |  |  | 0 |  |  |  | 0 | 1 | 0 |
| 2 | 77.8 | 9.62 | 12.8 | 17.22 |  |  | 46.3 | 0.003 | 0 | 1.12 |  |  | 1 | 1 | 0 |
| 2 | 79.6 | 9.55 | 13 | 21.68 |  |  |  |  | 0 |  |  |  | 0 | 1 | 0 |
| 1 | 78.4 | 11.70 | 14.3 | 15.84 |  |  |  |  | 0 |  |  |  | 1 | 1 | 0 |
| 2 | 89.8 | 9.55 | 11.6 | 16.23 |  |  | 13.83 | 0.004 | 0 | 1.13 |  |  | 0 | 0 | 0 |
| 2 | 84.3 | 11.10 | 12.2 | 22.8 |  |  |  |  | 0 |  |  |  | 1 | 1 | 0 |
| 2 | 83.7 | 10.29 | 13 | 21.68 |  |  | 12.09 | 0.002 | 0 | 0.97 |  |  | 0 | 0 | 1 |
| 2 | 81.5 | 12.87 | 11 | 18.33 |  |  | 10.21 | 0.005 | 0 | 0.96 |  |  | 1 | 1 | 1 |
| 1 | 82.7 | 9.14 | 10.8 | 23.23 |  |  | 25.4 |  | 0 |  |  |  | 0 | 1 | 0 |
| 1 | 81.2 | 11.88 | 12.5 | 9.17 |  |  | 12.85 | 0.003 | 0 | 1.18 |  |  | 1 | 1 | 1 |
| 2 | 81.1 | 9.94 | 13.1 | 18 |  |  |  | 0.002 | 0 | 1.32 |  |  | 0 | 1 | 1 |
| 1 | 71 | 11.24 | 13.9 | 6.87 |  |  |  |  | 0 |  |  |  | 0 | 0 | 0 |
| 1 | 81.1 | 12.03 | 11.5 | 17.08 |  |  | 18.18 |  | 0 |  |  |  | 1 | 1 | 0 |
| 2 | 64.4 | 7.05 | 12.1 | 6.6 |  |  |  |  | 0 |  |  |  | 0 | 1 | 0 |
| 2 | 78.6 | 9.34 | 12.2 | 13.73 |  |  |  |  | 0 |  |  |  | 0 | 1 | 0 |
| 2 | 80.1 | 9.71 | 12.3 | 21.13 |  |  |  |  | 0 |  |  |  | 0 | 0 | 0 |
| 1 | 79.1 | 10.22 | 11.2 | 18 |  |  | 3.47 | 0.005 | 0 | 0.81 |  |  | 1 | 1 | 0 |
| 1 | 73.4 | 10.59 | 11.5 | 9.63 |  |  |  |  | 0 |  |  |  | 0 | 0 | 0 |
| 1 | 76.6 | 10.22 | 11.7 | 16.99 |  |  |  |  | 0 |  |  |  | 0 | 1 | 0 |
| 1 | 76.4 | 10.41 | 12.1 | 14.95 |  |  |  | 0.002 | 0 | 0.96 |  |  | 1 | 1 | 0 |
| 1 | 81.5 | 9.70 | 13.3 | 19.02 |  |  | 27.73 | 0.005 | 0 | 0.75 |  |  | 1 | 1 | 0 |
| 2 | 80.2 | 11.07 | 11.6 | 23.72 |  |  | 6.5 | 0.005 | 0 | 0.79 |  |  | 1 | 1 | 0 |
| 2 | 82.1 | 9.72 | 12.6 | 20.93 |  |  | 23.37 | 0.11 | 0 | 0.81 |  |  | 1 | 1 | 1 |
| 2 | 78.3 | 9.43 | 12.4 | 16.53 |  |  | 9.09 | 0.003 | 0 | 1.11 |  |  | 1 | 1 | 0 |
| 2 | 67.1 | 7.62 | 11.3 | 9.53 |  |  |  |  | 0 |  |  |  | 0 | 1 | 0 |
| 1 | 66.1 | 8.22 | 11.7 | 7.03 |  |  |  |  | 0 |  |  |  | 0 | 0 | 0 |
| 1 | 78 | 10.19 | 11.5 | 17.41 |  |  | 12.66 | 0.002 | 0 | 1.25 |  |  | 1 | 1 | 0 |
| 2 | 77.1 | 9.46 | 8.9 | 13.7 |  |  | 28.74 | 0.003 | 0 | 1.28 |  |  | 1 | 1 | 0 |
| 2 | 90 | 13.00 | 12.8 | 20.17 |  |  | 23.89 | 0.002 | 0 | 0.98 |  |  | 1 | 1 | 1 |
| 2 | 81.5 | 11.21 | 12.6 | 16.79 |  |  | 4.64 | 0.003 | 0 |  |  |  | 1 | 1 | 1 |
| 1 | 85.6 | 11.91 | 12.9 | 23.85 |  |  | 16.56 |  | 0 |  |  |  | 1 | 1 | 0 |
| 1 | 90.5 | 12.63 | 12.2 | 23.43 |  |  | 18.03 | 0.003 | 0 |  |  |  | 1 | 1 | 0 |
| 1 | 84 | 11.04 | 11.6 | 22.05 |  |  | 24.28 | 0.206 | 0 |  |  |  | 1 | 1 | 0 |
| 1 | 86.5 | 11.84 | 13.5 | 20.86 |  |  | 40.85 | 0.002 | 0 |  |  |  | 1 | 1 | 1 |
| 1 | 69.6 | 7.90 | 10.4 | 9 |  |  |  |  | 0 |  |  |  | 0 | 1 | 0 |
| 1 | 71.1 | 9.65 | 13.1 | 11.86 |  |  |  |  | 0 |  |  |  | 0 | 1 | 1 |
| 2 | 74 | 10.01 | 11 | 11.2 |  |  |  |  | 0 |  |  |  | 0 | 1 | 0 |
| 2 | 71.9 | 8.82 | 13.1 | 12.32 |  |  |  |  | 0 |  |  |  | 1 | 1 | 1 |
| 2 | 68 | 9.64 | 12.7 | 6.74 |  |  |  |  | 0 |  |  |  | 0 | 0 | 0 |
| 2 | 73.7 | 8.65 | 12.1 | 10.09 |  |  |  |  | 0 |  |  |  | 0 | 1 | 1 |
| 2 | 84.5 | 11.27 | 12.1 | 11.99 |  |  | 6.55 | 0.002 | 0 |  |  |  | 1 | 1 | 0 |
| 1 | 80.3 | 10.46 | 13.2 | 17.05 |  |  | 25.05 | 0.002 | 0 |  |  |  | 0 | 1 | 0 |
| 2 | 81.2 | 11.43 | 13.5 | 22.51 |  |  | 30.29 | 0.003 | 0 |  |  |  | 1 | 1 | 1 |
| 1 | 69.1 | 8.54 | 13.1 | 9.17 |  |  |  |  | 0 |  |  |  | 0 | 1 | 0 |
| 1 | 74.1 | 10.69 | 12 | 10.58 |  |  |  |  | 0 |  |  |  | 0 | 0 | 0 |
| 1 | 85.1 | 11.24 | 13.7 | 21.39 |  |  | 19.85 | 0.005 | 0 |  |  |  | 1 | 1 | 1 |
| 2 | 82.1 | 9.99 | 12.1 | 21.09 |  |  |  |  | 0 |  |  |  | 1 | 1 | 1 |
| 2 | 74.5 | 9.45 | 12.9 | 11.99 |  |  |  |  | 0 |  |  |  | 1 | 1 | 0 |
| 1 | 72.9 | 9.55 | 10.9 | 12.02 |  |  |  |  | 0 |  |  |  | 1 | 1 | 0 |
| 1 | 70.6 | 7.47 | 11.2 | 7.75 |  |  |  |  | 0 |  |  |  | 0 | 1 | 0 |
| 2 | 72.1 | 9.10 | 11 | 11.66 |  |  |  |  | 0 |  |  |  | 0 | 1 | 0 |
| 2 | 77.3 | 10.16 | 10.9 | 20.4 |  |  | 7.01 | 0.388 | 0 |  |  |  | 1 | 1 | 0 |
| 1 | 81 | 10.79 | 11.7 | 20.83 |  |  | 60.62 | 0.004 | 0 |  |  |  | 1 | 1 | 0 |
| 1 | 73 | 7.97 | 12.1 | 6.31 |  |  |  |  | 0 |  |  |  | 0 | 0 | 0 |
| 2 | 77 | 10.27 | 12.7 | 10.94 |  |  |  |  | 0 |  |  |  | 1 | 1 | 1 |
| 2 | 86.2 | 12.10 | 11.4 | 20.63 |  |  |  |  | 0 |  |  |  | 0 | 1 | 0 |
| 2 | 69 | 6.99 | 10.7 | 10.28 |  |  |  |  | 0 |  |  |  | 0 | 1 | 0 |
| 2 | 79.4 | 9.55 | 11.1 | 10.64 |  |  |  |  | 0 |  |  |  | 1 | 1 | 0 |
| 1 | 71.9 | 7.96 | 10.1 | 11.6 |  |  |  |  | 0 |  |  |  | 0 | 1 | 0 |
| 1 | 82.1 | 13.50 | 13.1 | 17.94 |  |  | 22.37 | 0.004 | 0 |  |  |  | 1 | 1 | 1 |
| 1 | 75.9 | 10.32 | 12.4 | 13.4 |  |  |  |  | 0 |  |  |  | 0 | 1 | 0 |
| 1 | 85 | 13.41 | 12.8 | 20.93 |  |  | 6.99 |  | 0 |  |  |  | 1 | 1 | 0 |
| 1 | 79.5 | 8.90 | 14.8 | 18.6 |  |  | 23.94 | 0.003 | 0 |  |  |  | 0 | 1 | 0 |
| 2 | 76.3 | 11.13 | 11.9 | 17.12 |  |  | 13.08 | 0.004 | 0 |  |  |  | 1 | 1 | 1 |
| 2 | 68 | 7.64 | 11.7 | 10.74 |  |  |  |  | 0 |  |  |  | 1 | 1 | 1 |
| 2 | 70.1 | 9.41 | 8.6 | 9.59 |  |  |  |  | 0 |  |  |  | 0 | 0 | 0 |
| 2 | 75 | 10.04 | 9.8 | 12.98 |  |  |  |  | 0 |  |  |  | 0 | 1 | 0 |
| 1 | 71.2 | 9.19 | 12.5 | 8.51 |  |  | 37.3 |  | 0 |  |  |  | 0 | 1 | 0 |
| 2 | 80.3 | 10.25 | 13.3 | 19.38 |  |  |  |  | 0 |  |  |  | 1 | 1 | 0 |
| 1 | 80 | 8.37 | 11.7 | 17.38 |  |  | 20.01 | 0.002 | 0 |  |  |  | 0 | 1 | 0 |
| 2 | 70 | 7.60 | 12.9 | 9.43 |  |  |  |  | 0 |  |  |  | 0 | 1 | 0 |
| 1 | 73 | 10.11 | 11.8 | 10.78 |  |  |  |  | 0 |  |  |  | 0 | 1 | 0 |
| 2 | 70 | 7.38 | 12.1 | 9.79 |  |  |  |  | 0 |  |  |  | 1 | 1 | 0 |
| 2 | 79 | 9.36 | 10.6 | 15.74 |  |  | 22.07 | 0.002 | 0 |  |  |  | 1 | 0 | 1 |
| 1 | 73 | 9.43 | 12.2 | 10.12 |  |  |  |  | 0 |  |  |  | 0 | 1 | 1 |
| 2 | 81.1 | 10.69 | 13.8 | 19.88 |  |  | 26.97 | 0.003 | 0 |  |  |  | 0 | 0 | 0 |
| 2 | 82.6 | 11.28 | 11.9 | 19.94 |  |  | 20.27 | 0.003 | 0 |  |  |  | 1 | 1 | 0 |
| 1 | 84.7 | 12.70 | 12.8 | 21.95 |  |  | 15.41 | 0.002 | 0 |  |  |  | 1 | 1 | 1 |
| 2 | 80.3 | 9.23 | 13.3 | 20.34 |  |  | 15.9 |  | 0 |  |  |  | 1 | 1 | 1 |
| 2 | 66.6 | 7.49 | 11.9 | 6.14 |  |  | 5.62 | 0.015 | 0 |  |  |  | 0 | 0 | 0 |
| 2 | 75.1 | 9.29 | 12.4 | 16.72 |  |  |  |  | 0 |  |  |  | 0 | 1 | 1 |
| 2 | 74.3 | 8.13 | 12 | 12.81 |  |  |  |  | 0 |  |  |  | 1 | 1 | 0 |
| 1 | 72 | 9.07 | 11.8 | 9.72 |  |  |  |  | 0 |  |  |  | 1 | 1 | 0 |
| 2 | 66.1 | 8.88 | 13.3 | 7.69 |  |  |  |  | 0 |  |  |  | 0 | 1 | 0 |
| 1 | 69.6 | 8.34 | 10.8 | 9.72 |  |  | 8.7 | 0.002 | 0 |  |  |  | 0 | 0 | 0 |
| 1 | 78.1 | 12.13 | 11.4 | 14.06 |  |  |  |  | 0 |  |  |  | 1 | 1 | 0 |
| 1 | 83.4 | 11.20 | 12.2 | 22.7 |  |  | 11.07 | 0.205 | 0 |  |  |  | 1 | 1 | 0 |
| 2 | 80.2 | 9.71 | 13 | 21.82 |  |  |  |  | 0 |  |  |  | 1 | 1 | 0 |
| 2 | 76.5 | 8.57 | 11.3 | 19.06 |  |  | 4.6 | 0.004 | 0 |  |  |  | 1 | 1 | 0 |
| 1 | 86.1 | 13.25 | 15.3 | 22.9 |  |  | 12.74 | 0.003 | 0 |  |  |  | 0 | 1 | 0 |
| 2 | 80.1 | 9.94 | 11.7 | 19.98 |  |  | 21.64 | 0.004 | 0 |  |  |  | 0 | 1 | 0 |
| 2 | 69.6 | 8.20 | 11 | 10.38 |  |  |  |  | 0 |  |  |  | 1 | 1 | 1 |
| 1 | 75.1 | 9.70 | 12 | 9.72 |  |  |  |  | 0 |  |  |  | 0 | 1 | 0 |
| 1 | 74.2 | 9.88 | 12.4 | 10.91 |  |  |  |  | 0 |  |  |  | 0 | 1 | 0 |
| 2 | 62 | 6.40 | 12.8 | 6.44 |  |  |  |  | 1 |  |  | 0 | 0 | 1 | 0 |
| 2 | 77 | 8.30 | 11 | 9 |  |  |  |  | 1 |  |  | 0 | 1 | 1 | 1 |
| 1 | 75 | 8.60 | 12.3 | 9.46 |  |  |  |  | 1 |  |  | 0 | 0 | 0 | 0 |
| 1 | 72.3 | 9.57 | 12.7 | 8.57 |  |  |  |  | 1 |  |  | 0 | 0 | 1 | 0 |
| 1 | 72 | 8.90 | 10.7 | 9.49 |  |  |  |  | 1 |  |  | 0 | 0 | 1 | 0 |
| 2 | 68 | 6.70 | 12.9 | 7.03 |  |  |  |  | 1 |  |  | 0 | 0 | 0 | 0 |
| 1 | 71 | 7.90 | 12.1 | 11.43 |  |  |  |  | 1 |  |  | 0 | 0 | 0 | 1 |
| 2 | 70 | 8.13 | 8.7 | 6.24 |  |  |  |  | 1 |  |  | 0 | 0 | 0 | 0 |
| 1 | 70 | 8.21 | 12 | 7.66 |  |  |  |  | 1 |  |  | 0 | 1 | 1 | 0 |
| 2 | 77 | 9.60 | 10.7 | 13.17 |  |  |  |  | 1 |  |  | 7 | 0 | 1 | 0 |
| 1 | 72 | 8.61 | 13.1 | 10.18 |  |  |  |  | 1 |  |  | 6 | 1 | 1 | 1 |
| 2 | 72 | 7.52 | 12.4 | 10.28 |  |  |  |  | 1 |  |  | 5 | 1 | 1 | 1 |
| 1 | 73 | 8.70 | 11.7 | 8.71 |  |  |  |  | 1 |  |  | 7 | 0 | 1 | 0 |
| 1 | 67.8 | 7.78 | 11.6 | 8.61 |  |  |  |  | 1 |  |  | 6 | 0 | 1 | 0 |
| 2 | 68.5 | 8.76 | 11.7 | 6.67 |  |  |  |  | 1 |  |  | 5 | 0 | 0 | 0 |
| 1 | 72.5 | 9.39 | 12.7 | 12.81 |  |  |  |  | 1 |  |  | 7 | 0 | 1 | 0 |
| 2 | 69.5 | 7.93 | 10.2 | 9.95 |  |  |  |  | 1 |  |  | 4 | 1 | 1 | 1 |
| 1 | 74 | 8.40 | 12.9 | 11.1 |  |  |  |  | 1 |  |  | 7 | 0 | 1 | 1 |
| 2 | 75 | 9.55 | 12.4 | 10.28 |  |  |  |  | 1 |  |  | 7 | 1 | 1 | 0 |
| 1 | 76 | 9.10 | 13.6 | 11.07 |  |  |  |  | 1 |  |  | 6 | 1 | 1 | 1 |
| 1 | 78 | 9.10 | 14 | 11.07 |  |  |  |  | 1 |  |  | 6 | 1 | 1 | 1 |
| 2 | 76 | 9.15 | 11.6 | 11.1 |  |  |  |  | 1 |  |  | 6 | 1 | 1 | 1 |
| 1 | 72.7 | 8.00 | 11.6 | 11.43 |  |  |  |  | 1 |  |  | 7 | 1 | 1 | 1 |
| 2 | 72.6 | 8.02 | 11.8 | 9.89 |  |  |  |  | 1 |  |  | 6 | 0 | 1 | 0 |
| 1 | 77.9 | 9.55 | 12.7 | 11.43 |  |  |  |  | 1 |  |  | 7 | 1 | 1 | 1 |
| 1 | 81.2 | 9.74 | 13.3 | 18.92 |  |  |  |  | 1 |  |  | 7 | 1 | 1 | 1 |
| 2 | 73.6 | 8.09 | 11.2 | 11.79 |  |  |  |  | 1 |  |  | 7 | 0 | 1 | 0 |
| 2 | 72.1 | 7.11 | 11.7 | 8.28 |  |  |  |  | 1 |  |  | 7 | 1 | 1 | 0 |
| 2 | 70.5 | 8.74 | 13.4 | 7.39 |  |  |  |  | 1 |  |  | 3 | 1 | 1 | 0 |
| 2 | 74.6 | 8.13 | 11.8 | 9.99 |  |  |  |  | 1 |  |  | 6 | 1 | 1 | 0 |
| 1 | 76.5 | 9.01 | 12 | 10.55 |  |  |  |  | 1 |  |  | 7 | 1 | 1 | 1 |
| 1 | 68.2 | 8.03 | 13.4 | 7.89 |  |  |  |  | 1 |  |  | 7 | 0 | 0 | 0 |
| 1 | 74.2 | 9.99 | 9.8 | 8.94 |  |  |  |  | 1 |  |  | 7 | 0 | 1 | 0 |
| 2 | 73.5 | 7.87 | 10.7 | 8.02 |  |  |  |  | 1 |  |  | 7 | 0 | 0 | 0 |
| 2 | 71.8 | 7.93 | 14.1 | 10.28 |  |  |  |  | 1 |  |  | 7 | 0 | 0 | 0 |
| 1 | 80.1 | 12.38 | 11.9 | 11.5 |  |  |  |  | 1 |  |  | 1 | 0 | 1 | 0 |
| 1 | 73 | 9.00 | 11.6 | 9.46 |  |  |  |  | 1 |  |  | 5 | 1 | 1 | 1 |
| 2 | 81 | 9.60 | 13.8 | 13.83 |  |  |  |  | 1 |  |  | 7 | 1 | 1 | 1 |
| 1 | 76 | 7.65 | 10.4 | 10.18 |  |  |  |  | 1 |  |  | 7 | 0 | 1 | 0 |
| 1 | 75 | 8.60 | 12.4 | 10.64 |  |  |  |  | 1 |  |  | 5 | 1 | 1 | 1 |
| 1 | 71 | 7.50 | 11 | 6.31 |  |  |  |  | 1 |  |  | 7 | 0 | 1 | 0 |
| 1 | 71 | 7.60 | 13.4 | 7.29 |  |  |  |  | 1 |  |  | 4 | 0 | 0 | 0 |
| 1 | 73 | 9.20 | 11.8 | 9.36 |  |  |  |  | 1 |  |  | 7 | 0 | 1 | 0 |
| 2 | 68 | 8.20 | 12.5 | 9.1 |  |  |  |  | 1 |  |  | 5 | 1 | 1 | 1 |
| 2 | 76 | 9.00 | 13.7 | 13.83 |  |  |  |  | 1 |  |  | 6 | 0 | 0 | 0 |
| 2 | 67 | 7.00 | 11.6 | 6.18 |  |  |  |  | 1 |  |  | 4 | 1 | 1 | 0 |
| 2 | 68 | 8.40 | 12.5 | 6.57 |  |  |  |  | 1 |  |  | 7 | 0 | 1 | 0 |
| 1 | 71 | 7.60 | 11.5 | 7.98 |  |  |  |  | 1 |  |  | 5 | 0 | 1 | 0 |
| 2 | 68 | 6.31 | 11.7 | 7.75 |  |  |  |  | 1 |  |  | 7 | 0 | 0 | 0 |
| 1 | 70 | 7.93 | 10.4 | 7.49 |  |  |  |  | 1 |  |  | 7 | 0 | 1 | 0 |
| 2 | 71 | 7.50 | 13.3 | 7.56 |  |  |  |  | 1 |  |  | 7 | 0 | 1 | 0 |
| 1 | 76 | 8.60 | 9.4 | 7.39 |  |  |  |  | 1 |  |  | 7 | 0 | 0 | 0 |
| 2 | 70 | 7.62 | 11.6 | 8.87 |  |  |  |  | 1 |  |  | 7 | 1 | 1 | 0 |
| 2 | 72 | 7.40 | 12.4 | 8.8 |  |  |  |  | 1 |  |  | 6 | 1 | 1 | 0 |
| 1 | 70 | 8.10 | 11.1 | 9.23 |  |  |  |  | 1 |  |  | 5 | 1 | 1 | 1 |
| 1 | 72 | 8.31 | 13.1 | 7.52 |  |  |  |  | 1 |  |  | 6 | 0 | 0 | 0 |
| 1 | 75 | 10.30 | 11.6 | 10.48 |  |  |  |  | 1 |  |  | 5 | 1 | 1 | 1 |
| 2 | 68 | 6.60 | 11.9 | 8.08 |  |  |  |  | 1 |  |  | 4 | 0 | 1 | 1 |
| 2 | 74 | 7.60 | 11.4 | 11.17 |  |  |  |  | 1 |  |  | 7 | 1 | 1 | 1 |
| 2 | 69 | 8.05 | 12.8 | 9.63 |  |  |  |  | 1 |  |  | 7 | 1 | 1 | 0 |
| 1 | 85 | 9.71 | 12.9 | 21.95 |  |  |  |  | 1 |  |  | 7 | 0 | 1 | 0 |
| 1 | 76 | 10.78 | 11.8 | 9.26 |  |  |  |  | 1 |  |  | 6 | 1 | 1 | 0 |
| 1 | 78 | 8.05 | 12.3 | 11.5 |  |  |  |  | 1 |  |  | 4 | 1 | 0 | 1 |
| 1 | 74 | 10.19 | 12.5 | 10.58 |  |  |  |  | 1 |  |  | 0 | 0 | 1 | 0 |
| 2 | 72 | 10.04 | 11.7 | 10.74 |  |  |  |  | 1 |  |  | 0 | 0 | 1 | 0 |
| 2 | 68 | 7.84 | 9.4 | 7.85 |  |  |  |  | 1 |  |  | 0 | 1 | 1 | 1 |
| 1 | 70 | 9.52 | 10.5 | 10.84 |  |  |  |  | 1 |  |  | 0 | 0 | 0 | 0 |
| 2 | 74.5 | 10.37 | 11 | 11.24 |  |  |  |  | 1 |  |  | 0 | 0 | 0 | 0 |
| 2 | 79 | 10.38 | 13.7 | 8.31 |  |  |  |  | 1 |  |  | 0 | 1 | 1 | 0 |
| 1 | 66 | 9.03 | 11.4 | 7.13 |  |  |  |  | 1 |  |  | 0 | 0 | 1 | 1 |
| 2 | 69 | 8.91 | 10.6 | 10.18 |  |  |  |  | 1 |  |  | 0 | 1 | 1 | 1 |
| 1 | 65 | 8.15 | 11.1 | 7 |  |  |  |  | 1 |  |  | 0 | 0 | 0 | 0 |
| 1 | 73.3 | 10.08 | 10 | 11.2 |  |  |  |  | 1 |  |  | 0 | 0 | 1 | 0 |
| 1 | 75 | 11.27 | 11.1 | 11.89 |  |  |  |  | 1 |  |  | 0 | 1 | 1 | 1 |
| 1 | 70 | 8.42 | 9.8 | 8.34 |  |  |  |  | 1 |  |  | 0 | 0 | 0 | 0 |
| 1 | 67.5 | 7.82 | 10.4 | 8.57 |  |  |  |  | 1 |  |  | 0 | 0 | 1 | 0 |
| 1 | 71 | 9.94 | 10.8 | 6.64 |  |  |  |  | 1 |  |  | 0 | 0 | 0 | 0 |
| 1 | 71 | 10.14 | 9.3 | 9.89 |  |  |  |  | 1 |  |  | 0 | 1 | 1 | 0 |
| 2 | 70.5 | 9.03 | 10.7 | 9.79 |  |  |  |  | 1 |  |  | 0 | 0 | 0 | 0 |
| 1 | 69 | 8.82 | 10.6 | 6.37 |  |  |  |  | 1 |  |  | 0 | 0 | 0 | 0 |
| 1 | 73 | 11.40 | 9.8 | 11.4 |  |  |  |  | 1 |  |  | 0 | 0 | 1 | 0 |
| 2 | 67.5 | 8.58 | 10.4 | 8.25 |  |  |  |  | 1 |  |  | 0 | 1 | 1 | 1 |
| 2 | 71 | 8.88 | 11.5 | 8.9 |  |  |  |  | 1 |  |  | 0 | 1 | 1 | 0 |
| 1 | 68.5 | 8.66 | 10.9 | 9.69 |  |  |  |  | 1 |  |  | 0 | 1 | 1 | 0 |
| 1 | 70 | 8.93 | 10.3 | 8.77 |  |  |  |  | 1 |  |  | 0 | 1 | 1 | 0 |
| 2 | 66 | 8.70 | 11.6 | 8.51 |  |  |  |  | 1 |  |  | 0 | 0 | 0 | 0 |
| 1 | 70 | 8.10 | 11.8 | 7.2 |  |  |  |  | 1 |  |  | 0 | 0 | 1 | 0 |
| 1 | 74 | 9.00 | 10.3 | 10.81 |  |  |  |  | 1 |  |  | 0 | 0 | 1 | 0 |
| 1 | 74 | 9.95 | 11.2 | 8.57 |  |  |  |  | 1 |  |  | 6 | 1 | 1 | 1 |
| 1 | 74.5 | 10.46 | 13.1 | 10.91 |  |  |  |  | 1 |  |  | 7 | 1 | 1 | 1 |
| 1 | 72.5 | 9.53 | 12.7 | 11.43 |  |  |  |  | 1 |  |  | 7 | 1 | 1 | 1 |
| 1 | 75 | 9.99 | 11.1 | 11.89 |  |  |  |  | 1 |  |  | 7 | 0 | 0 | 0 |
| 1 | 72.5 | 10.68 | 11.3 | 11.01 |  |  |  |  | 1 |  |  | 7 | 1 | 1 | 1 |
| 1 | 70 | 8.74 | 6.9 | 10.15 |  |  |  |  | 1 |  |  | 7 | 1 | 1 | 1 |
| 1 | 72 | 9.32 | 8.9 | 9.43 |  |  |  |  | 1 |  |  | 7 | 1 | 1 | 1 |
| 1 | 66 | 8.19 | 9.7 | 6.37 |  |  |  |  | 1 |  |  | 4 | 0 | 0 | 1 |
| 1 | 73 | 9.82 | 12.1 | 11.24 |  |  |  |  | 1 |  |  | 7 | 0 | 1 | 0 |
| 1 | 66 | 7.95 | 11.4 | 7.59 |  |  |  |  | 1 |  |  | 4 | 0 | 1 | 0 |
| 1 | 71.5 | 9.76 | 10.8 | 11.27 |  |  |  |  | 1 |  |  | 6 | 0 | 1 | 0 |
| 1 | 73 | 9.91 | 11.1 | 9.03 |  |  |  |  | 1 |  |  | 7 | 0 | 0 | 0 |
| 1 | 73 | 10.14 | 12.6 | 10.35 |  |  |  |  | 1 |  |  | 7 | 0 | 0 | 0 |
| 1 | 78 | 13.60 | 8.4 | 11.5 |  |  |  |  | 1 |  |  | 7 | 1 | 1 | 0 |
| 2 | 67 | 5.10 | 10.1 | 8.44 |  |  |  |  | 1 |  |  | 7 | 0 | 1 | 0 |
| 1 | 76 | 10.50 | 9.4 | 9.95 |  |  |  |  | 1 |  |  | 4 | 1 | 1 | 1 |
| 2 | 71 | 10.50 | 10.3 | 9.59 |  |  |  |  | 1 |  |  | 6 | 1 | 1 | 0 |
| 1 | 76 | 11.10 | 11.2 | 12.16 |  |  |  |  | 1 |  |  | 0 | 1 | 1 | 0 |
| 2 | 74 | 9.88 | 12.3 | 12.22 |  |  |  |  | 1 |  |  | 0 | 1 | 1 | 1 |
| 1 | 79 | 11.21 | 10.3 | 18.04 |  |  |  |  | 1 |  |  | 0 | 1 | 1 | 1 |
| 1 | 74.5 | 9.12 | 13 | 13.14 |  |  |  |  | 1 |  |  | 0 | 1 | 1 | 1 |
| 1 | 79.5 | 11.99 | 12.5 | 12.68 |  |  |  |  | 1 |  |  | 0 | 1 | 1 | 1 |
| 2 | 72 | 8.33 | 9.5 | 12.25 |  |  |  |  | 1 |  |  | 0 | 1 | 1 | 1 |
| 2 | 78 | 10.19 | 10.7 | 16.2 |  |  |  |  | 1 |  |  | 0 | 1 | 1 | 1 |
| 1 | 82 | 11.54 | 10.3 | 16.99 |  |  |  |  | 1 |  |  | 0 | 1 | 0 | 1 |
| 2 | 76.8 | 9.79 | 11 | 15.87 |  |  |  |  | 1 |  |  | 0 | 1 | 0 | 1 |
| 2 | 82.5 | 10.80 | 11.4 | 21.06 |  |  |  |  | 1 |  |  | 0 | 1 | 1 | 1 |
| 1 | 78.5 | 10.70 | 10.6 | 14.29 |  |  |  |  | 1 |  |  | 0 | 1 | 1 | 1 |
| 1 | 76 | 9.37 | 11.7 | 13.86 |  |  |  |  | 1 |  |  | 0 | 1 | 1 | 0 |
| 2 | 79 | 9.89 | 11.7 | 21.42 |  |  |  |  | 1 |  |  | 0 | 0 | 1 | 0 |
| 1 | 73 | 9.40 | 10.3 | 13.21 |  |  |  |  | 1 |  |  | 0 | 0 | 0 | 0 |
| 1 | 72 | 9.60 | 10.3 | 12.06 |  |  |  |  | 1 |  |  | 0 | 1 | 1 | 0 |
| 2 | 72 | 8.32 | 12.1 | 12.62 |  |  |  |  | 1 |  |  | 0 | 0 | 0 | 0 |
| 2 | 87 | 12.98 | 9.2 | 23.69 |  |  |  |  | 1 |  |  | 0 | 1 | 1 | 1 |
| 1 | 83 | 12.50 | 12.1 | 17.18 |  |  |  |  | 1 |  |  | 0 | 1 | 1 | 0 |
| 2 | 82 | 11.50 | 11.3 | 22.77 |  |  |  |  | 1 |  |  | 0 | 0 | 0 | 1 |
| 2 | 77.5 | 7.52 | 12.5 | 18.43 |  |  |  |  | 1 |  |  | 0 | 1 | 1 | 1 |
| 2 | 75 | 9.31 | 13.2 | 16.53 |  |  |  |  | 1 |  |  | 7 | 0 | 0 | 0 |
| 2 | 77.7 | 10.40 | 12 | 16.03 |  |  |  |  | 1 |  |  | 7 | 1 | 1 | 1 |
| 2 | 79.5 | 10.14 | 13.2 | 18.4 |  |  |  |  | 1 |  |  | 7 | 0 | 1 | 0 |
| 2 | 73.5 | 10.32 | 11.4 | 12.22 |  |  |  |  | 1 |  |  | 7 | 1 | 1 | 1 |
| 2 | 78.5 | 11.27 | 12 | 17.68 |  |  |  |  | 1 |  |  | 7 | 0 | 1 | 0 |
| 1 | 77.5 | 8.92 | 12.1 | 13.08 |  |  |  |  | 1 |  |  | 7 | 1 | 1 | 1 |
| 1 | 79 | 11.01 | 11 | 15.67 |  |  |  |  | 1 |  |  | 6 | 1 | 1 | 1 |
| 2 | 83 | 10.54 | 13.1 | 20.01 |  |  |  |  | 1 |  |  | 7 | 1 | 1 | 1 |
| 1 | 77.5 | 10.27 | 10.8 | 14.13 |  |  |  |  | 1 |  |  | 7 | 0 | 0 | 1 |
| 1 | 82 | 11.18 | 11.9 | 15.54 |  |  |  |  | 1 |  |  | 7 | 1 | 1 | 0 |
| 1 | 77 | 10.20 | 10.6 | 13.86 |  |  |  |  | 1 |  |  | 7 | 1 | 1 | 0 |
| 1 | 82 | 11.74 | 11.3 | 21.45 |  |  |  |  | 1 |  |  | 7 | 1 | 1 | 0 |
| 1 | 81.5 | 12.28 | 11 | 19.38 |  |  |  |  | 1 |  |  | 6 | 1 | 1 | 1 |
| 2 | 74 | 8.88 | 11.2 | 12.55 |  |  |  |  | 1 |  |  | 7 | 0 | 1 | 0 |
| 2 | 73 | 10.01 | 12.3 | 14.23 |  |  |  |  | 1 |  |  | 7 | 1 | 1 | 1 |
| 2 | 81 | 10.30 | 13.5 | 21.42 |  |  |  |  | 1 |  |  | 7 | 1 | 0 | 1 |
| 1 | 78.5 | 10.18 | 12.3 | 18.3 |  |  |  |  | 1 |  |  | 0 | 1 | 0 | 0 |
| 1 | 77 | 9.92 | 11.8 | 17.64 |  |  |  |  | 1 |  |  | 7 | 1 | 1 | 1 |
| 2 | 80 | 11.01 | 11.5 | 22.41 |  |  |  |  | 1 |  |  | 7 | 1 | 1 | 0 |
| 1 | 85 | 12.99 | 12.5 | 17.64 |  |  |  |  | 1 |  |  | 6 | 0 | 1 | 0 |
| 2 | 79 | 12.23 | 13.8 | 19.12 |  |  |  |  | 1 |  |  | 7 | 0 | 1 | 0 |
| 2 | 80 | 9.95 | 13 | 21.55 |  |  |  |  | 1 |  |  | 7 | 1 | 1 | 1 |
| 1 | 79 | 11.00 | 13.2 | 18.17 |  |  |  |  | 1 |  |  | 7 | 1 | 1 | 1 |
| 2 | 78 | 9.50 | 12 | 16.13 |  |  |  |  | 1 |  |  | 6 | 1 | 1 | 1 |
| 1 | 88 | 12.60 | 9.6 | 15.97 |  |  |  |  | 1 |  |  | 7 | 1 | 1 | 1 |
| 2 | 82 | 9.50 | 14.4 | 21.55 |  |  |  |  | 1 |  |  | 7 | 1 | 1 | 1 |
| 2 | 76 | 7.20 | 12.5 | 13.7 |  |  |  |  | 1 |  |  | 7 | 0 | 1 | 0 |
| 1 | 79 | 11.89 | 12 | 19.02 |  |  |  |  | 1 |  |  | 0 | 1 | 1 | 0 |
| 1 | 78 | 11.30 | 13.1 | 17.84 | 28.92 | 445.91 | 72.15 | 1.216 | 1 | 1.11 | 43.26 | 0 | 1 | 1 | 1 |
| 1 | 78 | 10.50 | 11.3 | 18.99 | 10.55 | 126.36 | 13.89 | 0.023 | 1 | 0.79 | 54.93 | 7 | 1 | 1 | 1 |
| 1 | 84 | 11.90 | 11.8 | 21.65 | 15.4 | 130.26 | 12.85 | 0.015 | 1 | 0.97 |  | 7 | 0 | 1 | 0 |
| 2 | 80 | 11.60 | 12.7 | 19.19 |  | 287.93 | 35.26 | 0.116 | 1 | 1.50 | 72.57 | 7 | 1 | 1 | 1 |
| 1 | 84 | 12.50 | 12.7 | 20.53 |  |  |  |  | 1 |  |  | 7 | 0 | 1 | 0 |
| 2 | 87 | 11.85 | 12.9 | 18.92 | 19.42 | 535.74 | 31.42 | 0.014 | 1 | 1.28 | 49.81 | 7 | 0 | 1 | 0 |
| 2 | 76.5 | 10.11 | 12.4 | 17.45 | 8.7 | 217.57 | 15.36 | 0.038 | 1 | 1.45 | 47.04 | 7 | 1 | 1 | 1 |
| 2 | 78 | 9.88 | 12.7 | 19.88 | 15.55 | 677.41 | 23.95 | 0.023 | 1 | 1.70 | 95.54 | 7 | 0 | 1 | 0 |
| 1 | 80 | 10.95 | 11.5 | 15.93 | 13.11 | 267.7 | 6.1 | 0.302 | 1 | 1.17 | 67.03 | 7 | 1 | 1 | 1 |
| 2 | 83 | 12.63 | 12 | 15.93 | 4.59 | 107.41 | 4.38 | 0.024 | 1 | 1.02 | 38.49 | 7 | 0 | 1 | 1 |
| 2 | 78 | 9.69 | 11.8 | 20.76 | 9.82 | 192.13 | 16.45 | 0.056 | 1 | 1.52 | 43.03 | 0 | 1 | 1 | 1 |
| 2 | 74 | 9.27 | 12.3 | 15.21 | 35.99 | 816.05 | 44.68 | 0.014 | 1 |  | 76.76 | 7 | 0 | 1 | 0 |
| 1 | 80 | 10.00 | 11.7 | 19.02 | 12.12 | 787.37 | 4.04 | 0.008 | 1 | 1.92 | 72.47 | 0 | 0 | 0 | 1 |
| 1 | 80 | 10.49 | 10.8 | 16.07 | 10.53 | 234.52 | 6.3 | 0.068 | 1 | 1.14 | 46.16 | 0 | 1 | 1 | 0 |
| 1 | 77 | 10.99 | 11.3 | 16.59 | 14.31 | 909.21 | 43.38 | 0.038 | 1 | 1.52 | 74.19 | 7 | 0 | 1 | 0 |
| 2 | 76 | 11.14 | 12.6 | 15.84 | 15.27 | 424.13 | 18.1 | 0.141 | 1 | 1.36 | 92.16 | 0 | 0 | 0 | 0 |
| 1 | 75.5 | 9.74 | 9.6 | 16.23 | 18.03 | 247.01 | 2.41 | 0.071 | 1 | 1.11 | 54.68 | 7 | 0 | 0 | 1 |
| 2 | 80.5 | 12.70 | 11.4 | 21.19 | 9.04 | 278.39 | 3.6 | 0.109 | 1 | 1.19 | 58.15 | 7 | 1 | 1 | 0 |
| 1 | 80 | 11.50 | 10.9 | 19.84 | 16.33 | 544.41 | 54.84 | 0.275 | 1 | 0.80 | 30.40 | 0 | 1 | 1 | 1 |
| 2 | 83 | 10.68 | 12.1 | 21.65 |  |  |  |  | 1 | 1.76 |  | 7 | 1 | 1 | 0 |
| 1 | 74 | 10.64 | 11.6 | 14.88 | 9.23 | 247.01 | 3.93 | 0.026 | 1 | 1.32 | 33.60 | 0 | 0 | 1 | 0 |
| 1 | 83 | 12.61 | 11.7 | 22.54 | 8.7 | 202.37 | 4.06 | 0.01 | 1 | 1.04 | 28.33 | 7 | 1 | 1 | 1 |
| 2 | 75 | 8.91 | 13 | 14.42 | 13.39 | 571.18 | 15.48 | 0.052 | 1 | 1.72 | 70.31 | 0 | 0 | 1 | 0 |
| 1 | 76 | 11.15 | 12.9 | 16.99 | 17.8 | 954.42 |  | 0.047 | 1 | 1.67 | 45.93 | 5 | 0 | 1 | 0 |
| 1 | 81 | 10.57 | 11.2 | 20.04 | 18.03 | 391.28 | 15.29 | 0.023 | 1 | 1.69 | 44.82 | 7 | 0 | 1 | 1 |
| 1 | 86.5 | 12.36 | 13.1 | 15.77 | 6.49 | 381.3 | 13.41 | 0.013 | 1 | 1.37 | 56.34 | 0 | 1 | 1 | 0 |
| 2 | 72.3 | 8.97 | 11.6 | 12.78 | 17.33 | 139.15 | 2.18 | 0.006 | 1 | 0.91 |  | 0 | 1 | 1 | 0 |
| 2 | 81 | 10.76 | 12.6 | 21.03 | 9.98 | 306.15 | 15.59 | 0.179 | 1 | 0.71 | 39.58 | 7 | 1 | 1 | 1 |
| 2 | 82.5 | 11.95 | 13.4 | 19.38 | 10.33 | 328.03 | 13.22 | 0.03 | 1 | 1.15 |  | 0 | 1 | 1 | 1 |
| 1 | 80 | 10.12 | 10.9 | 21.16 |  |  |  |  | 1 | 0.80 |  | 0 | 1 | 1 | 0 |
| 2 | 83 | 10.78 | 11.2 | 16.07 | 12.85 | 219.96 | 55.63 | 0.048 | 1 | 1.70 | 115.91 | 7 | 0 | 1 | 1 |
| 1 | 80 | 11.16 | 12.1 | 16.33 | 14.25 | 311.89 | 10.05 | 0.031 | 1 | 1.46 | 53.19 | 7 | 0 | 0 | 1 |
| 2 | 74 | 9.79 | 12.8 | 7.03 | 13.05 | 198.93 | 2.54 | 0.111 | 1 | 1.27 | 63.73 | 7 | 0 | 1 | 0 |
| 1 | 86 | 12.01 | 12 | 22.67 | 14.57 | 648.57 | 30.3 | 0.04 | 1 | 0.96 | 49.99 | 7 | 1 | 1 | 1 |
| 2 | 85 | 11.00 | 12.2 | 22.77 | 27.7 | 381.3 | 19.51 | 0.014 | 1 | 1.06 |  | 7 | 0 | 1 | 1 |
| 1 | 77 | 9.85 | 12.3 | 16.1 | 6.98 | 143.16 | 10.4 | 0.019 | 1 | 0.62 | 23.75 | 0 | 1 | 1 | 1 |
| 2 | 77 | 9.50 | 11.8 | 17.64 |  | 1461.98 | 24.11 | 0.026 | 1 | 1.30 | 66.07 | 0 | 1 | 1 | 1 |
| 1 | 84 | 11.75 | 12.3 | 22.11 | 18.46 | 704.73 | 27.23 | 0.022 | 1 | 1.64 | 71.57 | 7 | 0 | 1 | 0 |
| 2 | 77.5 | 9.85 | 11.7 | 20.27 | 21 | 267.7 | 46.05 | 0.275 | 1 | 1.00 | 68.99 | 0 | 1 | 1 | 1 |
| 1 | 77 | 9.76 | 11.7 | 14.82 | 13.66 | 682.79 | 7.03 | 0.193 | 1 | 1.27 | 92.44 | 0 | 1 | 1 | 0 |
| 2 | 80 | 11.60 | 12.9 | 17.28 | 11.34 | 194.38 | 7.17 | 0.046 | 1 | 1.56 | 91.20 | 0 | 1 | 1 | 1 |
| 1 | 89 | 12.44 | 13 | 19.55 | 13.42 | 183.21 | 13.23 | 0.025 | 1 | 1.02 | 53.97 | 0 | 1 | 1 | 1 |
| 1 | 84 | 11.65 | 12.5 | 23.29 | 8.69 | 510.43 | 32.82 | 0.329 | 1 | 0.99 | 69.62 | 7 | 0 | 1 | 1 |
| 2 | 79.5 | 9.81 | 12.4 | 19.12 | 10.68 | 633.35 | 8.71 | 0.161 | 1 | 1.30 | 30.12 | 7 | 1 | 1 | 1 |
| 2 | 83 | 11.29 | 12.9 | 22.47 | 16.95 | 931.5 | 20.39 | 0.045 | 1 | 1.50 | 51.22 | 7 | 1 | 1 | 0 |
| 1 | 81.5 | 11.22 | 12.6 | 19.15 | 8.18 | 520.85 | 6.03 | 0.027 | 1 | 1.42 | 69.50 | 0 | 1 | 1 | 1 |
| 1 | 82 | 10.77 | 13.1 | 19.12 | 11.01 | 287.93 | 22.02 | 0.112 | 1 | 0.97 | 50.82 | 7 | 1 | 1 | 1 |
| 1 | 75 | 9.08 | 9.6 | 12.98 | 18.24 | 215.2 | 1.47 | 0.018 | 1 | 1.16 | 56.59 | 6 | 0 | 0 | 1 |
| 2 | 82 | 11.66 | 12.1 | 19.75 | 11.52 | 245.75 | 11.71 | 0.481 | 1 | 1.11 | 44.06 | 6 | 1 | 1 | 0 |
| 1 | 76.5 | 9.88 | 10.9 | 14.55 | 11.64 | 144.17 | 8.08 | 0.681 | 1 | 0.57 | 46.53 | 7 | 0 | 1 | 0 |
| 1 | 79.5 | 10.39 | 11.4 | 13.83 | 12.6 | 171.24 | 16.05 | 0.266 | 1 | 0.74 | 57.00 | 7 | 1 | 1 | 1 |
| 2 | 80 | 10.35 | 12.5 | 21.91 | 10.42 | 451.49 | 10.65 | 0.035 | 1 | 1.51 | 43.03 | 7 | 1 | 1 | 1 |
| 1 | 81 | 10.83 | 11.8 | 22.54 | 10.88 | 518.75 | 10.3 | 0.019 | 1 | 1.75 | 56.34 | 0 | 0 | 1 | 0 |
| 2 | 80 | 10.72 | 12.5 | 15.51 | 16.23 | 486.14 | 13.01 | 0.419 | 1 | 0.83 | 46.33 | 7 | 0 | 1 | 0 |
| 2 | 80 | 11.13 | 11.5 | 19.88 | 9.35 | 326.54 | 2.71 | 0.01 | 1 | 1.29 | 36.95 | 6 | 1 | 1 | 1 |
| 2 | 80 | 10.39 | 11.5 | 23.59 | 5.65 | 502.23 | 29.14 | 0.207 | 1 | 1.55 | 36.30 | 0 | 0 | 0 | 0 |
| 2 | 79 | 10.64 | 12.3 | 18.63 | 12.3 | 287.93 | 46.13 | 0.667 | 1 | 1.11 | 59.59 | 0 | 1 | 1 | 1 |
| 2 | 83 | 10.73 | 12.1 | 19.94 | 26.85 | 585.01 | 6.94 | 0.01 | 1 | 1.19 | 65.41 | 7 | 0 | 0 | 0 |
| 1 | 76 | 10.78 | 10.7 | 13.14 | 9.62 | 164.84 | 3.81 | 0.052 | 1 | 0.79 | 18.91 | 0 | 1 | 1 | 1 |
| 1 | 84 | 11.05 | 11.7 | 23.29 | 14.7 | 468.56 | 38.03 | 0.013 | 1 | 1.07 | 49.48 | 4 | 1 | 1 | 1 |
| 1 | 83 | 12.15 | 12.1 | 20.93 | 9.21 | 329.52 | 6.7 | 0.054 | 1 | 0.76 | 37.94 | 7 | 1 | 0 | 0 |
| 2 | 78 | 9.91 | 10.3 | 17.28 | 11.33 |  |  | 0.023 | 1 | 1.43 | 45.80 | 5 | 1 | 1 | 0 |
| 1 | 77 | 9.93 | 11 | 16.13 | 15.09 | 839.14 | 11.93 | 0.145 | 1 | 1.18 | 59.42 | 0 | 1 | 1 | 1 |
| 1 | 78 | 11.34 | 12.9 | 17.02 | 12.04 | 986.02 | 42.42 | 0.02 | 1 | 2.14 | 78.30 | 7 | 1 | 1 | 0 |
| 1 | 82 | 11.31 | 12.8 | 20.01 |  |  |  |  | 1 | 1.59 |  | 7 | 1 | 1 | 0 |
| 2 | 80 | 11.00 | 12.5 | 20.47 | 19.01 | 248.28 | 9.89 | 0.011 | 1 | 1.55 | 62.19 | 0 | 1 | 1 | 0 |
| 1 | 83 | 11.19 | 12.1 | 22.41 | 12.6 | 262.45 | 13.05 | 0.018 | 1 | 1.57 | 41.72 | 0 | 1 | 1 | 1 |
| 1 | 81 | 11.56 | 12.3 | 17.54 | 36.25 |  | 8.27 | 0.04 | 1 | 1.75 | 61.53 | 5 | 1 | 1 | 1 |
| 2 | 81 | 11.14 | 13 | 21.82 | 17.36 | 470.49 | 10.1 | 0.116 | 1 | 0.83 | 52.94 | 0 | 0 | 1 | 0 |
| 2 | 76 | 9.56 | 13.2 | 17.02 | 6.2 | 116.78 | 18.32 | 0.016 | 1 | 0.97 |  | 0 | 1 | 1 | 0 |
| 2 | 86 | 11.60 | 12.6 | 23.75 | 15.16 | 666.78 | 29.45 | 0.034 | 1 | 1.50 | 56.26 | 0 | 1 | 1 | 1 |
| 2 | 74 | 8.56 | 9.8 | 8.94 | 34.12 | 150.27 | 3.95 | 0.053 | 1 | 1.16 | 19.08 | 0 | 1 | 1 | 1 |
| 1 | 87 | 13.43 | 12.8 | 22.21 | 9.78 | 635.86 | 26.97 | 0.09 | 1 | 1.45 | 35.09 | 0 | 1 | 1 | 1 |
| 2 | 84 | 12.03 | 13.9 | 23.39 | 13.38 | 535.74 | 19.45 | 0.007 | 1 | 1.22 | 40.84 | 0 | 1 | 1 | 1 |
| 1 | 77.5 | 10.39 | 12.9 | 16.16 |  |  |  |  | 1 | 1.42 |  | 0 | 1 | 1 | 1 |
| 2 | 77 | 9.71 | 11.9 | 18.33 | 14.02 | 182.11 | 6.05 | 0.026 | 1 | 1.20 | 58.63 | 0 | 1 | 1 | 1 |
| 1 | 86.5 | 12.39 | 12 | 23.33 | 5.09 | 283.82 | 16.17 | 0.013 | 1 | 0.85 | 65.97 | 0 | 1 | 1 | 1 |
| 1 | 83 | 11.57 | 11.2 | 23.03 | 14.07 | 287.93 | 16 | 0.012 | 1 | 1.60 | 56.24 | 0 | 1 | 1 | 1 |
| 1 | 74.5 | 10.76 | 11.2 | 11.93 | 20.18 | 243.23 | 2.62 | 0.01 | 1 | 0.72 | 25.51 | 0 | 1 | 1 | 0 |
| 2 | 76.5 | 11.21 | 11.7 | 16.62 | 30.25 | 124.43 | 20.77 | 0.027 | 1 | 0.67 | 102.42 | 0 | 1 | 1 | 1 |
| 2 | 79 | 10.02 | 10.1 | 21.75 | 28.62 | 962.21 | 23.23 | 0.079 | 1 | 1.04 | 131.99 | 0 | 0 | 1 | 0 |
| 2 | 83 | 11.29 | 12 | 22.21 | 10.4 | 396.33 | 9.62 | 0.145 | 1 | 1.46 | 48.47 | 0 | 0 | 1 | 0 |
| 1 | 84 | 10.73 | 12.4 | 20.9 | 17.46 | 1139.89 | 11.03 | 0.048 | 1 | 1.74 | 53.64 | 0 | 1 | 1 | 0 |
| 2 | 78 | 10.09 | 12 | 17.91 | 9.67 | 408.32 | 34.21 | 0.634 | 1 | 1.04 | 39.05 | 0 | 1 | 1 | 1 |
| 1 | 84 | 12.79 | 11.3 | 18.76 | 10.09 | 228.4 | 39.34 | 0.14 | 1 |  | 61.38 | 0 | 1 | 1 | 0 |
| 2 | 85 | 11.61 | 13.1 | 19.58 | 32.79 | 765.8 | 16.06 | 0.018 | 1 | 1.05 | 65.87 | 0 | 1 | 1 | 0 |
| 2 | 81 | 10.57 | 12.2 | 22.44 | 17.21 | 440.39 | 28.53 | 0.071 | 1 | 1.04 | 29.09 | 7 | 1 | 1 | 1 |
| 1 | 74 | 10.06 | 12.3 | 12.88 |  |  |  |  | 1 |  |  | 0 | 1 | 1 | 1 |
| 1 | 84 | 12.91 | 12.2 | 19.15 | 40.56 | 677.41 | 7.18 | 0.018 | 1 |  | 51.35 | 0 | 1 | 1 | 1 |
| 2 | 80 | 9.81 | 11.6 | 21.62 | 10.22 | 516.66 | 54.46 | 0.247 | 1 | 1.04 | 77.26 | 0 | 1 | 1 | 1 |
| 1 | 77 | 8.79 | 12.3 | 13.17 | 17.27 | 230.31 | 3.37 | 0.076 | 1 | 0.96 | 23.62 | 6 | 1 | 1 | 1 |
| 2 | 79.8 | 10.01 | 13.1 | 19.71 | 23.43 | 454.68 | 17.9 | 0.027 | 1 | 1.84 | 55.00 | 7 | 1 | 1 | 1 |
| 2 | 73.5 | 10.65 | 13.1 | 11.86 |  | 1357.84 | 34.03 |  | 1 | 0.90 | 37.74 | 7 | 1 | 1 | 1 |
| 1 | 85 | 10.15 | 12.5 | 22.05 | 8.51 | 413.24 | 25.13 | 0.199 | 1 | 1.03 | 24.12 | 7 | 0 | 1 | 0 |
| 1 | 82.5 | 12.60 | 13.1 | 23.85 | 7.1 | 270.26 | 28.2 | 0.032 | 1 | 1.24 | 31.56 | 7 | 1 | 1 | 1 |
| 2 | 76 | 9.57 | 12.8 | 16.23 | 7.18 | 497.14 | 14.54 | 0.028 | 1 | 1.76 | 52.08 | 0 | 0 | 0 | 0 |
| 2 | 83.5 | 10.39 | 13.8 | 20.5 | 38.9 | 264.74 | 43.88 | 0.012 | 1 | 1.41 | 38.52 | 6 | 1 | 1 | 1 |
| 1 | 83.8 | 11.05 | 14.5 | 22.44 | 12.67 | 361.21 | 23.04 | 0.01 | 1 | 1.95 | 58.00 | 7 | 1 | 1 | 0 |
| 1 | 79.5 | 9.24 | 11.7 | 16.46 | 16.99 | 518.46 | 33.87 | 0.139 | 1 | 0.70 | 36.60 | 6 | 1 | 0 | 1 |
| 2 | 75 | 8.42 | 13.4 | 13.21 | 25.26 | 159.46 | 13.28 | 0.037 | 1 | 1.28 | 40.18 | 7 | 0 | 1 | 0 |
| 2 | 83 | 11.51 | 14.9 | 20.44 | 21.46 | 234.16 | 16.56 | 0.016 | 1 | 1.41 | 41.19 | 7 | 0 | 1 | 1 |
| 2 | 79 | 9.28 | 13.1 | 16.59 | 34.86 | 606.66 | 19.03 | 0.123 | 1 | 1.69 | 42.48 | 6 | 0 | 1 | 0 |
| 2 | 69.5 | 7.25 | 10.9 | 11.4 | 15.66 | 629.41 | 17.17 | 0.181 | 1 | 1.18 | 43.61 | 7 | 0 | 1 | 0 |
| 2 | 73 | 8.68 | 12.3 | 12.48 | 15.84 | 118.91 | 27.19 | 0.025 | 1 | 1.08 | 50.11 | 5 | 0 | 1 | 0 |
| 1 | 82 | 11.79 | 9.2 | 17.61 | 15.43 | 551.85 | 5.37 | 0.645 | 1 | 0.76 | 32.97 | 7 | 0 | 0 | 1 |
| 1 | 87 | 11.88 | 12.1 | 17.94 | 5.25 | 660.96 | 6.9 | 0.152 | 1 | 1.22 | 55.91 | 5 | 1 | 1 | 1 |
| 2 | 77 | 8.94 | 14 | 13.63 | 24.14 | 531.6 | 40.05 | 0.017 | 1 | 1.20 | 51.45 | 6 | 0 | 1 | 0 |
| 2 | 84.9 | 9.92 | 12.2 | 15.61 | 23.53 | 167.16 | 46.47 | 0.017 | 1 | 1.41 | 45.25 | 7 | 1 | 1 | 1 |
| 1 | 85 | 12.30 | 14.3 | 23.85 | 30.89 | 172.76 | 19.06 | 0.007 | 1 | 2.26 | 28.69 | 7 | 1 | 1 | 0 |
| 1 | 80 | 9.90 | 11.8 | 20.17 | 27.81 | 206.65 | 22.8 | 0.064 | 1 |  | 36.93 | 7 | 0 | 1 | 1 |
| 1 | 81 | 9.80 | 14 | 22.01 | 13.13 | 454.68 | 26.31 | 0.016 | 1 | 1.24 | 43.86 | 7 | 1 | 1 | 1 |
| 1 | 86 | 11.38 | 13.5 | 22.28 | 18.59 | 426.1 | 21.24 | 0.026 | 1 | 2.06 | 52.08 | 6 | 1 | 1 | 1 |
| 1 | 82 | 11.51 | 11.7 | 19.84 | 4.84 | 140.25 |  | 0.1 | 1 | 1.21 | 42.75 | 7 | 1 | 1 | 1 |
| 1 | 75.3 | 9.48 | 12.4 | 12.65 | 6.27 | 209.09 | 12.93 | 0.025 | 1 | 0.93 | 30.20 | 0 | 0 | 0 | 0 |
| 2 | 76.2 | 9.36 | 11.6 | 16.3 | 16.4 | 437.36 | 11.02 | 0.294 | 1 | 0.86 | 44.52 | 7 | 1 | 1 | 1 |
| 2 | 78.5 | 10.01 | 14 | 15.34 | 4.8 | 63.6 |  | 0.032 | 1 | 1.33 | 46.76 | 6 | 1 | 1 | 1 |
| 2 | 78 | 9.99 | 12.5 | 18.04 | 10.26 | 472.51 | 8.04 | 0.179 | 1 | 0.81 | 39.90 | 7 | 1 | 1 | 0 |
| 2 | 76 | 8.68 | 11.1 | 14.59 | 33.61 | 206.65 | 3.17 | 0.022 | 1 | 1.00 | 57.37 | 5 | 1 | 1 | 1 |
| 1 | 90 | 10.08 | 13.6 | 18.5 | 14.46 | 503.46 | 30.5 | 0.185 | 1 | 1.63 | 28.81 | 7 | 1 | 1 | 1 |
| 2 | 83.2 | 10.46 | 12.5 | 22.41 | 10.04 | 226.5 | 35.01 | 0.012 | 1 | 1.81 | 32.77 | 7 | 1 | 1 | 0 |
| 2 | 85.5 | 9.90 | 13.4 | 19.38 | 13.07 | 558.74 | 18.79 | 0.014 | 1 | 0.79 | 39.80 | 7 | 0 | 1 | 0 |
| 1 | 73 | 8.41 | 11.4 | 12.39 | 14.74 | 255.23 | 43.32 | 0.282 | 1 | 2.31 | 21.98 | 6 | 0 | 1 | 0 |
| 2 | 71 | 8.58 | 11.7 | 11.37 | 18.77 | 327.53 | 34.39 | 0.071 | 1 | 0.82 |  | 6 | 0 | 0 | 0 |
| 1 | 83 | 10.65 | 13.4 | 22.44 | 12.86 | 840.01 | 32.67 | 0.04 | 1 | 1.96 | 23.14 | 7 | 1 | 1 | 1 |
| 1 | 88 | 13.05 | 12.8 | 23.33 | 12.82 | 499.24 | 15.77 | 0.037 | 1 | 1.11 | 43.76 | 7 | 1 | 1 | 1 |
| 1 | 77 | 9.94 | 12.7 | 16 | 35.85 | 531.6 | 24.63 | 0.046 | 1 | 2.08 | 66.65 | 5 | 1 | 1 | 1 |
| 2 | 83 | 10.12 | 13.5 | 22.41 | 22.75 | 783.89 | 45.19 | 0.013 | 1 | 2.08 | 40.61 | 7 | 1 | 1 | 1 |
| 1 | 86 | 12.56 | 13.2 | 20.3 | 25.67 | 476.55 | 18.36 | 0.262 | 1 | 0.85 | 51.70 | 6 | 0 | 0 | 0 |
| 1 | 75.5 | 9.76 | 12.8 | 13.77 | 7.94 | 826.44 | 8.84 | 0.005 | 1 | 0.79 | 24.86 | 7 | 1 | 1 | 1 |
| 2 | 86 | 11.08 | 14.2 | 20.83 | 18.66 | 587.07 | 30.75 | 0.156 | 1 | 1.16 | 55.16 | 5 | 1 | 1 | 1 |
| 2 | 85 | 11.68 | 13.3 | 23.49 | 16.33 | 777.55 | 37.77 | 0.078 | 1 | 1.21 | 52.23 | 7 | 1 | 1 | 1 |
| 2 | 73 | 8.53 | 10.7 | 14.39 | 23.22 | 454.68 | 18.03 | 0.01 | 1 |  | 50.62 | 6 | 1 | 0 | 1 |
| 2 | 77 | 9.03 | 12 | 18.37 | 15.32 | 400.65 | 33.86 | 0.157 | 1 | 1.75 | 47.21 | 0 | 0 | 0 | 0 |
| 1 | 90 | 13.29 | 12.6 | 21.55 | 30.83 | 381.44 | 13.83 | 0.003 | 1 | 1.05 | 35.87 | 7 | 1 | 1 | 1 |
| 2 | 80 | 10.90 | 12.6 | 18.92 | 18.7 | 514.13 | 23.66 | 0.015 | 1 | 1.10 | 30.48 | 0 | 1 | 1 | 1 |
| 1 | 81 | 13.00 | 13 | 19.61 | 29.64 | 318.29 | 43.4 | 0.078 | 1 | 1.83 | 55.58 | 7 | 0 | 0 | 1 |
| 2 | 83.3 | 9.70 | 13.2 | 20.24 |  | 538.28 | 32.17 | 0.12 | 1 | 1.78 | 64.68 | 7 | 1 | 1 | 0 |
| 1 | 77 | 10.90 | 12.3 | 13.4 | 25.91 | 1260.17 | 30.67 | 0.022 | 1 | 1.08 | 52.46 | 0 | 1 | 1 | 1 |
| 1 | 82 | 10.16 | 13.3 | 19.12 | 11.01 | 826.44 | 18.28 | 0.029 | 1 | 1.90 | 33.50 | 6 | 1 | 1 | 1 |
| 1 | 82 | 10.40 | 11.4 | 17.54 | 16.46 | 333.78 | 5.46 | 0.022 | 1 | 0.63 | 25.41 | 6 | 0 | 1 | 0 |
| 1 | 81.2 | 10.80 | 11.5 | 21.29 | 33.54 | 458.59 | 17.95 | 0.062 | 1 | 0.92 | 49.89 | 7 | 1 | 1 | 1 |
| 2 | 89.5 | 12.90 | 12.9 | 15.74 | 18.47 | 324.43 | 47.95 | 0.014 | 1 | 1.26 | 35.72 | 0 | 1 | 1 | 1 |
| 2 | 77 | 9.50 | 13.8 | 12.62 | 36.73 | 472.51 | 11.29 |  | 1 | 1.53 | 28.86 | 7 | 1 | 1 | 1 |
| 1 | 80 | 10.40 | 12.4 | 19.84 | 13.28 | 188.8 | 5.36 | 0.053 | 1 | 0.88 | 48.37 | 7 | 1 | 1 | 1 |
| 2 | 80 | 8.30 | 13 | 14.52 | 10.39 | 225.23 | 16.61 | 0.01 | 1 | 1.36 | 45.88 | 7 | 1 | 1 | 1 |
| 2 | 75 | 7.60 | 12.2 | 14.95 | 18.68 | 462.54 | 19.48 | 0.035 | 1 | 0.89 | 57.73 | 6 | 0 | 1 | 0 |
| 1 | 79 | 9.40 | 11.2 | 13.17 | 5.91 | 829.81 |  | 5.403 | 1 | 0.67 | 59.16 | 7 | 1 | 1 | 1 |
| 1 | 83 | 12.50 | 13.8 | 18.99 | 29.84 | 218.95 | 20.04 | 0.019 | 1 | 1.87 | 50.92 | 0 | 0 | 1 | 1 |
| 1 | 73 | 8.60 | 11.8 | 11.33 | 5.96 | 234.16 | 8.16 | 0.016 | 1 | 0.84 | 58.61 | 6 | 1 | 1 | 1 |
| 1 | 79 | 10.00 | 12.4 | 16.66 | 13.18 | 614.16 | 9.42 | 0.048 | 1 | 1.43 | 112.55 | 7 | 1 | 1 | 1 |
| 2 | 81 | 9.00 | 13.4 | 16.36 | 18 | 180.7 | 8.48 | 0.014 | 1 | 1.42 | 46.99 | 7 | 1 | 1 | 1 |
| 2 | 80 | 9.20 | 13.2 | 21.19 | 13.7 | 416.88 | 6.86 | 0.014 | 1 | 1.58 | 70.43 | 7 | 0 | 1 | 0 |
| 2 | 74 | 8.30 | 13.7 | 11.79 | 13.21 | 220.2 | 21.26 | 0.078 | 1 | 1.40 | 67.10 | 6 | 0 | 1 | 1 |
| 2 | 71 | 7.60 | 12.4 | 11.86 | 24.31 | 390.09 | 24.28 | 0.304 | 1 | 0.92 | 30.85 | 6 | 0 | 1 | 0 |
| 1 | 77 | 9.30 | 10.8 | 13.27 | 12 | 682.82 | 75.38 | 3.061 | 1 | 1.09 | 59.87 | 5 | 1 | 1 | 1 |
| 1 | 76 | 8.40 | 10.8 | 15.05 | 21.76 | 466.51 | 20.62 | 0.27 | 1 | 1.14 | 46.31 | 2 | 1 | 1 | 1 |
| 1 | 84 | 11.35 | 12.8 | 19.06 | 16.46 | 758.84 | 11.5 | 0.193 | 1 | 1.89 | 59.24 | 5 | 0 | 1 | 0 |
| 2 | 83 | 10.40 | 12.1 | 21.62 | 11.47 | 263.37 | 6.32 | 0.211 | 1 | 1.40 | 59.92 | 7 | 1 | 1 | 0 |
| 2 | 72 | 8.52 | 11.5 | 14.59 | 24.55 | 197.04 | 4.38 | 0.067 | 1 | 1.19 | 75.98 | 6 | 0 | 1 | 0 |
| 1 | 72 | 8.80 | 9.7 | 12.39 | 10.2 | 327.53 | 2.02 | 0.11 | 1 | 1.05 | 59.04 | 6 | 0 | 0 | 0 |
| 2 | 86 | 11.10 | 12.6 | 22.77 | 16.01 | 554.14 | 20.82 | 0.185 | 1 | 1.20 | 52.84 | 6 | 1 | 0 | 0 |
| 1 | 84 | 10.60 | 13.9 | 21.55 | 11.52 | 166.05 | 5.53 | 0.005 | 1 | 1.84 | 52.05 | 6 | 1 | 1 | 0 |
| 2 | 79.4 | 8.50 | 12 | 19.71 | 30.35 | 271.65 | 46.17 | 0.013 | 1 | 2.02 | 41.11 | 7 | 1 | 1 | 1 |
| 1 | 84 | 13.10 | 12.2 | 17.84 | 10.76 | 549.57 | 6.51 | 0.046 | 1 | 1.80 | 36.68 | 6 | 1 | 1 | 0 |
| 1 | 80 | 10.40 | 12.9 | 20.3 | 26.11 | 533.82 | 28.28 | 0.168 | 1 | 1.50 | 70.00 | 7 | 1 | 1 | 1 |
| 1 | 86 | 12.20 | 14.8 | 21.85 | 13.08 | 819.74 | 24.59 | 0.036 | 1 | 1.38 | 56.92 | 6 | 1 | 1 | 1 |
| 2 | 76 | 9.60 | 13.6 | 15.28 | 16.86 | 524.99 | 18.03 | 0.025 | 1 | 1.80 | 36.20 | 7 | 1 | 1 | 1 |
| 1 | 81.2 | 9.04 | 12 | 14.69 | 18.13 | 177.28 | 7.58 | 0.006 | 1 | 1.30 | 46.63 | 6 | 0 | 1 | 0 |
| 2 | 73.9 | 8.99 | 12.4 | 14.36 | 29.88 | 284.34 | 5.87 | 0.041 | 1 | 1.14 | 27.48 | 0 | 0 | 0 | 0 |
| 1 | 89.2 | 10.76 | 12.2 | 19.19 | 34.53 | 313.73 | 13.85 | 0.004 | 1 | 1.49 | 65.89 | 7 | 1 | 1 | 1 |
| 2 | 79.6 | 8.79 | 12.3 | 17.18 | 13.55 | 93.03 | 7.11 | 0.011 | 1 | 1.44 | 35.52 | 7 | 1 | 1 | 1 |
| 1 | 77.9 | 9.45 | 12.6 | 11.27 | 35.65 | 354.63 | 29.44 | 0.066 | 1 | 1.64 | 53.01 | 6 | 1 | 1 | 1 |
| 1 | 81.2 | 11.64 | 13.6 | 17.54 | 9.05 | 348.13 | 10.85 | 0.02 | 1 | 1.50 | 42.55 | 6 | 1 | 1 | 1 |
| 1 | 80.8 | 10.35 | 12.5 | 14.62 | 13.78 | 456.63 | 19.65 | 0.08 | 1 | 1.35 | 50.67 | 7 | 1 | 1 | 0 |
| 2 | 84.5 | 11.02 | 12.2 | 20.73 | 17.77 | 439.26 | 25.61 | 0.18 | 1 | 1.32 | 76.28 | 0 | 1 | 1 | 1 |
| 2 | 87.4 | 10.87 | 12.8 | 17.28 | 29.6 | 346.51 | 24.54 | 0.198 | 1 | 1.09 | 91.45 | 7 | 1 | 1 | 1 |
| 2 | 82.1 | 9.53 | 13.5 | 20.83 | 16.87 | 516.29 | 28.18 | 0.023 | 1 | 1.29 | 93.72 | 7 | 1 | 1 | 1 |
| 1 | 78.9 | 9.65 | 11.5 | 12.22 | 20.51 | 253.88 | 2.27 | 0.01 | 1 | 0.94 | 52.81 | 0 | 0 | 1 | 0 |
| 2 | 79.4 | 9.99 | 13.1 | 22.24 | 25.23 | 127.92 | 18.86 | 0.04 | 1 | 0.98 | 54.65 | 7 | 1 | 1 | 1 |
| 2 | 74.8 | 8.93 | 12.8 | 17.45 | 17.73 | 238.04 | 12.28 | 0.19 | 1 |  | 60.80 | 6 | 0 | 1 | 0 |
| 1 | 84.6 | 11.25 | 11 | 19.32 | 29.43 | 492.97 | 44.75 | 0.01 | 1 | 1.26 | 32.49 | 7 | 1 | 1 | 1 |
| 2 | 77 | 11.15 | 12.8 | 18.63 | 17.9 | 348.13 | 12.9 | 0.026 | 1 | 1.62 | 79.28 | 7 | 1 | 1 | 1 |
| 1 | 76.9 | 9.49 | 13 | 15.38 | 15.51 | 376.31 | 7.44 | 0.007 | 1 | 0.88 | 45.27 | 7 | 1 | 1 | 1 |
| 2 | 77.1 | 9.89 | 12.2 | 14.16 | 16.28 | 1067.04 | 23.77 | 0.183 | 1 | 1.27 | 40.53 | 7 | 0 | 1 | 0 |
| 1 | 86.7 | 12.45 | 12.3 | 23.43 | 9.4 | 278.66 | 10.84 | 0.015 | 1 | 1.89 | 94.96 | 7 | 1 | 1 | 1 |
| 1 | 88.5 | 10.77 | 12 | 23.49 | 11.73 | 371.23 | 16.36 | 0.008 | 1 | 1.60 | 52.16 | 0 | 1 | 1 | 1 |
| 2 | 77.1 | 9.18 | 11.8 | 20.24 | 12.67 | 332.21 | 19.56 | 0.039 | 1 | 0.82 | 47.11 | 7 | 1 | 1 | 1 |
| 1 | 84.9 | 11.59 | 11 | 22.6 | 9.64 | 332.21 | 8.87 | 0.017 | 1 | 1.39 | 48.20 | 0 | 1 | 1 | 1 |
| 2 | 84.1 | 12.39 | 12.7 | 22.05 | 4.03 | 422.4 | 18.4 | 0.024 | 1 | 1.51 | 46.43 | 7 | 1 | 1 | 1 |
| 2 | 81.7 | 10.65 | 13.7 | 21.72 | 17.39 | 871.43 | 16.24 | 0.015 | 1 |  | 32.77 | 7 | 1 | 1 | 1 |
| 1 | 75.3 | 8.67 | 12.9 | 13.57 | 29.64 | 439.26 | 16.23 | 0.017 | 1 | 1.42 | 70.96 | 7 | 1 | 1 | 1 |
| 1 | 80.6 | 10.65 | 12.9 | 15.8 | 24.07 | 752.7 | 36.06 | 0.05 | 1 | 1.38 | 30.20 | 7 | 0 | 1 | 0 |
| 2 | 75 | 8.51 | 13.5 | 12.88 | 31.27 | 520.63 | 26.83 | 0.036 | 1 | 1.15 | 54.07 | 6 | 1 | 1 | 1 |
| 2 | 79.6 | 10.22 | 12.9 | 14.39 | 30.79 | 486.76 | 19.65 | 0.014 | 1 | 1.33 | 51.15 | 6 | 0 | 1 | 0 |
| 1 | 85.1 | 11.75 | 13.4 | 23.52 | 21.46 | 728.65 | 27.38 | 0.025 | 1 | 1.73 | 88.63 | 7 | 1 | 1 | 0 |
| 1 | 81.5 | 10.97 | 13 | 18.96 | 17.92 | 470.5 | 29.89 | 0.005 | 1 | 0.76 | 44.72 | 6 | 1 | 1 | 1 |
| 2 | 84 | 11.00 | 13.2 | 21.85 | 26.79 | 390.09 | 31.92 | 0.195 | 1 | 1.18 | 70.46 | 7 | 1 | 1 | 1 |
| 1 | 78 | 9.20 | 13.2 | 11.76 | 21.83 | 596.8 | 18.63 | 0.03 | 1 | 1.10 | 47.90 | 5 | 1 | 1 | 1 |
| 1 | 79.5 | 9.50 | 12 | 19.52 | 6.81 | 416.88 | 20.53 | 0.037 | 1 | 0.97 | 42.85 | 0 | 0 | 1 | 0 |
| 2 | 79 | 9.00 | 14.2 | 17.91 | 30.79 | 705.37 | 23.87 | 0.008 | 1 | 1.54 | 55.08 | 6 | 1 | 1 | 0 |
| 2 | 73 | 8.40 | 13.1 | 13.47 | 11.03 | 616.68 | 13.51 | 0.008 | 1 | 1.12 | 53.01 | 7 | 0 | 0 | 0 |
| 1 | 80.5 | 9.98 | 13.7 | 19.42 | 9.83 | 685.6 | 22.02 | 0.046 | 1 | 1.57 | 53.44 | 7 | 1 | 1 | 1 |
| 2 | 81.5 | 8.65 | 12.2 | 19.81 | 14.76 | 930.49 | 39.5 | 0.013 | 1 | 1.05 | 52.21 | 7 | 1 | 1 | 1 |
| 2 | 78 | 9.71 | 12.3 | 21.13 | 18.5 | 448.85 | 16.93 | 0.218 | 1 | 1.17 | 69.88 | 7 | 0 | 1 | 1 |
| 1 | 83 | 11.43 | 13.1 | 20.24 | 11.22 | 582.27 | 12.19 | 0.046 | 1 | 1.66 | 76.03 | 7 | 1 | 1 | 1 |
| 2 | 83 | 8.65 | 14.1 | 22.41 | 17.77 | 614.16 | 16.03 | 0.04 | 1 | 1.00 | 51.37 | 7 | 1 | 1 | 1 |
| 1 | 86 | 11.07 | 12.9 | 21.65 | 6.93 | 406.01 | 14.36 | 0.029 | 1 | 1.99 | 70.58 | 7 | 1 | 1 | 1 |
| 1 | 90 | 12.58 | 13.7 | 18.86 | 11.12 | 398.88 | 35.9 | 0.011 | 1 | 1.37 | 77.99 | 7 | 1 | 1 | 1 |
| 2 | 82 | 9.52 | 12 | 18.37 | 13.01 | 647.64 | 14.57 | 0.113 | 1 | 1.27 | 37.28 | 7 | 1 | 1 | 1 |
| 2 | 82 | 10.78 | 11.1 | 22.87 | 16.59 | 260.64 | 25.68 | 0.008 | 1 | 1.99 | 54.70 | 7 | 1 | 1 | 1 |
| 1 | 81 | 11.03 | 11.3 | 17.05 | 14 | 734.59 | 11.31 | 0.025 | 1 | 1.59 | 45.53 | 7 | 0 | 1 | 1 |
| 1 | 78 | 9.93 | 12.4 | 13.86 | 6.99 | 476.55 | 9.12 | 0.021 | 1 | 1.60 | 48.12 | 7 | 1 | 0 | 1 |
| 1 | 78 | 9.50 | 12.9 | 16.89 | 18.65 | 330.65 | 32.62 | 0.018 | 1 | 1.36 | 43.53 | 7 | 0 | 1 | 0 |
| 1 | 79 | 9.00 | 11.5 | 17.71 | 5.56 | 87.51 | 11.1 | 0.403 | 1 | 1.02 | 36.02 | 7 | 0 | 0 | 0 |
| 2 | 85 | 11.20 | 13 | 21.72 | 12 | 533.82 | 14.11 | 0.014 | 1 | 0.95 | 83.44 | 7 | 1 | 1 | 1 |
| 1 | 84 | 12.70 | 11.8 | 20.24 | 11.4 | 374.61 | 62.53 | 0.171 | 1 | 0.98 | 63.75 | 7 | 0 | 0 | 1 |
| 1 | 82 | 9.16 | 11.9 | 18.17 | 16.76 | 1076.04 | 17.79 | 0.023 | 1 | 1.35 | 44.34 | 7 | 1 | 1 | 1 |
| 1 | 81 | 8.32 | 9.7 | 12.19 | 10.44 | 549.57 |  | 0.016 | 1 | 1.36 | 65.69 | 7 | 1 | 1 | 0 |
| 2 | 85 | 11.12 | 12.6 | 23.92 | 13.07 | 354.63 | 11.21 | 0.018 | 1 | 1.52 | 30.60 | 0 | 1 | 1 | 1 |
| 2 | 78 | 10.22 | 12.6 | 19.22 | 17.34 | 448.85 | 15.51 | 0.011 | 1 | 1.33 | 87.52 | 7 | 1 | 1 | 1 |
| 1 | 81 | 9.82 | 13.1 | 17.81 | 17.92 | 893.08 | 19.08 | 0.021 | 1 | 1.44 | 70.73 | 7 | 0 | 1 | 0 |
| 1 | 79 | 8.50 | 11.6 | 13.01 | 13.38 | 335.36 | 10.8 | 0.122 | 1 | 1.37 | 78.80 | 6 | 1 | 1 | 1 |
| 2 | 74 | 8.30 | 12.3 | 13.27 | 35.2 | 439.26 | 35.3 | 0.105 | 1 | 1.84 | 73.71 | 7 | 0 | 1 | 1 |
| 2 | 77 | 10.32 | 12.5 | 13.37 | 11.32 | 371.23 | 24.02 | 0.137 | 1 | 1.48 | 68.57 | 5 | 0 | 1 | 1 |
| 2 | 74 | 7.81 | 11.2 | 11.63 | 35.44 | 372.92 | 24.01 | 0.296 | 1 | 1.36 | 78.30 | 4 | 1 | 1 | 1 |
| 2 | 80 | 9.99 | 12.3 | 20.4 | 11.47 | 287.2 | 9.72 | 0.278 | 1 | 1.26 | 58.66 | 7 | 0 | 0 | 0 |
| 2 | 74 | 7.66 | 12.1 | 13.4 | 31.88 | 427.96 | 17.37 | 0.266 | 1 | 0.90 | 80.77 | 6 | 0 | 0 | 1 |
| 1 | 75 | 8.90 | 9.8 | 14.46 | 7.64 | 691.19 |  | 0.625 | 1 | 0.84 | 60.98 | 5 | 1 | 1 | 1 |
| 2 | 76 | 9.52 | 12.5 | 16.46 | 8.78 | 164.95 | 4.09 | 0.005 | 1 | 1.35 | 101.97 | 7 | 0 | 1 | 0 |
| 1 | 79 | 10.21 | 11.1 | 18.63 | 11.2 | 1122.39 | 8.9 | 0.813 | 1 | 1.01 | 38.67 | 7 | 1 | 1 | 1 |
| 1 | 81 | 10.85 | 13.3 | 16.99 | 16.26 | 596.8 | 53.52 | 0.293 | 1 | 1.10 | 47.74 | 6 | 1 | 1 | 0 |
| 1 | 80 | 10.05 | 13.3 | 21.39 | 12.66 | 200.62 | 8.38 | 0.049 | 1 | 1.48 | 44.59 | 7 | 1 | 1 | 0 |
| 1 | 81 | 10.82 | 12.9 | 16.92 | 4.53 | 529.39 | 3.37 | 0.087 | 1 | 0.84 | 71.67 | 7 | 1 | 1 | 1 |
| 2 | 76 | 10.32 | 14 | 13.4 | 4.94 | 809.8 | 17.46 | 0.159 | 1 | 1.23 | 36.75 | 6 | 1 | 1 | 1 |
| 2 | 85 | 10.82 | 13.3 | 18.63 | 4.92 | 327.53 | 10.16 | 0.056 | 1 | 1.26 | 53.74 | 6 | 1 | 1 | 1 |
| 2 | 89 | 12.63 | 12.8 | 23.82 | 11.6 | 306.22 | 20.71 | 0.095 | 1 | 1.59 | 65.34 | 7 | 1 | 1 | 1 |
| 1 | 83 | 10.72 | 12.8 | 14.65 | 13.19 | 187.63 | 4.97 | 0.016 | 1 | 1.36 | 38.04 | 7 | 0 | 1 | 0 |
| 1 | 85 | 11.99 | 13.5 | 23.43 | 12.88 | 867.88 | 4.48 | 0.232 | 1 | 0.95 | 58.56 | 6 | 1 | 1 | 1 |
| 1 | 82.2 | 11.82 | 12.7 | 18.23 |  |  |  |  | 1 |  |  | 7 | 1 | 1 | 1 |
| 1 | 69.1 | 7.71 | 12.1 | 7.92 |  |  |  |  | 1 |  |  | 7 | 0 | 0 | 0 |
| 2 | 91.3 | 14.94 | 11.5 | 19.12 | 7.89 | 198.74 | 9.44 | 0.011 | 1 | 1.27 | 35.52 | 7 | 0 | 1 | 0 |
| 1 | 82 | 11.16 | 13.4 | 20.11 | 11.15 | 305.24 |  |  | 1 | 1.13 | 70.03 | 7 | 0 | 0 | 0 |
| 2 | 86.8 | 12.14 | 12 | 23.62 | 16.09 | 1400.09 | 37.72 | 0.051 | 1 | 1.12 | 45.12 | 7 | 1 | 1 | 1 |
| 1 | 79.4 | 9.74 | 11.6 | 14.92 |  |  |  |  | 1 |  |  | 7 | 1 | 1 | 1 |
| 1 | 70 | 6.92 | 10.8 | 6.24 |  |  |  |  | 1 |  |  | 0 | 0 | 1 | 0 |
| 2 | 77 | 9.22 | 12 | 15.44 |  |  |  |  | 1 |  |  | 0 | 0 | 0 | 0 |
| 2 | 81.3 | 9.18 | 11.6 | 20.96 | 11.66 | 261.99 | 38.56 | 0.01 | 1 | 1.61 | 47.54 | 7 | 1 | 1 | 0 |
| 1 | 74.6 | 10.25 | 11.4 | 10.22 |  |  |  |  | 1 |  |  | 5 | 1 | 1 | 1 |
| 1 | 84 | 11.43 | 11.4 | 21.45 | 19.11 | 872.59 | 35.07 | 0.02 | 1 | 1.16 | 83.61 | 6 | 0 | 1 | 0 |
| 1 | 71.5 | 8.59 | 13 | 10.41 |  |  |  |  | 1 |  |  | 7 | 1 | 1 | 0 |
| 1 | 72 | 9.34 | 10 | 11.24 |  |  |  |  | 1 |  |  | 7 | 1 | 1 | 0 |
| 1 | 79.3 | 9.93 | 11.2 | 20.11 |  |  |  |  | 1 | 1.11 |  | 7 | 1 | 1 | 0 |
| 2 | 67.6 | 7.46 | 13.1 | 7.89 |  |  |  |  | 1 |  |  | 0 | 0 | 0 | 0 |
| 2 | 86.3 | 11.59 | 11.2 | 23.89 | 10.23 | 202.22 | 9.58 | 0.112 | 1 | 0.92 | 24.17 | 7 | 1 | 1 | 0 |
| 1 | 67.6 | 8.31 | 10.5 | 6.51 |  |  |  |  | 1 |  |  | 0 | 0 | 1 | 0 |
| 1 | 84.8 | 11.43 | 12.2 | 19.65 | 6.9 | 293.7 | 16.58 | 0.008 | 1 | 1.09 | 31.99 | 7 | 0 | 0 | 1 |
| 2 | 82 | 11.61 | 12.7 | 20.96 | 7.06 | 499.77 | 26.35 | 0.002 | 1 | 1.54 | 45.73 | 7 | 0 | 1 | 0 |
| 2 | 71.4 | 9.52 | 11.9 | 10.05 |  |  |  |  | 1 |  |  | 7 | 0 | 1 | 0 |
| 2 | 89.1 | 10.24 | 11.8 | 21.55 | 7.07 | 385.92 | 12.02 | 0.208 | 1 | 0.82 | 23.32 | 4 | 0 | 1 | 1 |
| 2 | 83 | 10.82 | 11.6 | 23.39 | 11.79 | 369.07 | 28.4 | 0.021 | 1 | 0.70 | 37.89 | 7 | 1 | 1 | 1 |
| 1 | 84.3 | 10.76 | 12.1 | 23.36 | 18.2 | 1091.5 | 16.98 | 0.02 | 1 | 0.78 | 40.01 | 7 | 1 | 1 | 1 |
| 1 | 80.1 | 10.82 | 11.8 | 19.12 |  |  |  |  | 1 | 1.14 |  | 7 | 0 | 1 | 0 |
| 2 | 78 | 9.83 | 12.3 | 19.78 | 11.35 | 727.78 | 12.19 | 0.021 | 1 | 1.46 | 59.49 | 7 | 1 | 1 | 1 |
| 2 | 87.5 | 10.09 | 11.1 | 15.67 | 23.18 | 104.55 | 4.06 | 0.082 | 1 | 0.70 | 53.42 | 7 | 0 | 0 | 0 |
| 2 | 83.2 | 11.99 | 12.6 | 23.13 | 13.58 | 602.53 | 48.39 | 0.007 | 1 | 1.08 | 43.08 | 6 | 1 | 1 | 0 |
| 1 | 83.3 | 12.00 | 12.8 | 21.62 | 9.16 | 1019.26 | 39.31 | 1.121 | 1 | 0.78 | 58.71 | 7 | 0 | 0 | 1 |
| 2 | 81.1 | 10.24 | 12.4 | 18.4 | 10.79 | 585.39 | 9.31 | 0.026 | 1 | 1.51 | 53.67 | 6 | 0 | 1 | 0 |
| 1 | 82 | 11.22 | 12.4 | 20.14 | 7.03 | 578.18 | 28.37 | 0.159 | 1 | 0.93 | 47.87 | 7 | 0 | 1 | 0 |
| 1 | 68.8 | 8.67 | 11.6 | 9.17 |  |  |  |  | 1 |  |  | 7 | 1 | 1 | 1 |
| 2 | 84 | 9.80 | 13.1 | 11.6 | 12.39 | 1466.65 | 15.55 |  | 1 | 1.48 | 50.64 | 6 | 0 | 1 | 1 |
| 1 | 84 | 11.58 | 11.3 | 16.16 | 14.57 | 1393.65 | 18.25 | 0.017 | 1 | 1.08 | 41.77 | 6 | 1 | 1 | 0 |
| 2 | 78.8 | 10.03 | 13.4 | 20.4 | 26.28 |  | 26.07 | 0.038 | 1 | 1.10 | 49.94 | 2 | 0 | 1 | 0 |
| 2 | 82.7 | 10.56 | 13.9 | 20.44 | 21.42 |  | 31.84 | 0.34 | 1 | 1.06 | 56.16 | 2 | 0 | 1 | 0 |
| 2 | 83.8 | 10.60 | 11.7 | 22.44 | 16.55 | 707.18 | 18.51 | 0.072 | 1 | 1.12 |  | 7 | 1 | 1 | 1 |
| 2 | 74.5 | 8.88 | 12.3 | 14.06 | 20.43 | 780.44 | 14.83 | 0.012 | 1 | 1.36 | 61.00 | 7 | 0 | 0 | 1 |
| 1 | 86.5 | 11.73 | 11.9 | 20.34 | 5.48 | 479.13 | 8.02 | 0.038 | 1 | 1.90 | 50.72 | 4 | 1 | 1 | 1 |
| 1 | 90.2 | 13.31 | 13.3 | 23.95 | 5.19 | 293.7 | 25.04 | 0.012 | 1 | 0.92 | 35.42 | 6 | 0 | 1 | 1 |
| 2 | 82 | 10.94 | 12.7 | 19.88 | 6.57 | 379.12 | 8.69 | 0.027 | 1 | 1.32 | 44.74 | 6 | 1 | 1 | 1 |
| 1 | 88 | 11.60 | 12.7 | 23.72 |  |  |  |  | 1 | 1.05 |  | 7 | 0 | 1 | 0 |
| 1 | 79 | 10.03 | 11.1 | 17.05 |  |  |  |  | 1 | 0.90 |  | 6 | 0 | 1 | 0 |
| 1 | 84.4 | 10.70 | 12.6 | 19.29 | 8.34 | 648.81 | 28.52 | 0.027 | 1 | 1.49 | 24.05 | 7 | 0 | 1 | 0 |
| 2 | 70.2 | 8.00 | 11.8 | 13.11 |  |  |  |  | 1 |  |  | 7 | 0 | 1 | 0 |
| 1 | 73 | 10.46 | 12.5 | 10.55 |  |  |  |  | 1 |  |  | 7 | 0 | 0 | 0 |
| 1 | 82.3 | 10.67 | 11.4 | 23.89 | 7.46 | 281.03 | 13.59 | 0.058 | 1 | 1.23 | 53.67 | 7 | 1 | 1 | 1 |
| 1 | 81.4 | 10.09 | 12.9 | 21.98 | 15.02 | 793.41 | 46.63 | 0.143 | 1 | 1.45 | 48.75 | 6 | 0 | 1 | 0 |
| 2 | 79 | 10.48 | 12.5 | 19.09 | 16.05 | 710.09 | 20.54 | 0.007 | 1 | 1.63 | 59.24 | 7 | 1 | 1 | 0 |
| 2 | 80.6 | 11.26 | 12.6 | 18.4 | 11.62 | 171.92 | 4.87 | 0.135 | 1 | 1.15 | 55.89 | 7 | 0 | 1 | 1 |
| 2 | 78.1 | 10.67 | 12.2 | 14.09 | 10.37 | 329.19 | 9.28 | 0.033 | 1 | 0.90 | 53.95 | 7 | 0 | 0 | 1 |
| 2 | 80 | 10.78 | 11.2 | 17.91 | 11.6 | 767.7 | 43.32 | 0.259 | 1 | 0.96 | 51.88 | 7 | 1 | 1 | 1 |
| 2 | 71.6 | 9.47 | 11.8 | 9.07 |  |  |  |  | 1 |  |  | 7 | 0 | 0 | 0 |
| 2 | 82 | 10.75 | 13.2 | 16.13 | 8.95 | 739.83 | 12.31 | 0.044 | 1 | 1.60 | 80.67 | 7 | 1 | 1 | 1 |
| 2 | 71.6 | 9.88 | 10.4 | 11.83 |  |  |  |  | 1 |  |  | 7 | 0 | 0 | 1 |
| 1 | 76.7 | 10.21 | 10.7 | 7.66 | 31.88 | 59.56 | 7.6 | 0.01 | 1 | 1.21 | 61.23 | 7 | 0 | 1 | 0 |
| 1 | 65.4 | 7.98 | 12.3 | 6.34 |  |  |  |  | 1 |  |  | 0 | 0 | 0 | 0 |
| 2 | 74.3 | 8.13 | 12.1 | 14.29 | 17.92 | 793.41 | 26.93 | 0.044 | 1 | 1.58 |  | 7 | 1 | 1 | 1 |
| 1 | 81.4 | 10.06 | 11.7 | 18.99 | 18.96 | 529.93 | 4.83 | 0.005 | 1 | 1.43 | 79.10 | 6 | 1 | 1 | 1 |
| 1 | 82.5 | 11.87 | 11.3 | 17.12 | 4.3 | 106.37 | 6.1 |  | 1 | 0.82 | 57.78 | 7 | 0 | 0 | 1 |
| 1 | 79.3 | 10.01 | 12 | 18.83 | 14.1 | 318.57 | 4.55 | 0.143 | 1 | 1.12 | 33.80 | 0 | 1 | 1 | 0 |
| 2 | 63.9 | 7.50 | 11.7 | 6.24 |  |  |  |  | 1 |  |  | 7 | 0 | 0 | 1 |
| 1 | 77.2 | 9.68 | 13 | 17.77 | 15.19 | 813.28 | 22.01 | 0.138 | 1 | 1.19 | 57.60 | 7 | 1 | 1 | 1 |
| 2 | 74.4 | 9.96 | 12.6 | 10.48 |  |  |  |  | 1 |  |  | 5 | 0 | 1 | 0 |
| 1 | 88.8 | 12.26 | 12 | 22.77 | 15.36 | 793.41 | 49.2 | 0.014 | 1 | 1.67 | 46.74 | 6 | 1 | 1 | 1 |
| 1 | 69.3 | 9.34 | 12.1 | 10.35 |  |  |  |  | 1 |  |  | 7 | 1 | 1 | 1 |
| 2 | 79.2 | 11.60 | 13.2 | 15.8 | 15.77 | 1154.81 | 33.95 | 0.127 | 1 | 1.00 | 48.70 | 7 | 1 | 1 | 1 |
| 1 | 70.6 | 7.65 | 10.7 | 8.71 |  |  |  |  | 1 |  |  | 6 | 0 | 1 | 1 |
| 1 | 73 | 9.05 | 12.1 | 9.92 |  |  |  |  | 1 |  |  | 5 | 1 | 1 | 0 |
| 1 | 79.1 | 10.51 | 12.2 | 19.32 | 16 | 809.93 | 50.58 | 0.077 | 1 | 1.05 | 63.93 | 6 | 1 | 1 | 1 |
| 1 | 81.4 | 10.80 | 11.5 | 20.14 |  |  |  |  | 1 | 1.37 |  | 7 | 0 | 1 | 0 |
| 1 | 72.8 | 9.59 | 10.1 | 11.96 |  |  |  |  | 1 |  |  | 7 | 0 | 1 | 1 |
| 2 | 80.7 | 9.39 | 13.1 | 23.33 | 9.1 | 501.87 | 18.53 | 0.179 | 1 | 0.83 | 38.57 | 7 | 0 | 1 | 0 |
| 1 | 85.4 | 13.57 | 12.4 | 23.75 | 11.6 | 659.55 | 37.4 | 0.04 | 1 | 1.60 | 56.94 | 7 | 1 | 1 | 1 |
| 1 | 77.4 | 11.99 | 11.4 | 16.99 |  |  |  |  | 1 | 1.13 |  | 7 | 1 | 1 | 1 |
| 1 | 81.2 | 10.48 | 10 | 19.61 | 11.13 | 159.15 | 6.51 | 0.102 | 1 | 0.88 | 42.50 | 7 | 0 | 0 | 0 |
| 2 | 81.7 | 9.62 | 11.3 | 23.56 | 10.75 | 495.58 | 7.22 | 0.01 | 1 | 0.98 | 53.72 | 7 | 0 | 0 | 1 |
| 1 | 71.7 | 8.31 | 12.2 | 8.71 |  |  |  |  | 1 |  |  | 5 | 0 | 0 | 0 |
| 1 | 67.5 | 8.60 | 12.8 | 7.79 |  |  |  |  | 1 |  |  | 1 | 0 | 1 | 0 |
| 2 | 79.6 | 10.23 | 12.5 | 20.21 |  |  |  |  | 1 |  |  | 0 | 0 | 0 | 0 |
| 1 | 82.1 | 12.85 | 13.5 | 20.07 | 13.68 | 710.09 | 35.1 | 0.034 | 1 | 1.77 | 57.85 | 7 | 1 | 1 | 1 |
| 1 | 79.3 | 9.81 | 12.3 | 20.67 | 11.32 | 94.71 | 2.44 | 0.011 | 1 | 0.68 | 69.65 | 4 | 0 | 1 | 0 |
| 1 | 68.6 | 8.95 | 11.7 | 7.52 |  |  |  |  | 1 |  |  | 4 | 0 | 1 | 0 |
| 1 | 84.1 | 11.99 | 11.7 | 17.91 | 14.29 | 587.81 | 24.22 | 0.133 | 1 | 1.43 | 40.36 | 7 | 1 | 1 | 1 |
| 2 | 79.2 | 10.07 | 11.8 | 17.64 | 6.92 |  | 7.86 |  | 1 |  | 42.10 | 7 | 0 | 1 | 0 |
| 2 | 75.6 | 10.90 | 12.5 | 11.14 |  |  |  |  | 1 |  |  | 7 | 0 | 1 | 0 |
| 1 | 74.3 | 9.32 | 11.6 | 10.15 |  |  |  |  | 1 |  |  | 7 | 0 | 1 | 0 |
| 2 | 76.6 | 9.35 | 13 | 16.16 | 16.84 | 692.83 | 20.97 | 0.032 | 1 | 1.23 | 48.53 | 7 | 0 | 1 | 0 |
| 1 | 67.5 | 8.88 | 11.1 | 6.87 |  |  |  |  | 1 |  |  | 7 | 0 | 1 | 1 |
| 2 | 80.3 | 10.31 | 13.5 | 20.57 |  |  |  |  | 1 | 0.92 |  | 5 | 1 | 1 | 1 |
| 2 | 78.3 | 10.86 | 11.5 | 17.97 | 15.14 | 554.74 | 20.89 | 0.05 | 1 | 2.23 | 71.09 | 7 | 1 | 1 | 1 |
| 2 | 80.1 | 10.95 | 12.1 | 19.25 | 9.42 | 541.08 | 9.83 | 0.052 | 1 | 0.93 | 52.68 | 7 | 0 | 1 | 1 |
| 1 | 82 | 11.87 | 11.8 | 19.19 |  |  |  |  | 1 |  |  | 7 | 1 | 1 | 1 |
| 1 | 79.6 | 11.08 | 12.6 | 19.52 |  |  |  |  | 1 |  |  | 7 | 0 | 1 | 0 |
| 1 | 65.2 | 8.44 | 9.3 | 6.41 |  |  |  |  | 1 |  |  | 4 | 0 | 0 | 0 |
| 1 | 78.1 | 10.05 | 12.9 | 18.79 |  |  |  |  | 1 |  |  | 7 | 0 | 1 | 1 |
| 1 | 86.2 | 12.72 | 12.3 | 22.24 | 9.31 | 612.54 | 29.09 | 0.433 | 1 | 0.96 | 43.11 | 7 | 1 | 1 | 1 |
| 1 | 82.4 | 9.68 | 12.8 | 22.74 | 15.22 | 610.02 | 22.33 | 0.007 | 1 | 1.29 | 125.79 | 5 | 1 | 1 | 1 |
| 1 | 71.8 | 10.85 | 11.5 | 8.08 |  |  |  |  | 1 |  |  | 7 | 0 | 0 | 0 |
| 1 | 78.5 | 10.89 | 12 | 14.95 |  |  |  |  | 1 |  |  | 7 | 1 | 1 | 0 |
| 2 | 82.5 | 10.97 | 12.2 | 23.49 |  |  |  |  | 1 |  |  | 7 | 1 | 1 | 1 |
| 1 | 75.5 | 9.64 | 11.1 | 11.56 |  |  |  |  | 1 |  |  | 7 | 1 | 1 | 1 |
| 1 | 79 | 11.94 | 11.1 | 12.71 |  |  |  |  | 1 |  |  | 7 | 1 | 1 | 1 |
| 1 | 79.2 | 10.45 | 11.1 | 15.51 |  | 796.68 | 35.45 | 0.073 | 1 | 1.35 | 48.50 | 7 | 0 | 0 | 0 |
| 2 | 82.2 | 10.05 | 11.3 | 19.68 | 10.19 | 194.15 | 14.9 | 0.023 | 1 | 0.89 | 77.59 | 7 | 0 | 1 | 0 |
| 2 | 81.6 | 10.59 | 12 | 22.34 | 5.01 | 465.11 | 14.16 | 0.093 | 1 | 0.75 | 100.88 | 6 | 1 | 1 | 0 |
| 2 | 67.5 | 7.75 | 10.8 | 6.51 |  |  |  |  | 1 |  |  | 7 | 1 | 0 | 0 |
| 1 | 75 | 10.51 | 11.5 | 10.87 |  |  |  |  | 1 |  |  | 5 | 0 | 1 | 0 |
| 1 | 76.3 | 10.12 | 10.8 | 14.88 | 10.13 | 156.02 | 3.55 | 0.183 | 1 |  | 52.91 | 5 | 0 | 0 | 0 |
| 1 | 74.5 | 9.97 | 11.5 | 10.58 |  |  |  |  | 1 |  |  | 6 | 1 | 1 | 1 |
| 2 | 74.7 | 9.91 | 11.9 | 12.02 |  |  |  |  | 1 |  |  | 6 | 0 | 0 | 0 |
| 2 | 70.4 | 9.93 | 9.7 | 9.23 |  |  |  |  | 1 |  |  | 7 | 0 | 1 | 0 |
| 2 | 79 | 9.82 | 11.5 | 16.43 | 28.18 | 568.7 | 6.71 | 0.017 | 1 | 1.40 | 79.23 | 7 | 1 | 1 | 0 |
| 1 | 77.6 | 10.96 | 11.3 | 15.61 | 14.19 | 940.68 | 44.95 | 0.992 | 1 | 0.66 | 80.92 | 7 | 1 | 1 | 0 |
| 1 | 81.2 | 11.36 | 11.7 | 16.62 | 16.44 | 890.93 | 72.51 | 0.01 | 1 | 2.03 | 78.60 | 6 | 1 | 1 | 0 |
| 2 | 75.5 | 8.27 | 11.3 | 16.13 | 13.67 | 126.99 |  | 0.013 | 1 | 0.57 | 92.26 | 7 | 0 | 0 | 0 |
| 2 | 76.1 | 9.56 | 11.6 | 18.17 |  |  |  |  | 1 | 0.68 | 53.82 | 5 | 1 | 1 | 1 |
| 1 | 80.4 | 10.51 | 12.1 | 19.98 | 14.97 | 1393.65 | 75.09 | 0.034 | 1 | 1.71 | 79.23 | 7 | 1 | 1 | 1 |
| 2 | 82.5 | 11.44 | 12.4 | 19.98 | 18.04 | 408.68 | 34.45 | 0.033 | 1 | 1.70 | 80.92 | 5 | 1 | 1 | 1 |
| 1 | 85.8 | 11.62 | 13.6 | 18.07 | 17.69 | 1446.25 | 27.68 | 0.144 | 1 | 1.70 | 55.13 | 7 | 1 | 1 | 0 |
| 1 | 77.3 | 10.96 | 11.2 | 14.62 | 14.43 | 364.11 | 21.94 | 0.047 | 1 | 1.40 | 78.60 | 7 | 1 | 1 | 0 |
| 2 | 84.5 | 11.19 | 12.3 | 21.32 | 15.23 | 887.23 | 6.66 | 0.092 | 1 | 1.27 | 92.26 | 7 | 1 | 1 | 1 |
| 2 | 77.1 | 9.75 | 12.4 | 14.75 | 12.04 | 508.23 | 13.33 | 0.04 | 1 | 1.13 | 44.87 | 7 | 0 | 1 | 0 |
| 1 | 77.2 | 9.11 | 11.9 | 15.31 | 31.02 | 713.01 | 25.48 | 0.179 | 1 | 1.37 | 62.16 | 6 | 0 | 1 | 0 |
| 1 | 74.2 | 9.42 | 11.1 | 12.42 |  |  |  |  | 1 |  |  | 4 | 0 | 1 | 0 |
| 1 | 80.3 | 11.15 | 11.8 | 18.69 | 7.71 | 483.2 | 8.62 | 0.017 | 1 | 1.10 | 68.16 | 5 | 1 | 1 | 0 |
| 2 | 76.7 | 9.32 | 12.2 | 15.74 | 11.94 | 124.11 | 42.33 | 0.312 | 1 | 0.84 | 62.62 | 7 | 0 | 0 | 0 |
| 2 | 78.3 | 11.44 | 11.6 | 13.24 | 16.72 | 264.67 | 3.1 | 0.018 | 1 | 1.14 | 82.61 | 4 | 0 | 1 | 0 |
| 2 | 75.3 | 10.46 | 11.4 | 9.33 |  |  |  |  | 1 |  |  | 5 | 1 | 1 | 0 |
| 2 | 75.4 | 8.73 | 12.4 | 17.51 | 10.12 | 365.76 | 17.57 | 0.136 | 1 | 1.11 | 61.08 | 7 | 1 | 1 | 1 |
| 1 | 77.4 | 10.31 | 10.8 | 17.22 | 13.06 | 150.86 |  | 0.021 | 1 | 0.81 | 58.21 | 6 | 1 | 1 | 0 |
| 1 | 66.7 | 8.53 | 12.8 | 6.11 | 13.24 | 461.16 |  | 0.816 | 1 |  | 143.43 | 0 | 0 | 0 | 0 |
| 1 | 79.1 | 9.31 | 9.2 | 15.54 | 12.39 | 124.11 |  | 0.014 | 1 | 0.89 | 42.63 | 7 | 1 | 1 | 0 |
| 1 | 73.4 | 8.70 | 12.8 | 10.61 |  |  |  |  | 1 |  |  | 6 | 1 | 1 | 0 |
| 1 | 73.1 | 10.35 | 12.8 | 8.25 |  |  |  |  | 1 |  |  | 7 | 0 | 1 | 0 |
| 1 | 72.5 | 9.76 | 9.2 | 8.71 |  |  |  |  | 1 |  |  | 7 | 0 | 0 | 0 |
| 2 | 84 | 8.31 | 13.1 | 23.92 | 6.75 | 317.07 | 13.58 | 0.01 | 1 | 1.85 | 108.80 | 7 | 1 | 1 | 1 |
| 1 | 81 | 8.26 | 11.5 | 20.47 | 8.35 | 451.42 | 13.21 | 0.187 | 1 | 0.99 | 59.47 | 7 | 1 | 1 | 1 |
| 1 | 82 | 12.10 | 9.5 | 15.87 |  |  |  |  | 1 |  |  | 7 | 1 | 1 | 1 |
| 2 | 80 | 10.14 | 13.1 | 16.53 |  |  |  |  | 1 |  |  | 7 | 1 | 1 | 0 |
| 2 | 73.6 | 10.50 | 11.2 | 10.05 | 5.96 | 664.98 | 18.49 | 0.039 | 1 | 1.30 | 58.89 | 7 | 1 | 1 | 0 |
| 1 | 73 | 8.73 | 12.2 | 9.33 |  |  |  |  | 1 |  |  | 7 | 0 | 0 | 0 |
| 2 | 76 | 9.40 | 13 | 15.31 | 12.51 | 1050.14 | 32.3 | 0.247 | 1 | 2.17 | 84.27 | 7 | 0 | 1 | 0 |
| 2 | 76 | 8.92 | 13.8 | 18.96 | 24.99 |  | 24.35 | 0.051 | 1 | 1.21 | 75.52 | 7 | 0 | 0 | 0 |
| 1 | 74.8 | 11.42 | 11.2 | 13.96 | 12.73 | 327.66 | 4.17 | 0.121 | 1 | 1.32 | 62.99 | 7 | 1 | 1 | 0 |
| 2 | 72 | 8.27 | 12.1 | 13.5 |  |  |  |  | 1 |  |  | 7 | 1 | 1 | 0 |
| 1 | 72 | 7.65 | 11.2 | 12.85 |  |  |  |  | 1 |  |  | 7 | 0 | 1 | 0 |
| 1 | 80 | 8.96 | 11.6 | 20.86 | 16.01 | 687.17 | 8.98 | 0.015 | 1 | 1.44 | 70.91 | 7 | 1 | 1 | 0 |
| 1 | 80 | 10.69 | 13.5 | 19.61 | 13.42 | 902.14 | 13.94 | 0.052 | 1 | 1.38 | 54.17 | 7 | 0 | 1 | 0 |
| 1 | 74 | 8.21 | 12 | 12.55 | 18.76 | 758.29 | 31.08 | 0.042 | 1 | 1.62 | 74.54 | 7 | 1 | 1 | 0 |
| 1 | 80 | 10.25 | 14.4 | 20.6 | 15.53 | 790.14 | 26.49 | 0.131 | 1 | 1.20 | 53.16 | 7 | 1 | 1 | 1 |
| 2 | 80.5 | 9.95 | 12.8 | 20.01 | 15.81 | 364.11 | 25.19 | 0.036 | 1 | 1.04 | 63.15 | 7 | 1 | 1 | 0 |
| 2 | 74 | 8.66 | 11.7 | 14.49 | 19.17 | 648.81 | 11.85 | 0.013 | 1 | 1.11 | 58.31 | 6 | 0 | 1 | 0 |
| 1 | 79.5 | 9.92 | 11.5 | 21.16 | 8.31 | 275.51 | 17.2 | 0.025 | 1 | 1.05 | 38.87 | 7 | 1 | 1 | 1 |
| 2 | 77 | 10.03 | 12.5 | 15.7 | 27.58 | 837.15 | 33.82 | 0.034 | 1 | 2.26 | 87.87 | 6 | 1 | 1 | 0 |
| 2 | 81 | 9.67 | 12.8 | 21.95 | 11.38 | 1301.64 | 10.07 | 0.027 | 1 |  | 60.95 | 7 | 1 | 1 | 0 |
| 1 | 76 | 11.05 | 12.1 | 11.99 |  |  |  |  | 1 |  |  | 7 | 0 | 1 | 1 |
| 2 | 70 | 7.15 | 11.8 | 9.07 |  |  |  |  | 1 |  |  | 7 | 1 | 1 | 1 |
| 2 | 81 | 10.01 | 12.6 | 20.73 | 24.86 | 1446.25 | 57.81 | 0.01 | 1 | 1.26 | 65.69 | 7 | 1 | 1 | 1 |
| 1 | 82 | 10.51 | 12.9 | 18.79 | 14.49 | 493.5 | 60.25 | 0.019 | 1 |  | 76.03 | 7 | 1 | 1 | 0 |
| 2 | 92.5 | 12.88 | 13.6 | 23.89 |  | 487.3 | 11.53 | 0.011 | 1 | 1.69 | 53.79 | 7 | 0 | 0 | 1 |
| 1 | 76 | 9.28 | 12.7 | 16.56 | 11.38 | 338.48 | 52.39 |  | 1 | 1.26 | 101.66 | 7 | 1 | 1 | 0 |
| 1 | 76 | 9.53 | 11 | 12.39 |  |  |  |  | 1 |  |  | 6 | 1 | 0 | 0 |
| 1 | 76 | 10.54 | 13.3 | 11.86 | 32.05 | 1196.03 | 20.88 | 0.075 | 1 | 1.32 | 65.36 | 7 | 0 | 1 | 0 |
| 1 | 70 | 8.45 | 11.7 | 9.56 |  |  |  |  | 1 |  |  | 7 | 0 | 0 | 0 |
| 1 | 80 | 8.80 | 12.5 | 19.02 | 12.25 | 876.23 | 6.44 | 0.017 | 1 | 1.38 | 76.15 | 6 | 0 | 0 | 0 |
| 2 | 78 | 9.41 | 9.9 | 11.37 |  |  |  |  | 1 |  |  | 6 | 1 | 1 | 1 |
| 2 | 69 | 6.65 | 11.6 | 10.58 |  |  |  |  | 1 |  |  | 7 | 0 | 0 | 0 |
| 1 | 97 | 11.90 | 11.9 | 21.78 | 9.3 | 268.7 | 21.22 | 0.034 | 1 | 1.25 | 53.54 | 7 | 1 | 1 | 1 |
| 1 | 71 | 7.90 | 12.1 | 9.66 |  |  |  |  | 1 |  |  | 7 | 0 | 0 | 1 |
| 2 | 78 | 9.85 | 12.3 | 17.91 | 8.05 | 305.24 | 4.92 | 0.003 | 1 | 1.35 | 80.26 | 7 | 1 | 0 | 1 |
| 2 | 79 | 9.75 | 13.3 | 20.47 | 11.51 | 403.34 | 8.48 |  | 1 | 1.52 | 96.17 | 7 | 1 | 1 | 1 |
| 1 | 73 | 9.07 | 11.5 | 10.87 |  |  |  |  | 1 |  |  | 7 | 1 | 1 | 1 |
| 2 | 72 | 7.78 | 12 | 13.37 | 12.71 | 146.77 | 2.55 | 0.032 | 1 | 1.40 | 103.66 | 7 | 0 | 1 | 0 |
| 1 | 78 | 11.48 | 12.2 | 10.87 |  |  |  |  | 1 |  |  | 7 | 0 | 1 | 0 |
| 2 | 77 | 9.00 | 11.6 | 18.96 |  |  |  |  | 1 |  |  | 7 | 1 | 1 | 1 |
| 2 | 68 | 7.90 | 11.3 | 9.4 |  |  |  |  | 1 |  |  | 7 | 1 | 1 | 0 |
| 2 | 77 | 9.50 | 12.2 | 16.07 | 10.6 | 607.51 | 19.73 | 0.049 | 1 | 1.67 | 115.15 | 7 | 0 | 1 | 0 |
| 2 | 84 | 10.48 | 12.6 | 23.95 | 12.37 | 777.24 | 30.01 | 0.066 | 1 | 1.18 | 105.90 | 7 | 0 | 1 | 0 |
| 1 | 72 | 9.71 | 9 | 10.68 |  |  |  |  | 1 |  |  | 7 | 0 | 1 | 0 |
| 1 | 83 | 12.51 | 13.2 | 19.91 |  |  |  |  | 1 |  |  | 7 | 1 | 1 | 0 |
| 1 | 80 | 10.07 | 11.9 | 17.31 | 13.15 | 602.53 | 10.22 | 0.089 | 1 | 1.00 | 92.66 | 7 | 1 | 1 | 0 |
| 2 | 86 | 10.36 | 12.8 | 22.57 | 8.17 | 803.28 | 31.39 | 0.007 | 1 | 1.65 | 75.80 | 7 | 1 | 1 | 0 |
| 1 | 74.5 | 8.54 | 11.4 | 11.5 |  |  |  |  | 1 |  |  | 7 | 0 | 1 | 1 |
| 2 | 73 | 9.44 | 12.4 | 11.79 |  |  |  |  | 1 |  |  | 6 | 1 | 1 | 1 |
| 2 | 79 | 11.03 | 12.7 | 17.91 | 9.97 | 497.67 | 8.13 | 0.009 | 1 | 1.39 | 91.61 | 7 | 1 | 1 | 0 |
| 1 | 72 | 9.40 | 11.3 | 11.5 |  |  |  |  | 1 |  |  | 7 | 0 | 1 | 0 |
| 2 | 69 | 8.94 | 13.1 | 8.87 |  |  |  |  | 1 |  |  | 6 | 0 | 1 | 0 |
| 2 | 72 | 8.80 | 12.2 | 14.62 |  |  |  |  | 1 |  |  | 7 | 1 | 1 | 1 |
| 2 | 70 | 8.30 | 11.5 | 12.42 |  |  |  |  | 1 |  |  | 0 | 0 | 1 | 0 |
| 2 | 75.3 | 8.56 | 13.1 | 14.06 |  |  |  |  | 1 |  |  | 7 | 0 | 1 | 0 |
| 2 | 69.9 | 7.04 | 13.7 | 9.1 |  |  |  |  | 1 |  |  | 7 | 0 | 1 | 0 |
| 1 | 77 | 9.23 | 12.4 | 17.91 |  |  |  |  | 1 |  |  | 7 | 1 | 1 | 1 |
| 2 | 65 | 6.72 | 11.2 | 6.51 |  |  |  |  | 1 |  |  | 0 | 0 | 0 | 0 |
| 1 | 68 | 8.07 | 12.5 | 9 |  |  |  |  | 1 |  |  | 7 | 0 | 1 | 0 |
| 1 | 79.2 | 10.91 | 11 | 20.6 | 16.26 | 1453.01 | 45.9 | 0.013 | 1 | 1.28 | 68.09 | 6 | 1 | 1 | 1 |
| 2 | 73.5 | 8.71 | 11.8 | 13.14 |  |  |  |  | 1 |  |  | 6 | 1 | 1 | 1 |
| 2 | 70.2 | 8.82 | 11.5 | 10.15 |  |  |  |  | 1 |  |  | 1 | 1 | 1 | 0 |
| 1 | 76.1 | 10.10 | 13.1 | 12.65 |  |  |  |  | 1 |  |  | 6 | 0 | 0 | 0 |
| 1 | 83.2 | 11.60 | 11.1 | 17.22 |  |  |  |  | 1 |  |  | 7 | 1 | 1 | 1 |
| 2 | 85 | 11.36 | 12.5 | 23.52 | 19.03 | 465.11 | 18.39 | 0.023 | 1 | 1.31 | 81.19 | 7 | 0 | 1 | 0 |
| 1 | 80 | 9.82 | 11.3 | 20.73 |  | 489.36 | 20.17 | 0.022 | 1 | 0.91 | 79.96 | 7 | 1 | 1 | 1 |
| 2 | 79 | 11.78 | 14.4 | 20.6 | 20.82 | 646.15 | 11.04 | 0.011 | 1 | 1.83 | 91.56 | 7 | 0 | 1 | 0 |
| 1 | 76.5 | 12.43 | 12 | 15.87 | 7.72 | 208.07 | 10.86 | 0.007 | 1 | 0.91 |  | 7 | 1 | 1 | 0 |
| 1 | 68 | 7.86 | 10.4 | 6.11 |  |  |  |  | 1 |  |  | 0 | 0 | 0 | 0 |
| 2 | 82.5 | 10.65 | 13 | 19.09 | 23.18 | 174.09 | 24.7 | 0.01 | 1 | 1.01 |  | 6 | 0 | 1 | 0 |
| 1 | 72 | 8.11 | 12.3 | 6.7 |  |  |  |  | 1 |  |  | 1 | 0 | 0 | 0 |
| 1 | 70 | 7.63 | 11.7 | 8.8 |  |  |  |  | 1 |  |  | 4 | 1 | 1 | 0 |
| 1 | 82 | 10.31 | 12.6 | 22.11 | 30.59 | 861.79 | 27.64 | 0.009 | 1 | 1.29 | 96.09 | 7 | 1 | 1 | 1 |
| 2 | 75 | 9.82 | 12 | 12.78 |  |  |  |  | 1 |  |  | 7 | 1 | 1 | 0 |
| 1 | 82.5 | 10.05 | 12.1 | 18.63 | 37.13 | 876.23 | 17.38 | 0.019 | 1 | 1.89 | 84.82 | 6 | 0 | 1 | 0 |
| 1 | 79 | 9.66 | 12.2 | 15.01 | 41.74 | 1180.36 | 43.54 | 0.033 | 1 | 1.54 | 106.50 | 7 | 0 | 0 | 0 |
| 2 | 80 | 11.74 | 10.8 | 18.66 | 16.02 | 279.65 | 13.03 | 0.029 | 1 | 1.04 |  | 7 | 1 | 1 | 1 |
| 2 | 74.2 | 9.62 | 10.3 | 12.78 |  |  |  |  | 1 |  |  | 5 | 1 | 1 | 1 |
| 1 | 77.1 | 9.56 | 12.1 | 17.38 | 11.9 | 268.7 | 24.95 | 0.026 | 1 | 1.11 | 75.83 | 6 | 0 | 0 | 0 |
| 2 | 69.9 | 8.94 | 10.7 | 9.59 |  |  |  |  | 1 |  |  | 0 | 0 | 0 | 0 |
| 2 | 78.7 | 10.34 | 12.6 | 15.05 |  |  |  |  | 1 |  |  | 6 | 1 | 1 | 1 |
| 2 | 73.5 | 9.14 | 11.7 | 11.17 |  |  |  |  | 1 |  |  | 7 | 0 | 1 | 0 |
| 1 | 74.3 | 9.85 | 10.8 | 8.51 |  |  |  |  | 1 |  |  | 5 | 1 | 1 | 0 |
| 1 | 88.1 | 11.14 | 9.9 | 18.46 |  |  |  |  | 1 |  |  | 7 | 1 | 1 | 0 |
| 2 | 71.2 | 9.11 | 10.4 | 13.96 |  |  |  |  | 1 |  |  | 6 | 1 | 1 | 0 |
| 1 | 81 | 8.89 | 13.8 | 23.46 | 11.1 |  | 24.37 | 0.145 | 0 |  | 98.38 |  | 0 | 1 | 1 |
| 1 | 66 | 7.72 | 10.3 | 8.61 |  |  |  |  | 0 |  |  |  | 1 | 1 | 0 |
| 2 | 78.4 | 9.11 | 10.6 | 23.79 |  |  |  |  | 0 |  |  |  | 0 | 0 | 1 |
| 1 | 77 | 9.48 | 11.2 | 19.78 |  |  |  |  | 0 |  |  |  | 1 | 1 | 1 |
| 2 | 70.4 | 7.73 | 11.9 | 13.77 |  |  |  |  | 0 |  |  |  | 1 | 1 | 0 |
| 2 | 70 | 7.08 | 10.2 | 13.77 |  |  |  |  | 0 |  |  |  | 1 | 1 | 0 |
| 1 | 79.3 | 10.63 | 9.2 | 21.22 |  |  |  |  | 0 |  |  |  | 0 | 0 | 0 |
| 2 | 76 | 9.03 | 12 | 20.44 |  |  |  |  | 0 |  |  |  | 1 | 1 | 1 |
| 1 | 78 | 10.30 | 10.5 | 20.21 |  |  |  |  | 0 |  |  |  | 1 | 0 | 0 |
| 2 | 75 | 8.05 | 13.5 | 22.41 |  |  |  |  | 0 |  |  |  | 1 | 1 | 1 |
| 1 | 65 | 9.06 | 11.9 | 7.69 |  |  |  |  | 0 |  |  |  | 0 | 1 | 0 |
| 1 | 63 | 6.59 | 11.4 | 7.03 |  |  |  |  | 0 |  |  |  | 0 | 1 | 0 |
| 1 | 63 | 6.48 | 11.8 | 7.03 |  |  |  |  | 0 |  |  |  | 0 | 1 | 0 |
| 1 | 69 | 8.23 | 11.5 | 10.61 |  |  |  |  | 0 |  |  |  | 0 | 1 | 0 |
| 1 | 68 | 7.90 | 10.3 | 11.93 |  |  |  |  | 0 |  |  |  | 1 | 1 | 0 |
| 1 | 65 | 8.48 | 11.1 | 10.48 |  |  |  |  | 0 |  |  |  | 0 | 0 | 0 |
| 2 | 74.1 | 8.37 | 11.9 | 17.18 |  |  |  |  | 0 |  |  |  | 1 | 1 | 1 |
| 2 | 70 | 8.40 | 11.4 | 13.86 |  |  |  |  | 0 |  |  |  | 0 | 1 | 1 |
| 1 | 68 | 9.09 | 9.6 | 9.53 |  |  |  |  | 0 |  |  |  | 1 | 1 | 0 |
| 2 | 67 | 7.95 | 10.5 | 11.43 |  |  |  |  | 0 |  |  |  | 0 | 1 | 0 |
| 1 | 80 | 9.95 | 9 | 23.59 |  |  |  |  | 0 |  |  |  | 1 | 1 | 1 |
| 2 | 76 | 9.08 | 10.4 | 22.11 |  |  |  |  | 0 |  |  |  | 0 | 0 | 1 |
| 1 | 63 | 5.62 | 8.3 | 6.8 |  |  |  |  | 0 |  |  |  | 0 | 0 | 0 |
| 2 | 64 | 6.37 | 13.9 | 10.09 |  |  |  |  | 0 |  |  |  | 1 | 1 | 1 |
| 1 | 73 | 8.54 | 11.7 | 14.49 |  |  |  |  | 0 |  |  |  | 1 | 1 | 0 |
| 2 | 62 | 6.59 | 9.6 | 6.57 |  |  |  |  | 0 |  |  |  | 0 | 0 | 0 |
| 1 | 63.5 | 7.13 | 10.8 | 6.97 |  |  |  |  | 0 |  |  |  | 0 | 1 | 0 |
| 1 | 67 | 8.32 | 10.5 | 8.9 |  |  |  |  | 0 |  |  |  | 0 | 1 | 0 |
| 1 | 76 | 8.70 | 11.2 | 17.97 |  |  |  |  | 0 |  |  |  | 0 | 0 | 0 |
| 1 | 75.1 | 8.99 | 11.1 | 17.68 |  |  |  |  | 0 |  |  |  | 1 | 1 | 1 |
| 1 | 71.5 | 8.92 | 11.4 | 21.19 |  |  |  |  | 0 |  |  |  | 1 | 1 | 0 |
| 2 | 75 | 8.79 | 11.7 | 18.43 |  |  |  |  | 0 |  |  |  | 1 | 1 | 1 |
| 2 | 77 | 9.13 | 11.4 | 21.72 |  |  |  |  | 0 |  |  |  | 1 | 1 | 1 |
| 2 | 66.2 | 6.13 | 9.1 | 15.64 |  |  |  |  | 0 |  |  |  | 0 | 1 | 0 |
| 1 | 63 | 7.51 | 12 | 8.05 |  |  |  |  | 0 |  |  |  | 0 | 0 | 0 |
| 1 | 67 | 7.30 | 10.5 | 9.46 |  |  |  |  | 0 |  |  |  | 0 | 1 | 0 |
| 2 | 79 | 9.26 | 11.3 | 22.97 |  |  |  |  | 0 |  |  |  | 1 | 1 | 1 |
| 2 | 70 | 8.41 | 11.6 | 15.54 |  |  |  |  | 0 |  |  |  | 1 | 1 | 1 |
| 2 | 70.5 | 8.18 | 10.1 | 19.12 |  |  |  |  | 0 |  |  |  | 1 | 1 | 0 |
| 1 | 67 | 7.37 | 10.3 | 10.41 |  |  |  |  | 0 |  |  |  | 1 | 1 | 1 |
| 2 | 74.3 | 9.08 | 10.5 | 18.14 | 15.6 |  | 11.06 | 0.003 | 0 | 0.85 | 85.43 |  | 1 | 1 | 0 |
| 2 | 67 | 7.76 | 11 | 10.58 |  |  |  |  | 0 |  |  |  | 1 | 1 | 0 |
| 1 | 77 | 9.21 | 9.8 | 18.86 |  |  |  |  | 0 |  |  |  | 1 | 1 | 1 |
| 2 | 75 | 8.94 | 11.6 | 20.21 |  |  |  |  | 0 |  |  |  | 1 | 1 | 1 |
| 1 | 77.7 | 9.18 | 11.3 | 22.08 | 13.07 |  | 21.08 | 0.743 | 0 | 0.86 | 38.27 |  | 1 | 1 | 0 |
| 2 | 69 | 7.33 | 9.9 | 12.45 |  |  |  |  | 0 |  |  |  | 0 | 1 | 0 |
| 1 | 74.4 | 8.36 | 12.5 | 15.8 |  |  |  |  | 0 |  |  |  | 1 | 1 | 1 |
| 2 | 76 | 8.37 | 11.3 | 19.68 |  |  |  |  | 0 |  |  |  | 0 | 0 | 0 |
| 1 | 75 | 8.75 | 9.6 | 19.81 |  |  |  |  | 0 |  |  |  | 1 | 1 | 1 |
| 2 | 76 | 9.36 | 12.7 | 20.93 |  |  |  |  | 0 |  |  |  | 1 | 1 | 1 |
| 2 | 73 | 7.21 | 12.7 | 17.45 |  |  |  |  | 0 |  |  |  | 0 | 0 | 0 |
| 2 | 76 | 8.58 | 12.2 | 19.61 |  |  |  |  | 0 |  |  |  | 0 | 1 | 1 |
| 1 | 69 | 6.84 | 12 | 13.54 |  |  |  |  | 0 |  |  |  | 1 | 0 | 1 |
| 1 | 67 | 7.98 | 9.9 | 11.76 |  |  |  |  | 0 |  |  |  | 0 | 0 | 0 |
| 1 | 72 | 8.95 | 12.3 | 13.11 |  |  |  |  | 0 |  |  |  | 0 | 0 | 1 |
| 2 | 75 | 7.34 | 10.2 | 18.79 |  |  |  |  | 0 |  |  |  | 0 | 0 | 1 |
| 2 | 76 | 9.64 | 11.4 | 19.75 |  |  |  |  | 0 |  |  |  | 1 | 1 | 0 |
| 1 | 70 | 7.55 | 9.9 | 13.5 |  |  |  |  | 0 |  |  |  | 0 | 1 | 0 |
| 1 | 76 | 8.94 | 10.6 | 19.15 | 18.09 |  | 6.37 | 0.083 | 0 | 1.02 | 90.63 |  | 1 | 1 | 0 |
| 1 | 79 | 9.25 | 10.8 | 22.14 |  |  |  |  | 0 |  |  |  | 0 | 0 | 0 |
| 1 | 73 | 8.98 | 10.3 | 14.85 |  |  |  |  | 0 |  |  |  | 0 | 0 | 0 |
| 1 | 74 | 9.61 | 11.2 | 15.01 |  |  |  |  | 0 |  |  |  | 0 | 0 | 0 |
| 1 | 70 | 7.85 | 10.9 | 14.36 |  |  |  |  | 0 |  |  |  | 0 | 0 | 0 |
| 1 | 66 | 7.44 | 11.5 | 10.71 |  |  |  |  | 0 |  |  |  | 0 | 0 | 0 |
| 1 | 74 | 9.34 | 12.5 | 15.9 |  |  |  |  | 0 |  |  |  | 0 | 0 | 0 |
| 2 | 75 | 8.12 | 11.4 | 20.76 |  |  |  |  | 0 |  |  |  | 0 | 0 | 0 |
| 2 | 64 | 6.19 | 11.8 | 8.94 |  |  |  |  | 0 |  |  |  | 0 | 0 | 0 |
| 1 | 79.5 | 11.30 | 10.5 | 21.75 |  |  |  |  | 0 |  |  |  | 0 | 1 | 1 |
| 1 | 79.5 | 9.70 | 13.2 | 21.75 |  |  |  |  | 0 |  |  |  | 0 | 1 | 1 |
| 1 | 70 | 7.20 | 11.6 | 11.56 |  |  |  |  | 0 |  |  |  | 1 | 1 | 0 |
| 2 | 76 | 9.80 | 11.3 | 23.95 |  |  |  |  | 0 |  |  |  | 1 | 1 | 1 |
| 1 | 67.5 | 7.70 | 11.8 | 10.15 |  |  |  |  | 0 |  |  |  | 0 | 1 | 0 |
| 2 | 68 | 5.80 | 9.4 | 12.09 |  |  |  |  | 0 |  |  |  | 1 | 1 | 1 |
| 1 | 78 | 9.50 | 11.6 | 20.07 |  |  |  |  | 0 |  |  |  | 0 | 1 | 1 |
| 1 | 72 | 8.80 | 10.1 | 13.86 |  |  |  |  | 0 |  |  |  | 0 | 0 | 0 |
| 1 | 63.5 | 8.80 | 13 | 8.05 |  |  |  |  | 0 |  |  |  | 0 | 0 | 0 |
| 1 | 68.5 | 7.30 | 11.1 | 10.81 |  |  |  |  | 0 |  |  |  | 0 | 1 | 0 |
| 1 | 74 | 9.70 | 12.8 | 17.45 |  |  |  |  | 0 |  |  |  | 1 | 0 | 1 |
| 1 | 77 | 9.80 | 10.3 | 19.38 |  |  |  |  | 0 |  |  |  | 0 | 1 | 1 |
| 1 | 76.5 | 10.18 | 12.5 | 23.82 |  |  |  |  | 0 |  |  |  | 0 | 1 | 0 |
| 1 | 77 | 9.70 | 11.8 | 20.8 |  |  |  |  | 0 |  |  |  | 0 | 0 | 1 |
| 1 | 76 | 9.70 | 11 | 17.61 |  |  |  |  | 0 |  |  |  | 0 | 0 | 0 |
| 1 | 79.5 | 8.50 | 10.2 | 21.82 |  |  |  |  | 0 |  |  |  | 0 | 0 | 0 |
| 2 | 75 | 8.80 | 9.9 | 21.72 |  |  |  |  | 0 |  |  |  | 0 | 0 | 0 |
| 1 | 76 | 9.30 | 11.3 | 17.48 |  |  |  |  | 0 |  |  |  | 0 | 0 | 0 |
| 1 | 68 | 6.80 | 11.3 | 10.68 |  |  |  |  | 0 |  |  |  | 0 | 0 | 0 |
| 1 | 71 | 8.80 | 11.9 | 13.24 |  |  |  |  | 0 |  |  |  | 0 | 0 | 0 |
| 2 | 79.1 | 9.00 | 13.4 | 23.62 | 5.43 | 308.86 | 10.81 | 0.025 | 0 | 1.04 | 106.34 |  | 0 | 1 | 0 |
| 2 | 77 | 9.42 | 12.3 | 20.76 | 7.73 | 468.06 | 6.14 | 0.003 | 0 | 0.99 | 51.67 |  | 0 | 0 | 1 |
| 1 | 62.5 | 6.76 | 13.1 | 6.11 |  |  |  |  | 0 |  |  |  | 0 | 0 | 0 |
| 2 | 71.5 | 7.69 | 12.6 | 14.49 |  |  | 11.2 | 0.025 | 0 |  | 60.30 |  | 1 | 1 | 0 |
| 1 | 80.2 | 8.57 | 12.2 | 23.75 | 13.16 | 356.27 | 28.27 | 0.405 | 0 | 0.81 | 92.69 |  | 1 | 1 | 1 |
| 1 | 71.9 | 7.67 | 11.9 | 13.4 | 17.66 | 398.49 | 11.92 | 0.002 | 0 | 0.82 | 76.01 |  | 1 | 1 | 1 |
| 1 | 77.1 | 8.20 | 12.4 | 23.1 | 16.81 | 482.9 | 20.19 | 0.002 | 0 | 0.88 | 76.88 |  | 0 | 0 | 1 |
| 2 | 66.2 | 6.92 | 11.2 | 9.99 |  |  |  |  | 0 |  |  |  | 0 | 1 | 0 |
| 1 | 71.7 | 8.13 | 11.6 | 14.98 | 10.98 | 262.4 | 11.76 | 0.005 | 0 | 0.84 | 66.98 |  | 1 | 1 | 0 |
| 1 | 76.5 | 9.09 | 11 | 19.09 | 7.57 | 342.15 | 4.5 | 0.002 | 0 | 1.18 | 66.98 |  | 0 | 1 | 0 |
| 1 | 73.5 | 9.22 | 11 | 15.54 | 11.38 | 243.15 | 13.47 | 0.002 | 0 | 1.05 | 88.87 |  | 0 | 1 | 0 |
| 2 | 70.2 | 8.90 | 13.6 | 15.74 |  |  |  |  | 0 |  |  |  | 0 | 0 | 1 |
| 1 | 74.2 | 7.67 | 11.3 | 15.34 | 10.99 | 366.77 | 2.52 | 0.003 | 0 | 1.15 | 90.48 |  | 1 | 1 | 1 |
| 1 | 71 | 8.70 | 11.7 | 13.14 |  |  | 7.92 | 0.002 | 0 | 1.64 | 64.86 |  | 0 | 0 | 0 |
| 1 | 68 | 9.10 | 11.1 | 11.2 |  |  |  |  | 0 |  |  |  | 0 | 0 | 1 |
| 1 | 70 | 9.19 | 11.9 | 12.16 |  |  |  |  | 0 |  |  |  | 0 | 1 | 0 |
| 2 | 76 | 8.63 | 12.4 | 23.89 | 10.44 | 189.44 | 16.72 | 0.002 | 0 | 1.14 | 85.99 |  | 1 | 1 | 1 |
| 2 | 60.4 | 5.19 | 11.2 | 6.6 |  |  |  |  | 0 |  |  |  | 0 | 1 | 0 |
| 2 | 78 | 8.90 | 14.3 | 21.68 | 13.04 | 364.13 | 6.35 | 0.04 | 0 | 0.88 | 82.74 |  | 1 | 1 | 1 |
| 2 | 67 | 6.51 | 11.8 | 13.93 | 7.31 | 544.42 | 22.82 | 0.048 | 0 | 0.85 | 65.40 |  | 0 | 0 | 1 |
| 1 | 74.5 | 7.60 | 11.8 | 17.91 | 11.13 | 114.06 | 14.91 | 0.004 | 0 | 0.87 | 101.48 |  | 1 | 1 | 1 |
| 1 | 72 | 8.00 | 13 | 15.28 | 16.19 | 586.19 | 17.88 | 0.003 | 0 | 0.99 | 114.87 |  | 1 | 1 | 1 |
| 1 | 68.5 | 6.03 | 11.6 | 10.12 |  |  |  |  | 0 |  |  |  | 0 | 1 | 0 |
| 2 | 68.5 | 8.30 | 11.7 | 11.79 |  |  |  |  | 0 |  |  |  | 0 | 1 | 0 |
| 1 | 70 | 7.28 | 12 | 14.03 | 10.23 | 261.83 | 6.91 | 0.002 | 0 | 1.09 | 68.13 |  | 0 | 1 | 0 |
| 1 | 70.5 | 9.70 | 11.4 | 12.98 | 6.63 | 162.4 | 21 | 0.002 | 0 | 1.03 | 87.73 |  | 0 | 0 | 1 |
| 2 | 73.1 | 8.32 | 13.3 | 20.27 | 35.89 | 300.08 | 30.7 | 0.002 | 0 | 1.07 | 66.13 |  | 0 | 1 | 0 |
| 2 | 77 | 9.70 | 13.1 | 20.9 | 3.62 | 420.69 | 10.93 | 0.002 | 0 | 1.08 | 63.65 |  | 0 | 1 | 0 |
| 1 | 70 | 7.80 | 12.7 | 12.45 |  |  |  |  | 0 |  |  |  | 0 | 1 | 0 |
| 1 | 78.4 | 9.79 | 12.2 | 22.37 | 12.37 | 155.54 | 12.97 | 0.002 | 0 | 1.21 | 102.66 |  | 0 | 1 | 0 |
| 2 | 76.5 | 8.50 | 13.4 | 21.03 | 12.47 | 164.22 | 25.53 | 0.003 | 0 | 1.26 | 91.95 |  | 1 | 0 | 1 |
| 1 | 80.5 | 10.60 | 12.6 | 23.56 |  |  | 4.79 | 0.168 | 0 | 0.89 | 75.50 |  | 0 | 0 | 0 |
| 1 | 77 | 9.00 | 12.5 | 19.15 | 7.52 | 426.62 | 20.03 | 0.003 | 0 | 1.03 | 122.69 |  | 1 | 1 | 1 |
| 1 | 73.5 | 9.45 | 11.2 | 17.91 | 10.13 | 225.88 | 29.87 | 0.002 | 0 | 1.23 | 92.48 |  | 0 | 0 | 1 |
| 2 | 78.2 | 10.10 | 11.7 | 22.08 | 6.88 | 327.12 | 13.36 | 0.003 | 0 | 0.98 | 97.37 |  | 0 | 0 | 0 |
| 1 | 79.3 | 10.30 | 11.2 | 21.03 | 12.37 | 373.42 | 41.56 | 0.04 | 0 | 0.91 | 112.36 |  | 1 | 0 | 1 |
| 1 | 75.5 | 9.83 | 9.9 | 16.62 | 7.56 | 453.47 | 6.18 | 0.011 | 0 | 0.96 | 98.02 |  | 0 | 1 | 0 |
| 1 | 69 | 8.00 | 10.3 | 10.97 |  |  |  |  | 0 |  |  |  | 0 | 0 | 0 |
| 1 | 70 | 6.90 | 11.3 | 13.04 | 11.61 | 84.3 | 4.2 | 0.002 | 0 | 0.75 | 88.12 |  | 0 | 0 | 0 |
| 1 | 66.5 | 6.10 | 11.5 | 8.25 |  |  |  |  | 0 |  |  |  | 1 | 1 | 1 |
| 1 | 74 | 9.10 | 11.2 | 15.41 |  | 503.56 | 11.45 | 0.517 | 0 | 0.71 | 89.67 |  | 0 | 1 | 1 |
| 2 | 70 | 6.80 | 11.3 | 14.29 |  |  | 16.57 | 0.495 | 0 | 0.93 | 121.48 |  | 0 | 1 | 0 |
| 2 | 79 | 8.90 | 12.4 | 23.46 | 4.85 | 479.15 | 13.87 | 0.028 | 0 | 1.26 | 35.62 |  | 1 | 1 | 0 |
| 2 | 68.5 | 8.10 | 12.6 | 12.65 |  |  |  |  | 0 |  |  |  | 1 | 0 | 1 |
| 2 | 71 | 8.90 | 12.3 | 14.46 | 19.6 | 121.38 | 17.76 | 0.043 | 0 | 1.10 | 70.88 |  | 0 | 0 | 0 |
| 1 | 76 | 9.70 | 12.5 | 23.89 |  |  | 12 |  | 0 | 1.12 | 96.20 |  | 0 | 1 | 1 |
| 2 | 75.5 | 8.80 | 13 | 20.14 |  |  |  |  | 0 | 1.02 |  |  | 0 | 0 | 1 |
| 1 | 73.5 | 8.60 | 13.2 | 16.99 | 5.68 | 307.2 | 9.25 | 0.002 | 0 | 1.08 | 85.78 |  | 1 | 0 | 1 |
| 2 | 78.2 | 9.07 | 12 | 23.1 |  |  | 17.24 | 0.004 | 0 | 0.95 |  |  | 1 | 1 | 0 |
| 2 | 63.1 | 6.82 | 11.2 | 7.46 |  |  |  |  | 0 |  |  |  | 0 | 1 | 0 |
| 1 | 67.6 | 8.11 | 11.3 | 10.41 |  |  |  |  | 0 |  |  |  | 1 | 1 | 0 |
| 1 | 69.1 | 8.33 | 11.8 | 13.04 |  |  |  |  | 0 |  |  |  | 0 | 1 | 0 |
| 1 | 68.1 | 7.75 | 11.1 | 13.04 |  |  |  |  | 0 |  |  |  | 0 | 1 | 0 |
| 1 | 76.3 | 9.24 | 12.1 | 17.74 |  |  | 20.62 | 0.005 | 0 | 1.07 |  |  | 1 | 1 | 1 |
| 1 | 70.8 | 7.92 | 12.9 | 13.17 |  |  | 38.02 | 0.003 | 0 | 1.17 |  |  | 1 | 1 | 0 |
| 1 | 68.2 | 6.57 | 12.7 | 16.89 |  |  |  |  | 0 |  |  |  | 0 | 1 | 0 |
| 1 | 76.3 | 10.06 | 11.9 | 17.84 |  |  | 4.9 | 0.003 | 0 |  |  |  | 1 | 1 | 0 |
| 1 | 79 | 9.71 | 13 | 21.09 |  |  | 8.93 | 0.003 | 0 |  |  |  | 0 | 1 | 0 |
| 2 | 79.1 | 10.17 | 9.8 | 23 |  |  | 69.67 | 0.06 | 0 |  |  |  | 1 | 1 | 1 |
| 1 | 78.8 | 9.17 | 12 | 20.34 |  |  | 13.93 | 0.04 | 0 |  |  |  | 1 | 1 | 1 |
| 1 | 76.4 | 8.78 | 11.8 | 22.51 |  |  | 16.76 | 0.353 | 0 |  |  |  | 1 | 1 | 1 |
| 1 | 68 | 8.04 | 11.3 | 10.02 |  |  |  |  | 1 |  |  | 0 | 1 | 1 | 1 |
| 1 | 65.8 | 7.07 | 12.4 | 9.69 |  |  |  |  | 1 |  |  | 7 | 1 | 1 | 1 |
| 1 | 70.4 | 8.98 | 13.2 | 11.99 |  |  |  |  | 1 |  |  | 6 | 1 | 0 | 0 |
| 1 | 74.5 | 8.70 | 12.8 | 17.12 |  |  |  |  | 1 |  |  | 7 | 1 | 1 | 0 |
| 2 | 65 | 7.27 | 9 | 8.8 |  |  |  |  | 1 |  |  | 0 | 0 | 1 | 1 |
| 1 | 65 | 7.38 | 10.3 | 7.23 |  |  |  |  | 1 |  |  | 0 | 0 | 1 | 0 |
| 1 | 70 | 9.16 | 10.7 | 11.2 |  |  |  |  | 1 |  |  | 0 | 0 | 1 | 0 |
| 1 | 70 | 9.26 | 11.5 | 11.4 |  |  |  |  | 1 |  |  | 0 | 0 | 1 | 0 |
| 1 | 64 | 7.04 | 10.8 | 7.06 |  |  |  |  | 1 |  |  | 0 | 1 | 1 | 0 |
| 2 | 67 | 7.34 | 8.5 | 11.27 |  |  |  |  | 1 |  |  | 7 | 0 | 0 | 0 |
| 2 | 67 | 7.62 | 8.5 | 11.27 |  |  |  |  | 1 |  |  | 7 | 0 | 0 | 0 |
| 1 | 70 | 7.90 | 11.7 | 11.4 |  |  |  |  | 1 |  |  | 6 | 0 | 1 | 0 |
| 1 | 70 | 9.70 | 10.7 | 11.96 |  |  |  |  | 1 |  |  | 4 | 1 | 1 | 1 |
| 1 | 71 | 9.87 | 9.9 | 19.61 |  |  |  |  | 1 |  |  | 0 | 1 | 1 | 0 |
| 1 | 74.5 | 9.25 | 9.8 | 17.12 |  |  |  |  | 1 |  |  | 0 | 0 | 1 | 0 |
| 1 | 76 | 10.07 | 10.9 | 20.44 |  |  |  |  | 1 |  |  | 0 | 0 | 1 | 0 |
| 2 | 73 | 9.70 | 11.4 | 16.72 |  |  |  |  | 1 |  |  | 0 | 1 | 1 | 0 |
| 1 | 78 | 9.09 | 12.6 | 21.91 |  |  |  |  | 1 |  |  | 7 | 0 | 1 | 0 |
| 2 | 68 | 7.96 | 11.4 | 12.19 |  |  |  |  | 1 |  |  | 5 | 1 | 1 | 0 |
| 2 | 72 | 8.70 | 10.7 | 16.46 |  |  |  |  | 1 |  |  | 7 | 1 | 1 | 1 |
| 1 | 78 | 10.10 | 12.1 | 20.73 | 18.35 | 480.22 | 11.83 | 0.018 | 1 | 1.34 | 36.25 | 7 | 0 | 1 | 0 |
| 1 | 74 | 8.83 | 11.6 | 15.05 | 8.29 | 91.95 | 14.16 | 0.016 | 1 | 1.23 | 78.55 | 0 | 1 | 1 | 0 |
| 1 | 76.5 | 9.92 | 11.3 | 18.17 | 20.48 | 909.21 | 6.58 |  | 1 | 1.24 | 42.63 | 7 | 1 | 1 | 1 |
| 1 | 78 | 9.42 | 11.8 | 23.06 | 17.15 | 243.23 | 34.83 |  | 1 | 1.77 | 42.15 | 6 | 0 | 1 | 0 |
| 1 | 78.5 | 10.04 | 12.8 | 20.27 |  |  |  |  | 1 | 1.15 | 59.79 | 7 | 1 | 1 | 1 |
| 1 | 78 | 9.94 | 12.3 | 23.69 | 15.62 | 174.47 | 30.1 | 0.012 | 1 | 1.31 | 54.50 | 7 | 1 | 1 | 0 |
| 1 | 76 | 9.47 | 12.2 | 18.79 | 8.1 | 232.06 | 6.11 | 0.007 | 1 | 0.99 | 43.61 | 0 | 1 | 1 | 1 |
| 1 | 76 | 10.37 | 11.1 | 17.05 | 10.86 | 69.36 | 14.69 | 0.098 | 1 | 0.90 | 46.41 | 0 | 1 | 1 | 0 |
| 2 | 71 | 7.66 | 10.1 | 14.19 | 18.63 | 112.07 | 54.48 | 3.122 | 1 | 0.63 | 40.76 | 0 | 0 | 1 | 0 |
| 1 | 79 | 10.59 | 10.1 | 21.45 | 11.24 | 341.61 | 3.15 | 0.048 | 1 | 0.93 | 70.00 | 7 | 1 | 1 | 1 |
| 2 | 73.5 | 9.07 | 13.1 | 16.72 | 34.85 | 990.05 | 28.07 | 0.026 | 1 | 1.52 | 72.47 | 7 | 0 | 1 | 0 |
| 1 | 73.5 | 9.10 | 11 | 15.38 | 18.99 | 178.82 | 9.08 | 0.021 | 1 | 1.17 | 55.31 | 0 | 0 | 1 | 0 |
| 1 | 78 | 10.14 | 11.8 | 21.95 | 7.36 | 520.85 | 10.92 | 0.058 | 1 | 1.04 | 40.23 | 0 | 0 | 0 | 0 |
| 1 | 76 | 9.51 | 10.5 | 17.68 | 15.52 | 179.92 | 3.57 | 0.067 | 1 | 0.68 | 50.24 | 0 | 0 | 1 | 0 |
| 1 | 75 | 9.68 | 11.9 | 18.6 | 12.99 | 228.4 | 19.7 | 0.174 | 1 | 1.18 | 40.99 | 0 | 1 | 1 | 0 |
| 1 | 72 | 8.73 | 12.3 | 13.57 | 21.9 | 448.85 |  |  | 1 | 1.26 |  | 7 | 0 | 0 | 0 |
| 1 | 74.7 | 8.77 | 11.6 | 21.36 | 25.5 | 388.35 | 52.13 | 0.02 | 1 | 1.07 | 20.72 | 7 | 0 | 1 | 0 |
| 2 | 74 | 10.43 | 11.6 | 18.73 | 9.46 | 234.16 | 2.6 | 0.008 | 1 | 1.54 | 30.50 | 7 | 1 | 1 | 1 |
| 2 | 71 | 8.24 | 13.8 | 14.85 | 15.01 | 450.79 | 12.87 | 0.078 | 1 | 1.19 | 34.23 | 7 | 0 | 1 | 0 |
| 1 | 66.3 | 6.59 | 13.2 | 12.19 | 18.18 | 231.59 | 27.3 | 0.017 | 1 | 0.97 | 46.56 | 6 | 1 | 1 | 1 |
| 1 | 77 | 7.46 | 11.7 | 22.47 | 17.16 | 542.77 | 14.97 | 0.087 | 1 |  | 24.91 | 7 | 1 | 1 | 1 |
| 2 | 68 | 8.63 | 12.6 | 15.41 | 24.89 | 529.39 | 8.4 | 0.012 | 1 | 1.23 | 46.86 | 6 | 1 | 1 | 1 |
| 1 | 77 | 10.10 | 12.8 | 18.37 | 11.11 | 361.21 | 14.68 | 0.138 | 1 | 1.16 | 43.06 | 7 | 1 | 1 | 1 |
| 2 | 70 | 7.35 | 11 | 14.03 | 5.81 | 124.89 | 12.68 | 0.033 | 1 | 0.91 | 43.16 | 0 | 1 | 1 | 0 |
| 1 | 77 | 10.30 | 13 | 21.49 | 10.18 | 601.71 | 21.1 | 0.042 | 1 | 1.67 | 29.54 | 7 | 1 | 0 | 1 |
| 1 | 76 | 9.80 | 13.3 | 18.86 | 14.88 | 159.46 | 13.87 | 0.013 | 1 | 1.98 | 30.70 | 7 | 1 | 1 | 1 |
| 2 | 74 | 9.30 | 12.7 | 19.55 | 16.65 | 107.2 | 9.21 | 0.008 | 1 | 1.77 | 47.64 | 7 | 1 | 1 | 1 |
| 2 | 77.6 | 9.25 | 11 | 21.49 | 12.41 | 433.59 |  | 13.756 | 1 |  | 52.51 | 7 | 0 | 0 | 0 |
| 2 | 72 | 8.35 | 12.1 | 15.28 | 15.99 | 507.71 | 8.95 | 0.021 | 1 | 1.73 | 55.73 | 7 | 1 | 0 | 1 |
| 1 | 79 | 10.00 | 13.4 | 20.67 | 14.31 | 520.63 | 14.44 | 0.106 | 1 | 1.41 | 93.98 | 7 | 1 | 0 | 1 |
| 2 | 77 | 10.15 | 12.2 | 21.03 | 7.57 | 985.82 | 11.74 | 0.222 | 1 | 1.27 | 64.89 | 6 | 1 | 1 | 1 |
| 1 | 70 | 9.85 | 12.3 | 17.41 | 13.33 | 372.92 | 17.34 | 0.019 | 1 | 1.11 | 64.46 | 7 | 1 | 1 | 1 |
| 1 | 76 | 8.65 | 12.4 | 17.87 | 11.36 | 733.78 | 63.55 | 0.191 | 1 | 1.11 | 69.40 | 6 | 1 | 1 | 0 |
| 2 | 72.7 | 8.41 | 11 | 16.62 | 6.06 | 684.35 | 17.22 | 0.013 | 1 | 1.47 | 40.23 | 7 | 0 | 1 | 0 |
| 2 | 70.2 | 9.32 | 13.1 | 19.09 | 11.59 | 670.46 | 6.26 | 0.159 | 1 | 1.14 | 46.61 | 7 | 1 | 1 | 1 |
| 2 | 70.3 | 9.32 | 11.7 | 19.09 |  |  |  |  | 1 | 1.02 |  | 7 | 1 | 1 | 1 |
| 1 | 77.2 | 9.27 | 10.4 | 20.3 |  |  |  |  | 1 |  |  | 0 | 1 | 1 | 1 |
| 1 | 74.5 | 10.75 | 9.5 | 19.09 | 15.75 | 718.88 | 17.7 | 0.014 | 1 | 0.84 | 65.14 | 6 | 0 | 1 | 1 |
| 2 | 73.6 | 7.86 | 11.5 | 17.97 | 15.97 | 1027.97 | 48.39 | 0.639 | 1 | 0.83 | 59.77 | 7 | 1 | 1 | 1 |
| 1 | 69.1 | 7.38 | 9.9 | 12.62 |  |  |  |  | 1 |  |  | 7 | 1 | 1 | 0 |
| 2 | 70.7 | 9.99 | 13.5 | 19.48 | 11.39 | 956.62 | 20.42 | 0.015 | 1 | 2.04 | 67.41 | 7 | 0 | 1 | 0 |
| 1 | 76.7 | 11.04 | 12.1 | 21.03 |  |  |  |  | 1 |  |  | 6 | 1 | 1 | 1 |
| 1 | 81.3 | 10.34 | 12.6 | 23.75 | 18.47 | 718.88 | 16.98 | 0.03 | 1 | 1.88 | 62.59 | 7 | 1 | 1 | 0 |
| 1 | 70.1 | 7.90 | 11.6 | 14.98 | 16.69 |  | 20.34 | 0.242 | 1 | 1.86 | 106.23 | 7 | 0 | 1 | 0 |
| 2 | 67.2 | 8.12 | 13.7 | 14.95 |  |  |  |  | 1 |  |  | 7 | 1 | 1 | 0 |
| 1 | 74.3 | 9.76 | 11.4 | 15.34 |  |  |  |  | 1 | 0.81 |  | 7 | 1 | 1 | 0 |
| 1 | 74 | 8.36 | 12.9 | 15.97 | 16.5 | 243.71 | 3.86 | 0.017 | 1 | 0.84 | 57.07 | 7 | 1 | 1 | 1 |
| 1 | 75 | 9.64 | 12.3 | 17.48 | 12.85 | 627.84 | 30.74 | 0.016 | 1 | 1.38 | 88.76 | 7 | 1 | 1 | 1 |
| 2 | 72.8 | 8.16 | 11.6 | 17.25 | 14.57 | 152.91 | 2.31 | 0.013 | 1 | 0.89 | 47.34 | 7 | 1 | 0 | 1 |
| 1 | 64 | 8.81 | 12.1 | 6.47 |  |  |  |  | 1 |  |  | 0 | 0 | 1 | 0 |
| 1 | 65.6 | 7.33 | 10.9 | 10.74 |  |  |  |  | 1 |  |  | 7 | 1 | 1 | 1 |
| 1 | 79 | 9.65 | 12.3 | 23.92 | 14.63 | 816.65 | 44.98 | 0.061 | 1 |  | 61.43 | 7 | 1 | 1 | 0 |
| 1 | 76.5 | 11.04 | 12.9 | 18.5 |  |  |  |  | 1 |  |  | 7 | 1 | 1 | 1 |
| 1 | 73.9 | 9.02 | 11.3 | 15.87 | 8.56 | 905.92 | 21.56 | 0.045 | 1 | 0.98 | 37.81 | 7 | 0 | 0 | 1 |
| 1 | 68 | 7.72 | 11.5 | 10.12 |  |  |  |  | 1 |  |  | 6 | 0 | 1 | 0 |
| 2 | 61 | 6.65 | 11.3 | 6.41 |  |  |  |  | 1 |  |  | 0 | 0 | 0 | 0 |
| 2 | 70 | 8.52 | 12.5 | 13.47 | 12.14 | 296.56 | 20.2 | 0.174 | 1 | 0.69 | 42.50 | 7 | 1 | 1 | 0 |
| 1 | 65 | 8.16 | 11.9 | 7.92 |  |  |  |  | 1 |  |  | 7 | 1 | 1 | 0 |
| 1 | 65.5 | 8.26 | 11.9 | 7.92 |  |  |  |  | 1 |  |  | 7 | 1 | 1 | 0 |
| 2 | 62 | 7.85 | 11 | 7.16 |  |  |  |  | 1 |  |  | 0 | 0 | 0 | 0 |
| 1 | 63 | 6.55 | 12.6 | 7 |  |  |  |  | 1 |  |  | 7 | 0 | 0 | 0 |
| 1 | 68 | 7.10 | 10 | 9.49 |  |  |  |  | 1 |  |  | 5 | 0 | 0 | 0 |
